# Microbiome Geographic Population Structure (mGPS) Detects Fine-Scale Geography

**DOI:** 10.1093/gbe/evae209

**Published:** 2024-10-07

**Authors:** Yali Zhang, Leo McCarthy, Emil Ruff, Eran Elhaik

**Affiliations:** Department of Biology, Lund University, Lund 22362, Sweden; Department of Mathematics, Sheffield University, Sheffield S3 7RH, UK; Ecosystems Center, Marine Biological Laboratory, Woods Hole, MA, USA; Department of Biology, Lund University, Lund 22362, Sweden

**Keywords:** microbiome, biogeographical predictions, microbiome geographic population structure (mGPS), antimicrobial resistance (AMR), forensics, machine learning

## Abstract

Over the past decade, sequencing data generated by large microbiome projects showed that taxa exhibit patchy geographical distribution, raising questions about the geospatial dynamics that shape natural microbiomes and the spread of antimicrobial resistance genes. Answering these questions requires distinguishing between local and nonlocal microorganisms and identifying the source sites for the latter. Predicting the source sites and migration routes of microbiota has been envisioned for decades but was hampered by the lack of data, tools, and understanding of the processes governing biodiversity. State-of-the-art biogeographical tools suffer from low resolution and cannot predict biogeographical patterns at a scale relevant to ecological, medical, or epidemiological applications. Analyzing urban, soil, and marine microorganisms, we found that some taxa exhibit regional-specific composition and abundance, suggesting they can be used as biogeographical biomarkers. We developed the microbiome geographic population structure, a machine learning–based tool that utilizes microbial relative sequence abundances to yield a fine-scale source site for microorganisms. Microbiome geographic population structure predicted the source city for 92% of the samples and the within-city source for 82% of the samples, though they were often only a few hundred meters apart. Microbiome geographic population structure also predicted soil and marine sampling sites for 86% and 74% of the samples, respectively. We demonstrated that microbiome geographic population structure differentiated local from nonlocal microorganisms and used it to trace the global spread of antimicrobial resistance genes. Microbiome geographic population structure's ability to localize samples to their water body, country, city, and transit stations opens new possibilities in tracing microbiomes and has applications in forensics, medicine, and epidemiology.

## Graphical Abstract

**Graphical Abstract:**
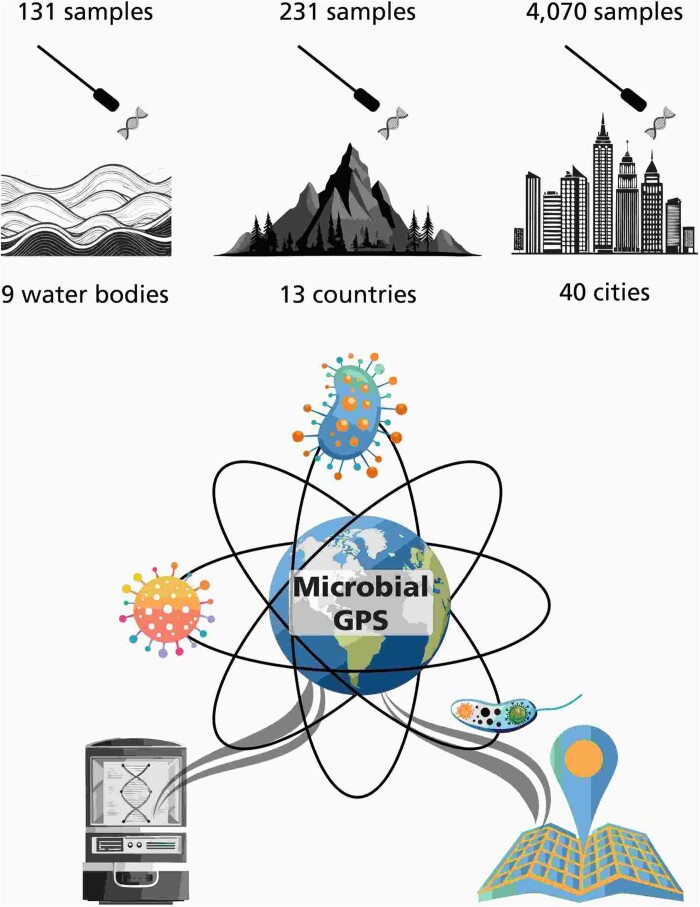

**Significance**

Predicting the geographical origins of microbial communities is critical for studying their biogeography and the transmission of antimicrobial resistance (AMR) genes, but suitable methods are lacking. We developed the microbiome geographic population structure (mGPS), which utilizes microbial relative sequence abundances to predict the source sites of microbiome samples and trace the spread of AMR. The ability of mGPS to accurately localize samples independently of the sequencing method and environment opens new avenues of microbiome research with applications in forensics, medicine, and epidemiology.

## Introduction

Trace evidence analysis is a specialized discipline within forensic science that focuses on the examination and interpretation of minute transfers of materials between objects, individuals, and environments. This field encompasses the detection, collection, and analysis of a wide range of small pieces of evidence (traces) that can link a person to a particular location, object, or event. These evidences can include hair, fibers, soil, pollen, glass, paint, or gunshot residue. Tracing the most recent geographic whereabouts of individual organisms has always been a major challenge in fields like ecology (Ricklefs and Jenkins 2011), microbiology (Peay et al. 2016), and forensics (Elhaik et al. 2021) due to the physical difficulties in identifying biological material that can uniquely associate individuals with the sites they visited or the objects they touch. Recent advances in biogeography showed that human DNA can be used to make accurate predictions of their country and, in some cases, village of origins (Elhaik et al. 2014). However, biotracing human DNA has limited forensic value because it remains constant and does not provide information about an individual's most recent movements (Mason-Buck et al. 2020). Only dynamic spatiotemporal types of information shared and exchanged between organisms and the environment can be used by tracing applications. The complex communities of bacteria, fungi, viruses, and microeukaryotes (microbiomes)—integral parts of natural ecosystems—represent the kind of information that may be informative to identify changes in one's environment.

**Fig. 1.**
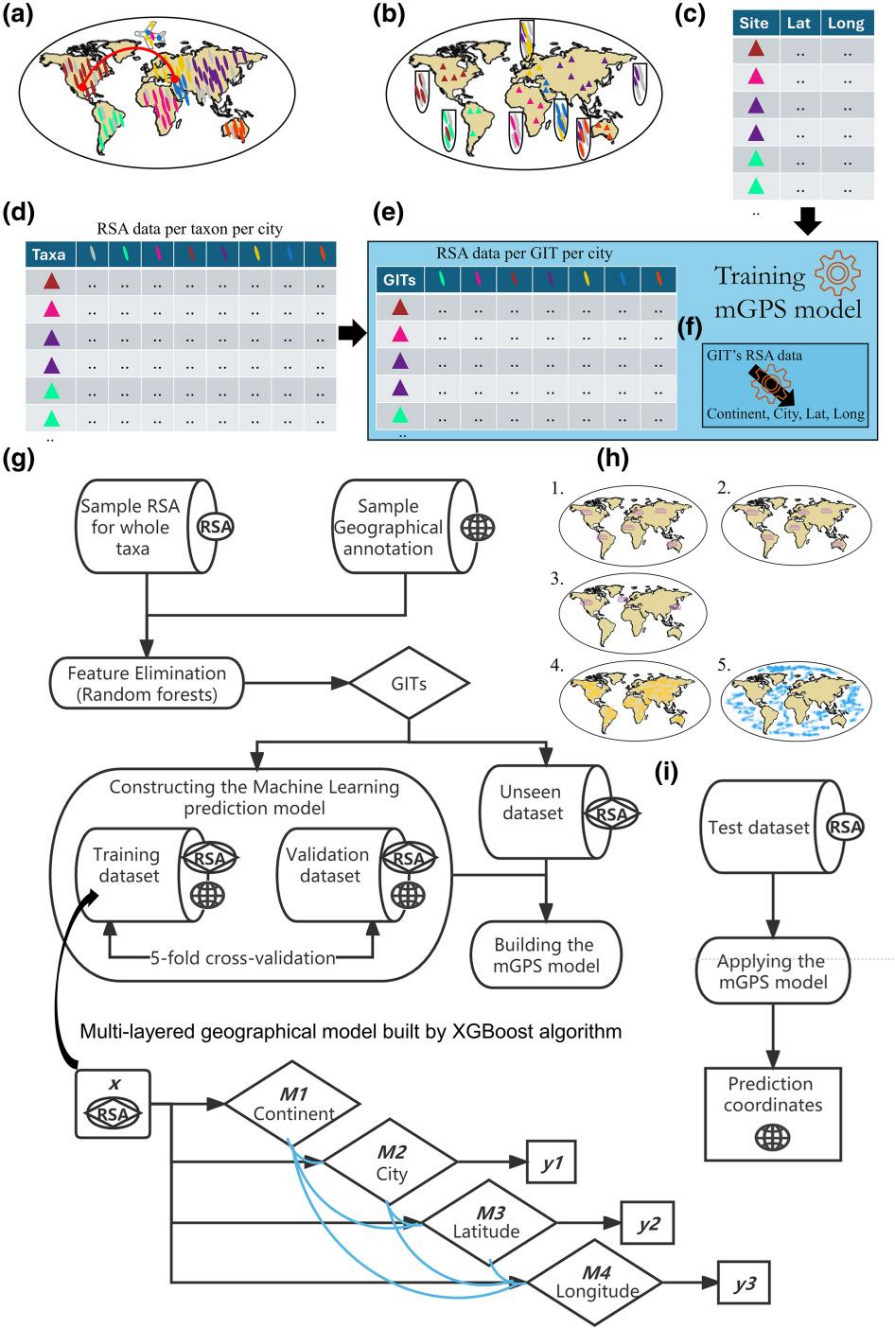
Schematic overview of the ecological microbiome model and mGPS's workflow. a) Microbiomes are characterized by taxa with local (color) and global (gray) dispersion. Human and natural activities mix these taxa, yet it is unclear to what extent the local taxa can survive and adapt to their new locations. b) Sampling can be done to identify the taxa of each site (triangles) and record its metadata (e.g. country and city) and geographical coordinates c). Following sequencing, the RSA of each taxon can be calculated d). The coordinates of the sampling sites and the RSA of the taxa are used to train the mGPS model. mGPS starts by identifying the geographically localized taxa e) and learning the relationships between their RSA and geography. The final mGPS model f) can then be used to convert any similarly-processed RSA taxa data into precise geographical locations. The mGPS model is detailed in g). First, GITs are selected after a random forest–based feature elimination step computes the RSA data of taxa with geographic information. The RSAs of GITs are used in the model training of XGBoost. The prediction model consists of chained submodels trained step-by-step using multilevel geographic information. We assembled five microbiome datasets from the global urban environment collected during 2016 and 2017 (#1 and #2, respectively) (Danko et al. 2021), the urban environment of three highly sampled cities: Hong Kong, New York City, and London (#3) (Danko et al. 2021), the soil environment (#4) (Delgado-Baquerizo et al. 2018a), and the marine environment (#5) (Sunagawa et al. 2015) d). i) We then applied the mGPS model g) to the RSA data of the different environments h). The training:testing of the aforementioned datasets was done as follows: 1 + 2:1 + 2, 2:1, 3:3, 4:4, and 5:5.

The importance of predicting microbial communities cannot be overstated. Beyond forensic capabilities (Elhaik et al. 2021), developing a biogeographical model for metagenomes has multiple ramifications. It can be used to study the fundamental processes that determine biodiversity and compare them quantitatively among microbial groups, which can shed light on the relationships between community dissimilarity and environmental parameters (De Gruyter et al. 2020). Understanding the geographic distributions of microorganisms promotes a better understanding of our surroundings, as there is a growing appreciation for the environment–microbiome–health axis (Ahrens et al. 2024). When humans engage in international travel, migration, and forced displacement, their mobility is assumed to be connected to the colonization and transmission of multidrug-resistant organisms, which is linked to the international spread of antimicrobial resistance (AMR) genes (MacPherson et al. 2009), one of the biggest challenges facing modern medicine (Afshinnekoo et al. 2021). For example, assume that AMR bacteria are transferred from the Levant into Mexico via illegally traded species through an airport. While the specific animal may have already been sold and cannot be identified, the microbiome signature of the merchants can be traced to Southern Indonesia, providing clues on the origin of the AMR bacteria (Fig. 1a). Therefore, inferring the dynamics and biogeography of human movement, pathogens, microbes, and AMR transmission will aid in the development of policies related to human activities and AMR, as well as travel guidance recommendations, to reduce the risk of acquiring AMR microorganisms. Similarly, the global trade of goods like foods is a source of the AMR spread (Hassan and Kassem 2020). Tracing AMR transmission routes is essential to assess the potential of epidemiological risk and population migration to AMR transmission.

Inferring the biogeographical origins of microorganisms remains a complex and unresolved challenge. To address this, we developed the microbiome geographic population structure (mGPS), the first machine learning (ML)–based tool that leverages microbial relative sequence abundances (RSAs) to pinpoint the fine-scale source locations of microorganisms. mGPS was rigorously tested on microbiomes from built urban, soil, and marine environments, sequenced using different approaches. mGPS's ability to distinguish local from nonlocal microorganisms allows it to effectively trace the global spread of AMR genes recorded in the MetaSUB dataset in 2016 and 2017.

## Results

### The mGPS Tool Implementation

Developing a biogeographical toolkit requires close coordination of experimental data collection (Shamarina et al. 2017) and method development with designing predictive models (Widder et al. 2016). For that, we implemented mGPS as a parameter-free ML-based tool that predicts the geographic source of microbiome samples from the RSA and metadata (Fig. 1). We postulated that predictive biogeographical models should capitalize on the spatiotemporal patchiness that some taxa exhibit. We termed these as geographically informative taxa (GITs) that can be used as markers for fine-scale biogeography. To select an optimal subset of bacterial taxa for predictive modeling, we applied a recursive feature elimination procedure (see Materials and Methods) that ranks taxa by how geographically informative they are and retains the most informative ones. mGPS was then trained on the RSA of the selected GITs, calculated from the fraction of reads classified to each taxon. The mGPS model was designed to produce predictions for different levels of regional locations (e.g. continent, country, latitude, and longitude). It is initially trained on the first hierarchical level for continent predictions. Then, the predictions are augmented with the training data and used to train the next model level, for which predictions would be augmented again with the training data until the final level is trained for specific coordinate predictions. Finally, mGPS outputs the predicted latitude and longitude.

To evaluate the performance of mGPS, we applied it to the RSA data calculated separately from the next-generation data of the urban biome (Danko et al. 2021) (global metagenomic dataset and three-city high-resolution dataset), the 16S rRNA gene-based soil biome (Delgado-Baquerizo et al. 2018), and the prokaryotic shotgun sequencing data of the marine biome (Sunagawa et al. 2015). These microbiome datasets used different sequencing platforms, data processing methods, and different read thresholds in calculating RSA, allowing us to rigorously test mGPS and its independence of data sequencing and processing.

### Biogeographical Predictions of Worldwide Transit Stations

We first analyzed the MetaSUB dataset (Danko et al. 2021), which contains the RSAs of 3,660 species identified from 4,135 metagenomic samples collected in 53 cities over 3 years (2015 to 2017) (Table 1; supplementary text S1, Supplementary Material online) (Ryon et al. 2022). The global RSA consisted of ∼97% bacteria, 0.97% eukaryotes, 0.01% viruses, 0.05% archaea, and ∼1.5% unannotated organisms. Interestingly, unlike typical microbial distribution patterns (Pascoal, Costa, and Magalhães 2020), where few microorganisms are common across sites and the vast majority is rare, here, the trend is disrupted by the existence of a large number of microorganisms in a large number of samples (supplementary fig. S1.1a1, Supplementary Material online). Overall, 55% of the species were common, and 45% were rare (appearing in less than 5% of the samples). The most common species, found in 95% of the samples, accounted for 1% of all species. Species fluctuated across cities, with all cities sharing 8% of the species and city-unique taxa accounting for 11% of all species, without a clear trend (supplementary fig. S1.1a2, Supplementary Material online). Across cities, 310 taxa were potentially pathogenic, 222 and 73 affecting animals (including humans) or plants, respectively, and 15 affecting both kingdoms. Globally, the most common potential pathogens were *Pseudomonas aeruginosa*, *Stenotrophomonas maltophilia*, and *Pseudomonas fluorescens*, all potentially pathogenic to humans. The average RSAs of potential pathogens varied by site. After Barcelona, English cities had the highest potential pathogen abundance based on our analyses (supplementary fig. S1.7a, Supplementary Material online). Pathogens are important biomarkers for mGPS predictions. Of the 200 MetaSUB GITs, 25 (12.5%) were potential pathogens.

**Table 1:** Sizes and counts of samples and taxa included in the basic and post-QC datasets

Dataset	Sample sizes and counts (basic QC)				Sample sizes and counts (post-QC)			
	Sample size	Number of cities, countries, or water bodies	Taxa count	Pathogens count	Samples size	Number of cities, countries, or water bodies	GIT count	Pathogenic GIT count
MetaSUB	4,135	53	3,660	310	4,070	40	200	25
Soil	237	18	25,224	7	231	13	200	0
Marine	131	9	2,328	50	131	9	200	1

The basic QC steps included removing unreliable samples, whereas follow-up steps included removing cities with small sample sizes. All the QC steps are detailed in the Materials and Methods section. Post-QC data were used to construct the mGPS model.

**Fig. 2.**
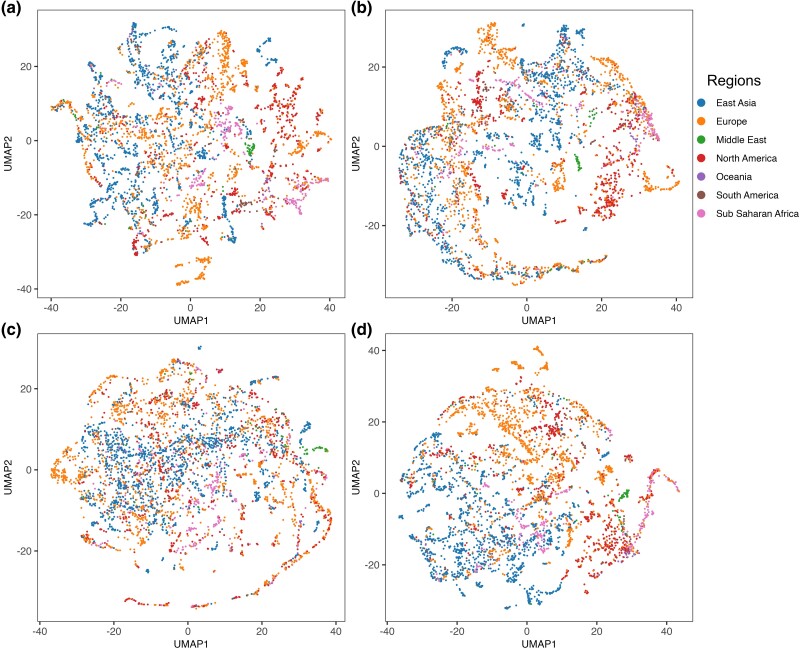
Global distribution of species. UMAPs of taxonomic proﬁles based on Manhattan distance between samples, which are color coded by the region of origin for each sample. Axes are arbitrary and without meaningful scale. a) All species, b) all non-GITs, c) 200 randomly selected non-GITs, and d) 200 GITs.

While the core urban microbiome is present in almost all samples, there was nonetheless a wide range of variation in taxonomy and localization across all the cities, already observed by Danko et al. (2021). To evaluate the suitability of the dataset for biogeographical applications, we carried out a dimensionality reduction of the RSA data using UMAP (uniform manifold approximation and projection) (McInnes et al. 2018) with Manhattan distance. UMAP (Fig. 2a) suggests that the entire microbiome is unsuitable for biogeographical applications due to the high overlap of microbiome from different regions. Compared with two controls consisting of all non-GITs (Fig. 2b) and randomly selected 200 non-GITs (Fig. 2c), using only 200 GITs species provided the most refined biogeographical predictions (Fig. 2d).

**Fig. 3.**
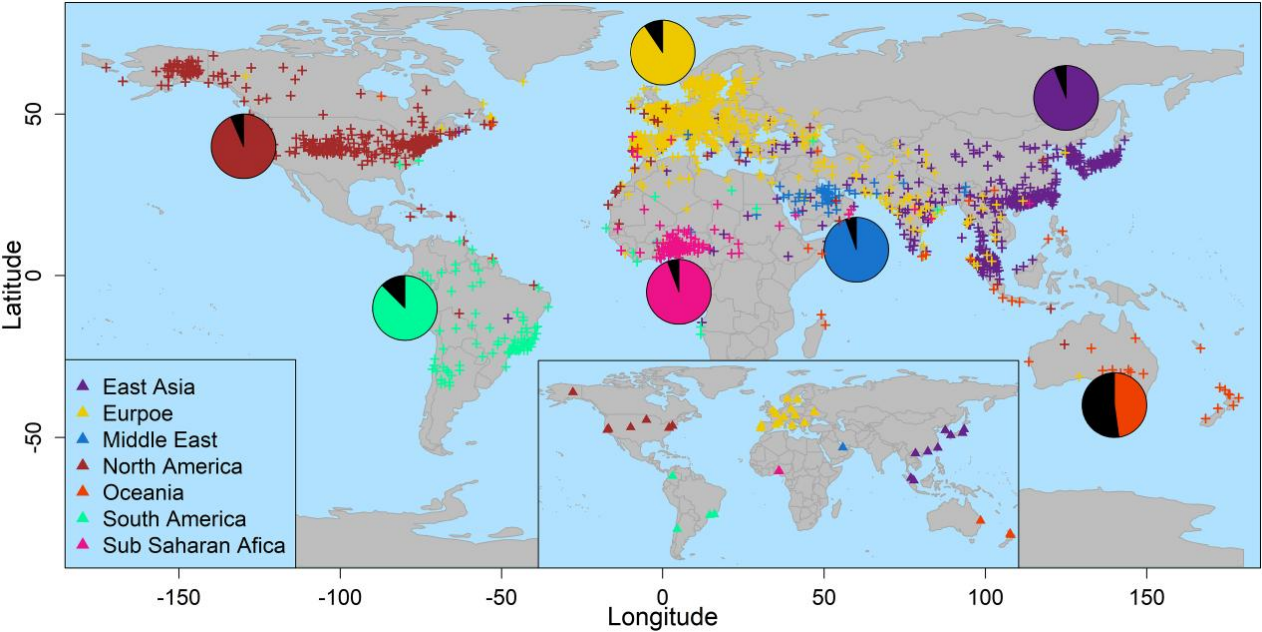
mGPS geographical predictions for the MetaSUB's global dataset. Crosses represent the predicted locations of MetaSUB samples. The pie charts show the proportion of samples that mGPS predicted for their sampling regions. Predictions are color coded based on their sampling region (triangle, inset); each includes several cities (see supplementary fig. S1, Supplementary Material online).

After quality control (see in Materials and Methods), we applied mGPS to 4,070 worldwide samples from 40 cities spanning all continents excluding Antarctica using 200 GITs. These GITs are globally abundant taxa with high regional variation, ensuring they would be found in all samples. mGPS predicted the source city and the geographical coordinates for each sample assigning 92% of the samples to their sampling city with high mean sensitivity (78%) (true positives over true positives and false negatives) and specificity (99%) (true negatives over true negatives and false positives) (supplementary table S1a, Supplementary Material online). Applying mGPS to the global microbiome dataset yielded high accuracy at regional (Fig. 3), city, and within-city levels (supplementary fig. S1, Supplementary Material online). mGPS predicted 62%, 74%, and 84% of the samples within 250, 500, and 1,000 km, respectively, from their sampling sites with a median distance of 137 km (Table 2; supplementary fig. S2, Supplementary Material online). We compared those results with those of a random model by reshuffling the RSAs, normalizing them to maintain the sum of taxa RSA as 1, and applying the trained mGPS model to these data. mGPS predicted only 0.05%, 0.2%, and 1.4% of the samples within 250, 500, and 1,000 km, respectively, from their sampling sites with a median distance of 7,445 km. We note that most microbiomes are expected to be predicted to their sampling site; however, deviations from this are also common.

**Table 2:** mGPS prediction accuracy per dataset

Dataset	mGPS prediction accuracy			
	% sample (0 to 250 km)	% sample (0 to 500 km)	% sample (0 to 1000 km)	Median distance (km)
MetaSUB	62%	74%	84%	137 from the sampling site
Soil	66%	71%	77%	0.74 from the sampling country
Marine	0	2.3%	10%	2,834 from the sampling site

For the MetaSUB and marine samples, the percent of predicted samples is reported in terms of distances between the sampling site and the predicted site. For the soil microbiome (all samples), the percent of predicted samples is reported in terms of distances from the sampling country (the finest geographical resolution for this dataset), with samples that match the sampling country and are considered to have a distance of zero.

**Fig. 4.**
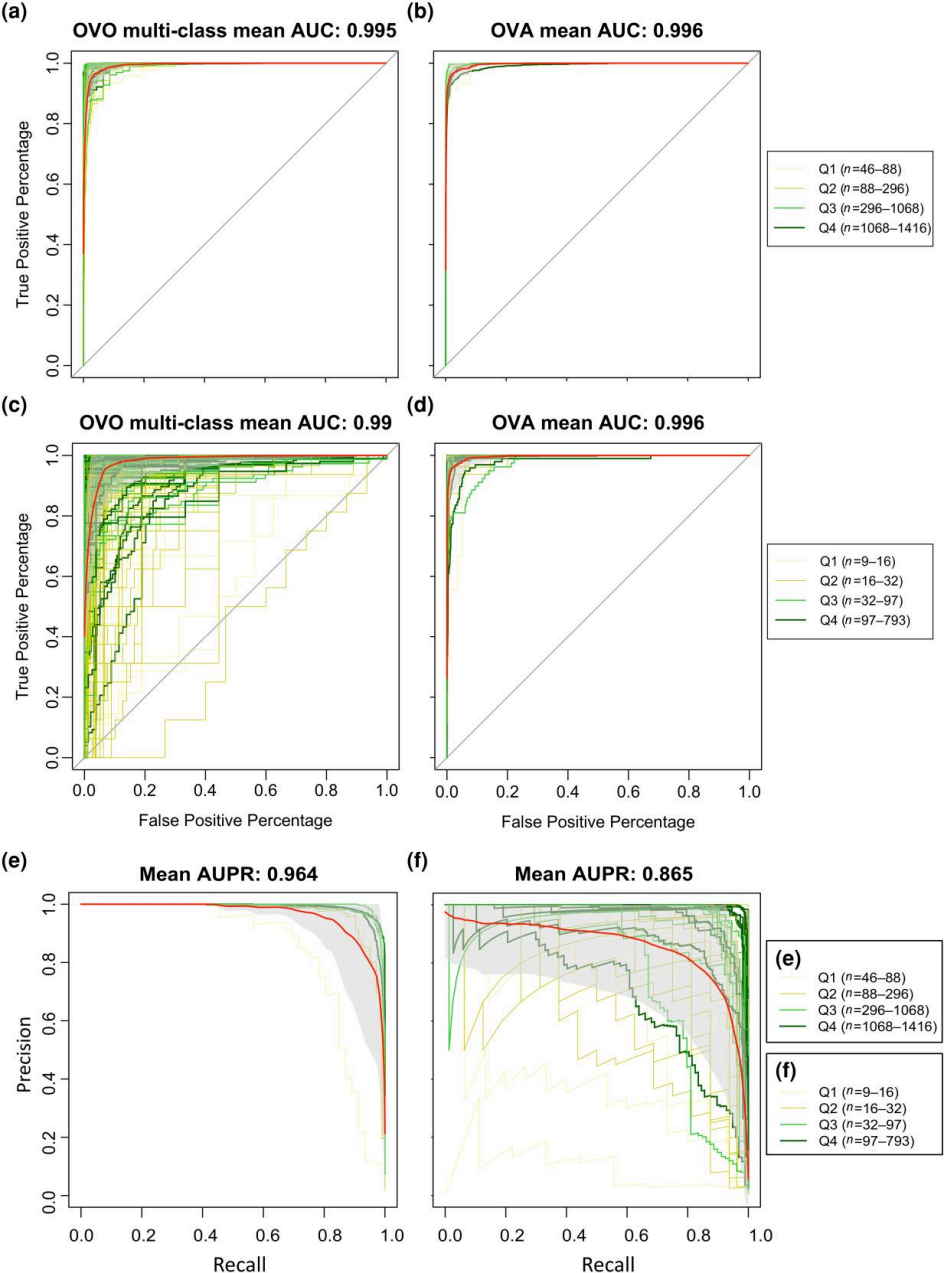
ROC and PR curves for mGPS classification of MetaSUB samples to continents (ROC: a and b, PR: e) and cities (ROC: c and d, PR: f). The OVO approach a and c) compares all possible combinations of geographical classes. The OVA approach b and d) compares each class against all the others. Each set of ROC and PR curves is color coded based on the quartile sample size (*n*) of continents or cities (see legend). The red line represents the mean ROC or PR curve. The gray background indicates one standard deviation of the mean. A horizontal line represents an AUC of 0.5 for reference.

To thoroughly evaluate the effectiveness of mGPS and confirm our findings, we created receiver operating characteristic (ROC) curves. These curves visualize the performance of mGPS's classification hierarchy (country and city). By examining these ROC curves that plot sensitivity against 1-specificity across various threshold settings, we can evaluate how well mGPS accurately classifies microbial samples from different geographical regions using different sample sizes. We employed both the one-vs.-one (OVO, comparing all possible two-class combinations [e.g. Asia vs. Europe; Hong Kong vs. London]) and one-vs.-all (OVA, comparing each class against all others) approaches to calculate multiclass results. The mean area under the curve (AUC) values ranged from 0.99 to 0.996 for both continent and city levels, regardless of the approach—OVO or OVA. This robust range of AUC values highlights the high-level performance of mGPS in discriminating between various geographical regions (Fig. 4a to d). Overall, the compelling AUC values reinforce the reliability and effectiveness of the mGPS approach in accurately predicting continents and cities.

Due to uneven sample distribution across locations, leading to dataset imbalance, we conducted additional analysis to gage mGPS's predictive performance in categorizing samples into continents (Fig. 4e) and cities (Fig. 4f) by carrying out a precision–recall curves (PRC) analysis. The AUCs of 0.97 and 0.87, respectively, underscore the accuracy and reliability of mGPS in effectively handling both broad geographical classifications and finer-grained city-level distinctions.

We found no correlation between the prediction accuracy and city population size (*T-test*, *n* = 40, *r* = 0.017, *P* = 0.92, 95% confidence interval [*CI*] = [−0.33, 0.30]) (supplementary fig. S3, Supplementary Material online). The highest concordance between mGPS predictions and the sampling cities was for cities that are well represented in the training dataset (*T-test*, *n* = 40, *r* = 0.53, *P* = 0.0005, 95% *CI* = [0.26, 0.72]) with the highest concordance for cities that are better represented in the training data (e.g. New York, Hong Kong, London) (supplementary fig. S4, Supplementary Material online). The exceptions were Singaporean samples that are poorly predicted although widely sampled, which may represent a high-traffic and heterogeneous microbial exchange network, and samples from the two Scandinavian cities, Stockholm and Oslo, which are accurately predicted despite being moderately represented in the training data, suggesting a distinct microbial signature in the area with respect to the remaining samples. For comparison, we retrained the model using a random subset of 200 taxa, excluding the 200 GITs. City prediction accuracy dropped to 77%, and only 44% of samples were predicted within 500 km of their sampling sites. These results indicate a substantial variation in the relationship between geography and taxa abundance. The differences in median abundance between the optimal GITs (supplementary fig. S5, Supplementary Material online) and randomly selected taxa (supplementary fig. S6, Supplementary Material online) show that only the first exhibit regional or city-specific geographic clustering.

To test the prediction accuracy of microbiome samples more rigorously, we next applied a leave-one-out procedure at the spatial cluster level. In this test, one region containing nearby cities is predicted after its removal from the training set (see Materials and Methods). The outcome is the mean prediction accuracy for all removed cities. Due to the relative density of the MetaSUB data, mGPS's accuracy decreased slightly, with 36%, 51%, and 66% of samples predicted within 250, 500, and 1,000 km of their sampling site, respectively. As before, the prediction accuracy was not correlated with the population size (*T-test*, *n* = 40, *r* = 0.027, *P* = 0.87, 95% *CI* = [−0.287, 0.336]) but rather with the training data size (supplementary fig. S7, Supplementary Material online).

To determine the effect of heavily sampled regions on the accuracy, we retrained the model only on the 31 cities that had less than 100 samples and calculated a prediction accuracy of 87% with 95% CI (0.85,0.9), a slight drop from the 92% accuracy of the complete dataset. The mean sensitivity and specificity were slightly higher than in the complete dataset (80%, ∼100%, respectively) (supplementary table S1b, Supplementary Material online), suggesting that the existence of oversampled sites does not reduce the prediction accuracy.

Finally, to determine the robustness of mGPS to small sample sizes and potential temporal effects on the underlying microbiome profile, we separated the MetaSUB dataset into its two temporal subsets: the samples collected as part of Global City Sampling Day 2016 (gCSD16) and 2017 (gCSD17). We obtained 264 and 452 samples from cities sampled on both sampling days (Denver, Ilorin, Doha, New York, and Tokyo) in gCSD16 and gCSD17, respectively. Based on their sample sizes, we extracted GITs and trained mGPS on the gCSD17 subset, which had more samples, to localize the gCSD16 samples. The model predicted the sampling city for 62% of the gCSD16 samples with a mean sensitivity of 61% across all cities, similar to the result obtained from the combined datasets (Table 2).

These findings call for reevaluating the effects of the rank abundance curves (RACs) on the distance decay of microbiome community similarity. It has been suggested that the number of taxa shared among all samples will decrease with the number of studied samples because microorganisms exhibit a patchy spatiotemporal distribution in which few microorganisms are common while the vast majority are rare. Interestingly, we found that, on average, there were more taxa present in 60% to 80% of the samples compared to those found in 40% to 60% and 20% to 40% of the samples, most likely because of the role of humans in dispersing the urban microbiome and modulating the community dynamics (supplementary text S1, Supplementary Material online).

### Fine-Scale Biogeography at the Transit Station Level

To assess the fine-scale accuracy of mGPS, we next trained it on the quality-controlled MetaSUB data from the three most extensively sampled cities: Hong Kong (*n* = 664), New York (*n* = 105), and London (*n* = 542) (supplementary fig. S8, Supplementary Material online). In Hong Kong, mGPS predicted the subway station of origin for 82% of the samples with a mean of 53% sensitivity and 99% specificity. Moreover, nearly half of the samples were predicted within 1 km of their sampling site, with a median distance of 1.25 km.

**Fig. 5.**
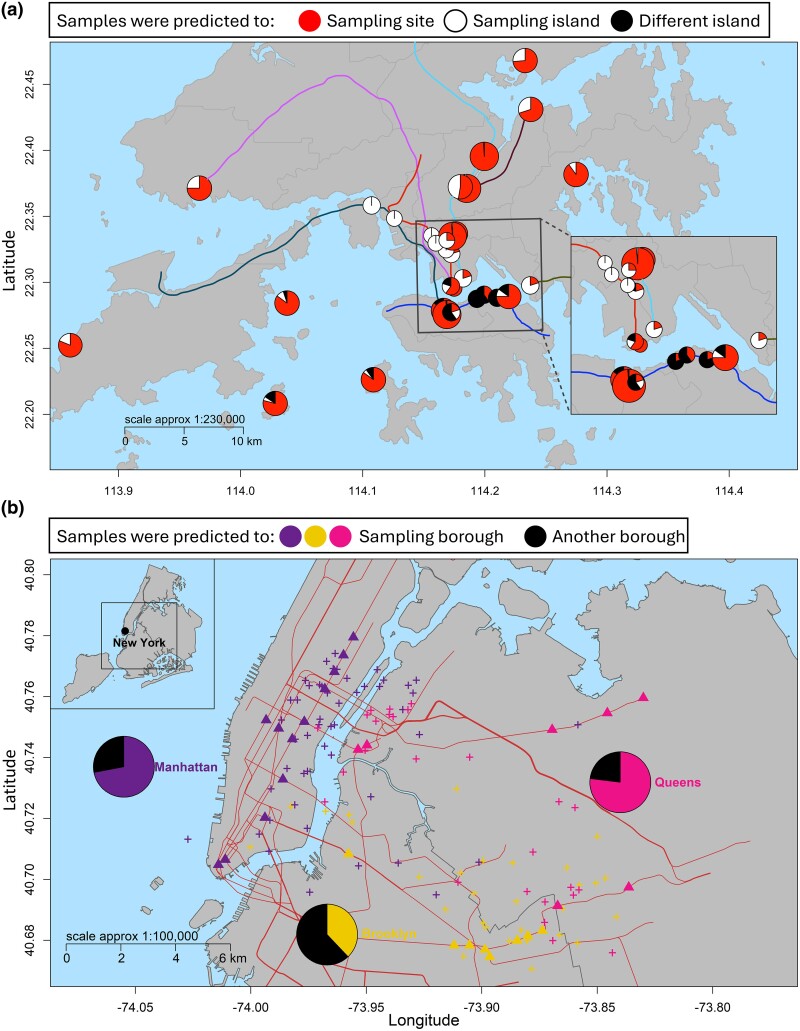
mGPS predictions for the transit systems and residential in Hong Kong a) and New York b) using the MetaSUB data. a) Pie charts depict the 33 sampling sites in Hong Kong and the prediction accuracy. All major train lines are shown. Sampling was done along these train lines. Pie chart sizes represent the number of samples taken at the corresponding sample site: large pie chart, 69 to 99 samples; medium pie chart, 16 to 21 samples; and small pie chart, 4 to 6 samples. b) Triangles show the New York sampling sites, and crosses show the predicted locations, color coded by the three boroughs. Pie charts depict the prediction accuracy. Symbols may overlap.

Hong Kong is divided into small islands by the ocean. We separated Hong Kong into distinct regions based on the three main islands in the north, west, and south. We observed clear separation and spatial clustering of predictions around stations (supplementary fig. S9, Supplementary Material online), even between stations in high proximity, as 86% of the sample coordinates were predicted to the sampling island region (Fig. 5a). Our model explained a large amount of the variance in sample coordinates (*r^2^* = 85% and *r^2^* = 63% for latitude and longitude, respectively). By contrast, in a random model with reshuffled RSAs, mGPS predicted only 9.7% and 28.4% of the samples to their original subway station and sampling island region, respectively. No samples were predicted within 1 km of their sampling site, with a median distance of 21.4 km. The high concordance between mGPS predictions and the sampling sites at such a fine-scale level indicates the remarkable distinctiveness of the microbial communities of each transit station. mGPS's classification accuracy in Hong Kong was further evaluated by utilizing the OVO (mean AUC of 0.92) and OVA (mean AUC of 0.97) methodologies (supplementary fig. S10a and b, Supplementary Material online). The PRC plot demonstrated how undersampling resulted in a reduced accuracy of mGPS. The high AUPR (∼0.6) can be expected to be improved if sufficient data are provided (supplementary fig. S11a, Supplementary Material online).

In the New York MetaSUB subset, mGPS predicted the transit station of origin for 43% of the samples, likely due to the smaller sample size (an average of 3.6 samples per station, compared to 20.1 per station for Hong Kong). Consequently, less information was available to train the model during each iteration of the cross-validation procedure. Nonetheless, the predicted coordinates showed a high clustering level toward the sampling boroughs, allowing us to predict 64% of the samples within the right borough (Fig. 5b) with a median distance of 2.39 km from the transit station. Most of the remaining samples were predicted along the borough boundaries. After reshuffling the RSAs of samples, mGPS predicted only 24% of the samples within the right borough with a median distance of 6.56 km from the station. mGPS's classification accuracy in New York was also evaluated by utilizing the OVO and OVA methodologies, both yielding a mean AUC of 0.87 (supplementary fig. S10c and d, Supplementary Material online). The AUPR (>0.4) can also be expected to improve further if sufficient data are provided (supplementary fig. S11b, Supplementary Material online).

Due to the layout of London's subway stations, most sampling took place at stations in the city center. As a result, while the London MetaSUB dataset is comparable in sample size to the Hong Kong dataset and nearly five times larger than the New York City dataset, it exhibited the lowest geographical spread and smallest average sample size (87% of London stations had fewer than four samples, whereas over 50% of Hong Kong stations had at least 15 samples available). While there was a high concordance between the sampling sites and the city in the global dataset (supplementary fig. S1c, Supplementary Material online), these limitations have considerably reduced the fine-mapping efficiency of mGPS. To overcome these data limitations, we divided the city into six clusters using *k*-means clustering (*K* = 6) applied to sample coordinates. mGPS predicted the sampling region for 48% of samples (supplementary fig. S12a, Supplementary Material online). After reshuffling the RSAs of samples, mGPS predicted the sampling region for only 20% of the samples.

The distribution of samples to clusters was uneven, ranging from 44 to 191 in the central cluster raising questions as to how sample distribution affects mGPS's accuracy. To test how uneven cluster sizes, affect mGPS's performances, we carried out several analyses where we manipulated the cluster sizes (Table 3). First, we randomly sampled 20 and 40 samples from each cluster 100 times and calculated the median cluster prediction accuracy as 30.8% and 35.8%, respectively (supplementary fig. S12b, Supplementary Material online). Compared with the original dataset, the low accuracy of the subsets showed that even sample sizes with undersampling were not advantageous for mGPS. Next, we tested three more conformations using even sample sizes in five clusters, with the last cluster enriched as much as possible. These conformations included even sampling with enrichment of the South London (R1) cluster, which yielded an accuracy of 35.7%, with 155 samples clustering at the R1 cluster but not the central R5 cluster, and even clustering with enrichment of the Central London (R5) cluster, which yielded an accuracy of 46%, with 139 samples clustering at the largest R5 cluster. Finally, we tested a similar conformation to the last one with even higher enrichment of the R5 cluster, which yielded an accuracy of 52.22% with 245 samples clustering at the fifth and largest cluster (supplementary fig. S12b, Supplementary Material online). Overall, these results show that mGPS is unbiased toward the central clusters but, as expected, is affected by the sample sizes, with the largest samples offering more training opportunities. Evaluating mGPS's classification accuracy in London using the OVO and OVA methodologies yielded mean AUCs of 0.75 and 0.77, respectively (supplementary fig. S10e and f, Supplementary Material online). The relatively low AUPR (0.42) indicates the low precision of mGPS in London (supplementary fig. S11c, Supplementary Material online).

**Table 3:** Testing mGPS performances for different cluster sizes in London

		Test	Cluster sizes of London samples						mGPS cluster prediction accuracy	Note
			South London (R1)	North London (R2)	East London (R3)	Northeast London (R4)	Central London (R5)	West London (R6)		
Experiment	1	Original dataset	102	77	44	52	191	76	47.8%	…
	2	Small and even sample size (*n* = 20)	20	20	20	20	20	20	30.8%	Median accuracy for 100 models
	3	Medium and even sample size (*n* = 40)	40	40	40	40	40	40	35.8%	Median accuracy for 100 models
	4	Medium and even sample size with a medium outlier (R1, *n* = 100)	100	40	40	40	40	40	35.7%	…
	5	Medium and even sample size with a medium outlier (R5, *n* = 100)	40	40	40	40	100	40	46.0%	…
	6	Medium and even sample size with a large outlier (R5, *n* = 160)	40	40	40	40	160	40	52.2%	…

The number of samples for each of the six clusters in London (see supplementary fig. S12a, Supplementary Material online) was manipulated to evaluate the accuracy of mGPS.

Overall, the global and fine-scale trends highlight the robustness of the mGPS across diverse datasets and population sizes, alongside its sensitivity to insufficient sampling data, suggesting that mGPS performances can be expected to improve over time as more data become available. While London was predicted accurately in the MetaSUB city dataset, mGPS could not achieve fine-grade predictions as those achieved for Hong Kong and New York City.

### Biogeographical Predictions for Soil Microbiomes

We analyzed the RSA data from a worldwide survey of soil microorganisms across 18 countries on six continents that detected 25,224 bacterial phylotypes (common soil bacterial species) in 237 samples (Delgado-Baquerizo et al. 2018) (Table 1; supplementary text S1, Supplementary Material online). In over 95% of the samples, just 0.03% of the phylotypes were shared, and fewer than 1% were distributed across all countries (supplementary fig. S1.1c, Supplementary Material online). Considering the dominant soil taxa, all phyla except WS2 were present in all samples (supplementary fig. S1.3c2, Supplementary Material online). The most common phylotypes were affiliated with *Bradyrhizobium*, *Rhodoplanes*, and *Mycobacterium*—all Pseudomonadota. The RSAs of common and rare phylotypes differed by country. Unlike in the MetaSUB dataset, where common taxa had a high RSA, common soil phylotypes did not. Puerto Rico had the highest abundance of rare phylotypes, followed by Peru (supplementary fig. S1.2c1, Supplementary Material online). The most common potential pathogen was the plant pathogen *Pseudomonas viridiflava*, followed by the animal and human pathogen *Stenotrophomonas maltophilia*. Potential soil pathogens were relatively uniformly distributed across continents (supplementary fig. S1.7c, Supplementary Material online).

**Fig. 6.**
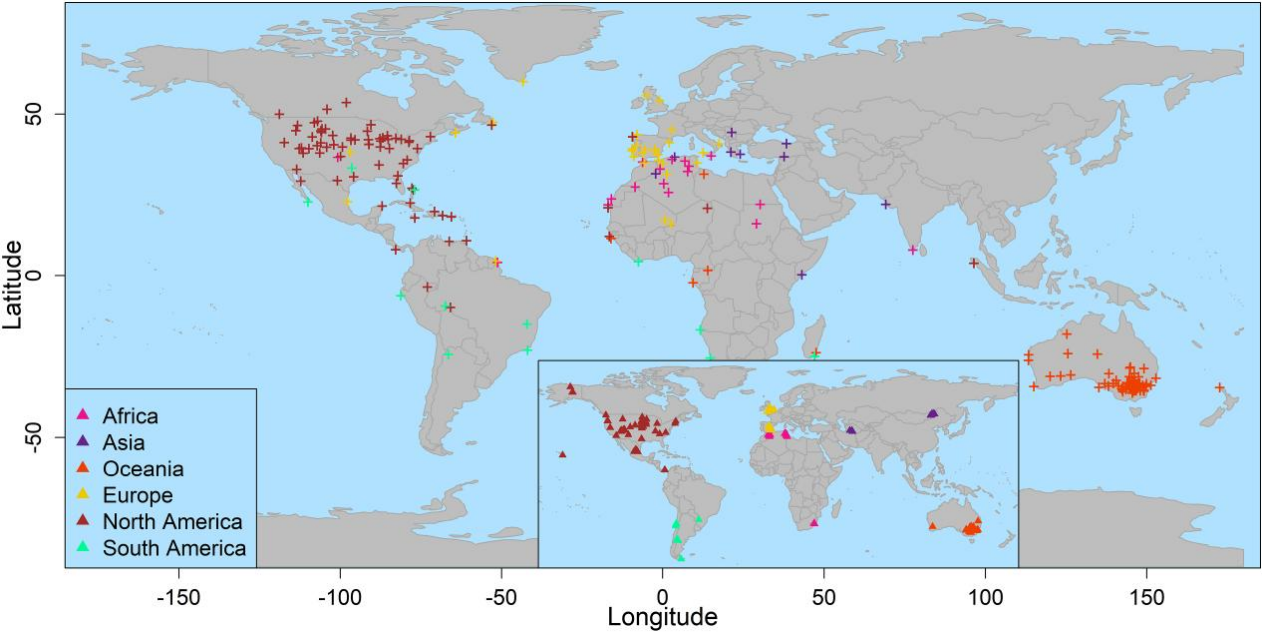
mGPS's geographical predictions for the soil microbiome data. Crosses represent the predicted location of the 231 samples in the dataset. Predictions are color coded based on their sampling region. Sampling sites are marked in triangles (inset).

We applied mGPS to the quality-controlled soil microbiome data (231 samples collected from 13 countries). The geographic dispersion of the top GITs demonstrates the potential of mGPS for biogeographical predictions for this dataset (supplementary fig. S13, Supplementary Material online). For example, the genus *Actinomycetospora* (operational taxonomic unit [OTU], #2106) of Actinobacteria (most likely *A. chiangmaiensis*), first isolated from tropical rainforest soil in Northern Thailand, was one of the taxa found primarily or solely in Australia. Although each sample contained over 500 abundant taxa, only a subset of 200 taxa provided the highest prediction accuracy, suggesting that most soil taxa are, at best, weakly associated with geography (supplementary fig. S14, Supplementary Material online). To address inconsistent sampling in the soil microbiome data, we employed the synthetic minority oversampling technique (SMOTE), which equalizes sample sizes by oversampling. After oversampling to ensure sample sizes of not less than 10 for all countries, mGPS predicted the sampling country for 88% of the samples with a mean of 75% sensitivity and 99% specificity. After reassigning samples to countries (the finest geographical resolution available in this dataset) based on the predicted latitude and longitude, samples that match the sampling country were considered to have a distance of zero from the sampling country. For samples not assigned to the sampling country, the distance between the predicted coordinates and the sampling country was calculated. Looking at the predicted geographical coordinates (Fig. 6), 61% of samples were predicted within 100 km of their true sampling countries and 71% within 500 km (Table 2). Expectedly, countries well represented in the training dataset were predicted more accurately (supplementary fig. S15, Supplementary Material online). Comparing those results with those of a random model by reshuffling RSAs, mGPS predicted the sampling country for only 28% of the samples, with 2.2% and 2.6% of samples predicted within 100 and 500 km, respectively.

Here, too, we applied a leave-one-out procedure, but at the country level because, unlike in the MetaSUB dataset, the soil dataset is sparsely sampled. mGPS's prediction concordance with the sampling site decreased, with 33% of predictions made within 100 km of their country of origin and 41% within 500 km. These results illustrate one of the known limitations of ML tools: their requirement of relatively large datasets for training. When provided with a medium-sized, sparsely sampled dataset, ML algorithms cannot be adequately trained, which results in lower accuracy (Fig. 6). Overall, the results reinforce both the regional uniqueness of some microbial communities and the relationships between geography and biodiversity, which allow the identification of the geographical source of microbial samples with high accuracy based solely on microbial RSA data. Lastly, we generated ROC curves to visualize the performance of the classification hierarchy of mGPS models in predicting samples to continents and countries utilizing the OVO and OVA approaches. The average AUC ranged from 0.94 to 0.97, demonstrating the robustness of mGPS performances for soil microbiomes (supplementary fig. S16, Supplementary Material online). The excellent AUPR (>0.8) demonstrates highly accurate localization, even in an imbalanced dataset. As before, smaller sample sizes were associated with lower prediction accuracy (supplementary fig. S17, Supplementary Material online).

### Biogeographical Predictions for Marine Microbiomes

We last analyzed the Tara Oceans microbiome dataset, which includes 2,328 taxa and 131 samples from nine oceanic water bodies (Sunagawa et al. 2015) (Table 1; supplementary text S1, Supplementary Material online). Archaea accounted for about 5% of taxa, whereas bacteria comprised the remainder. Here, a large percentage of taxa (22%) appeared in all nine oceanic regions, and 6% appeared in only one ocean (supplementary fig. S1.1b2, Supplementary Material online). The most common phyla were Actinobacteria, Bacteroidetes, and Cyanobacteria. A small number of potential pathogens were found, primarily affecting animals.

**Fig. 7.**
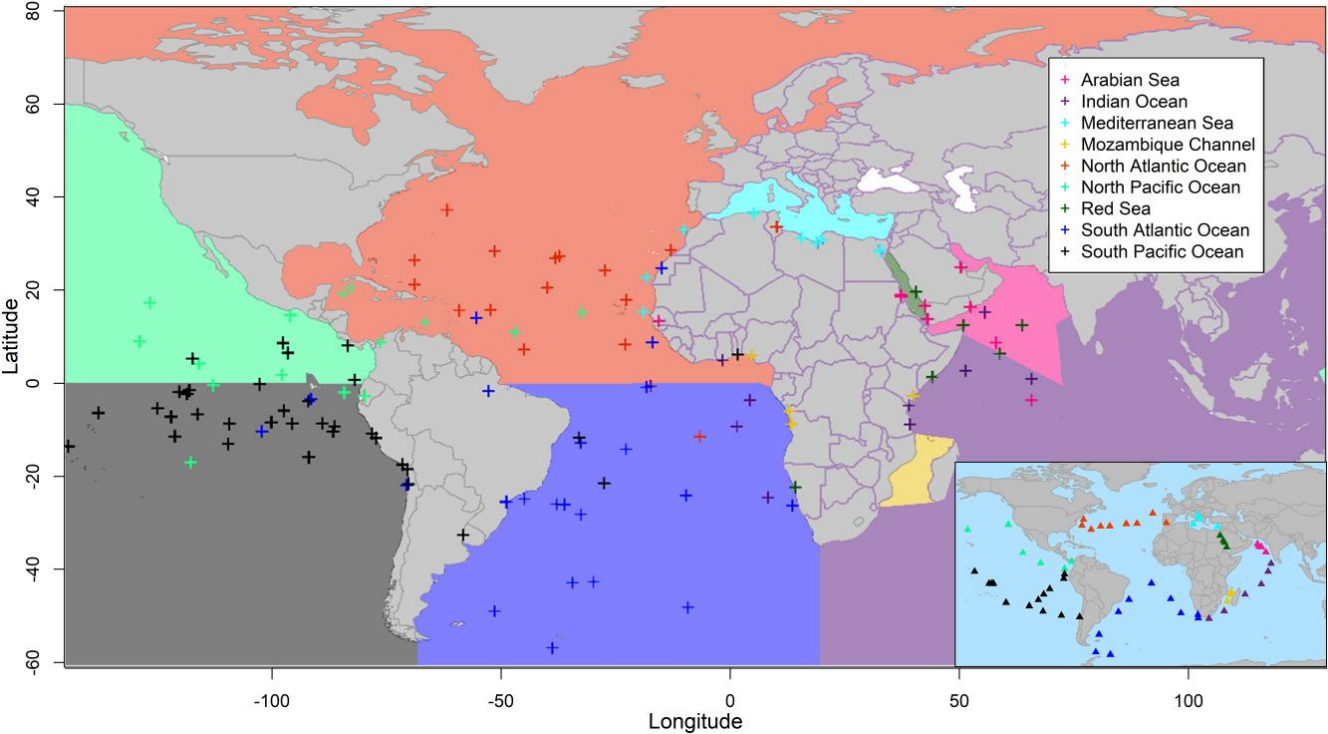
mGPS's geographical predictions for marine microbiome data. Crosses represent the predicted coordinates for all marine microbiome samples (*n* = 131) color coded by the oceanic body of origin. Sampling sites are marked in triangles (inset).

mGPS achieved 74% accuracy in predicting the oceanic water body of origin with a mean sensitivity of 70% (Fig. 7; supplementary table S2, Supplementary Material online). The median distance of the predictions from their true origin is 2,834 km, which is not unexpected given the homogeneity of surface ocean habitats, the dispersion of samples, their mobility in the aquatic medium, and the small size of this dataset. When compared with the random model with reshuffled RSAs, mGPS only predicted the sampling oceanic region body for 7.6% of samples, with a median distance of the predictions from their true origin of 8,035 km (Table 2). Finally, we generated ROC curves to visualize the performance of mGPS in predicting samples to oceanic bodies. Using the OVO and OVA approaches, the average AUC ranged from 0.93 to 0.94, demonstrating the robustness of mGPS performances for marine microbiomes (supplementary fig. S18, Supplementary Material online). The very good AUPR (∼0.7) demonstrates highly accurate localization, even in an imbalanced dataset. Very small (Q1) and large (Q4) sample sizes were associated with lower and higher prediction, respectively, with intermediate sample sizes (Q2 to Q3) showing mixed patterns (supplementary fig. S19, Supplementary Material online).

### Mobility among mGPS Predicted Local and Nonlocal Taxa

In the remaining study, we focused on the MetaSUB dataset due to its large size and high biodiversity. Further analysis of the 200 GITs found in the MetaSUB dataset showed that the most informative GITs are not rare in specific cities but instead have a higher dispersion in samples and cities (occupancy value). GITs account for most of the taxa's RSAs in cities, with the most dominant phyla being Pseudomonadota, Actinobacteria, and Firmicutes. Gammaproteobacteria, Actinomycetia, and Alphaproteobacteria were the most abundant classes. Interestingly, 12.5% of the GITs were global potential pathogens that varied in dispersal between cities. We found no correlations between the pathogens’ RSAs and the cities’ populations, nor between the pathogens’ alpha diversities and the cities’ populations (Table 1; supplementary text S2, Supplementary Material online).

**Fig. 8.**
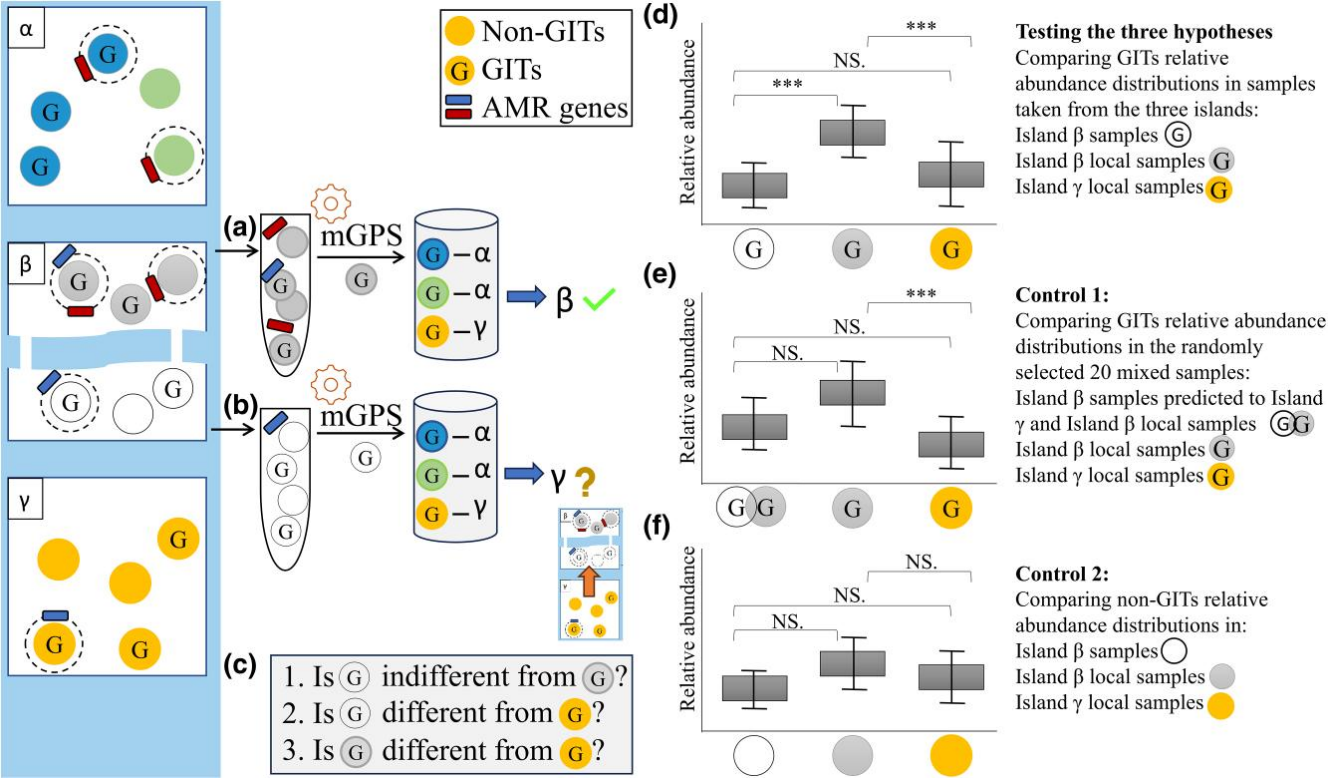
A statistical framework for testing mGPS's off-site predictions using differences in the RSA distributions of GITs. Islands α, β, and γ harbor diverse microbiome populations, which include GITs (“G”) and non-GITs (empty circles), some of which carry AMR genes (rectangles). In a), mGPS correctly predicted test samples (gray) from the Northern island β to β, although not previously sampled. In b), mGPS predicted test samples (white) from the Southern island β to γ. c) Testing the validity of these predictions requires answering three questions. For that, we compared the RSA distribution of the GITs in the test sample (nonlocals, immigrants) to local samples from sites β and γ (predicted as such by mGPS) in d). If the test sample's RSA distribution is significantly different from that of the neighboring sites, which is significantly different from that of the predicted site, and if the sample's RSA distribution is similar to that of the predicted site, it supports mGPS's prediction that the test sample originated in the predicted site. Two control cases are next described: in e), mixing the test (nonlocally predicted) samples with the neighboring site and selecting 20 random samples. If the mixed samples’ distribution is similar to both distributions of the neighboring sites and the predicted site and if the distribution of the neighboring sites is significantly different from that of the predicted site, it supports mGPS's prediction that the mixed sample arrived from sites β and γ) In f), the original analysis is repeated for non-GITs. Finding no difference between the three distributions demonstrates that non-GIT taxa carry little to no biogeographical information compared to GITs. Significance was assessed using the Wilcoxon signed-rank test with the *P*-value marked in the plots as 0 to 0.001*** or NS (nonsignificant difference).

Establishing the accuracy of mGPS predictions, whether on or off the sampling site, can be challenging given that we have information only on the sampling site and the site of origin (where the sample originated before it was translocated to the sampling site) is unknown. In our former analyses, we demonstrated that mGPS could predict most samples to their sampling sites and that, unlike classification algorithms, it can do so even if it was not trained on the sampling site. By analogy, consider the three-island model shown in Fig. 8a. Trained only on samples collected from islands α and γ, mGPS predicted samples collected from the northern area of island β. It did that by comparing the relative abundances of the β GITs to those of α and γ GITs in a similar manner to what we described above. However, mGPS predicted the samples collected from the southern area of island β to island γ (Fig. 8b), suggesting one or more translocation or migration events from islands γ to β. Are these predictions wrong?

Thus far, we treated off-sampling-site mGPS predictions as incorrect predictions, as we reported accuracy under the assumption that most predictions should match the sampling site. However, this assumption ignores the mobility of microbiomes due to migrations, import, tourism, and similar mechanisms that translocate microbiomes between environments. While we lack the historical knowledge to prove these translocations, we can support them using a statistical framework. To test whether mGPS off-sampling-site predictions are reasonable and whether mGPS can distinguish local from nonlocal microorganisms, we developed a test that compares the GITs’ RSA distributions to answer the three questions shown in Fig. 8c.

The rationale of our test is that if mGPS correctly predicts the source site, then the RSA distribution of the tested GITs should be more similar to those of samples in the predicted site than to those of the sampling site, given that the local populations of the two sites exhibit different RSAs. If the answer to all three questions is positive (Fig. 8c), we can confidently say that mGPS off-site predictions are correct. Overall, if mGPS correctly predicts samples as nonlocal, the GITs’ RSA distributions would exhibit a specific pattern shown in Fig. 8d. This pattern entails two notable distinctions (determined by the Wilcoxon test with a significance level of *P* < 0.05): firstly, between the nonlocal samples and the local samples at the sampling site, and secondly, between the local samples at the sampling site and the local samples at the predicted site. By contrast, there will be a nonsignificant (*P* > 0.05) or less significant difference between the samples predicted nonlocally and the local samples in the predicted site, as is the case in this example. We propose two controls, the first of which consists of mixed samples predicted locally and nonlocally, which violate the first condition (Fig. 8e). The second control consists of non-GITs violating the first and last conditions (Fig. 8f).

The aforementioned pattern (the test sample's RSA distribution is significantly different from that of the neighboring sites, which is significantly different from that of the predicted site, and the sample's RSA distribution is similar to that of the predicted site) shown in Fig. 8d is expected to be most visible in geographically remote prediction, and sampling sites are expected to harbor different microbial communities. To test if we can observe it in mGPS predictions, we selected three distinct geographical region groups and searched for the above pattern among the top 15 most important GITs (supplementary text S3, Supplementary Material online). We observed this pattern 8.7 times on average (supplementary fig. S3.1, Supplementary Material online), demonstrating that the nonlocally predicted samples have likely originated in the predicted sites. The pattern was not observed in the first control group, which consisted of mixed samples predicted nonlocally and locally (e.g. a mix of American samples predicted to America and Europe). As a second control, we analyzed non-GITs. The RSA distributions remained mostly unchanged between the three groups, and the pattern appeared only 2.6 times on average in the 15 randomly selected non-GITs. These results demonstrate that mGPS correctly predicts the source site in most cases and can be used to infer AMR transmission routes.

Comparing the proportion of MetaSUB samples predicted by mGPS to their local sampling region or elsewhere (e.g. do the migration), we found that the Middle East has the smallest nonlocal proportion and Oceania the highest, with more than half of all taxa being nonlocal (supplementary fig. S3.2, Supplementary Material online). On the city level, Barcelona had the smallest proportion of nonlocal taxa. We note that mGPS distinguished both the samples and individual local and nonlocal taxa.

Comparing the RACs of the local and nonlocal samples (supplementary text S3, Supplementary Material online), we found that, excepting Oceania, nonlocal samples tend to have a lower taxa richness. Oceania had the lowest taxa richness in the world. The variation in taxa uniformity of the local and nonlocal migrants provides a novel means of comparing urban microbiomes (supplementaryfig. S3.3, Supplementary Material online).

### AMR Genes of Local and Nonlocal Samples Differentiated by mGPS

As bacterial resistance and antimicrobial diversity rise, epidemiology increasingly focuses on the spread and transmission of AMR genes (Ahmad et al. 2021). Current approaches to identifying the source of AMR genes involve a combination of microbiological, molecular, and epidemiological techniques, requiring extensive data collection. A previous study has verified that AMR genes are urban-specific and can be used to predict the origin of urban microbial samples (Casimiro-Soriguer et al. 2019). However, until now, bioinformatic tools that can leverage this knowledge to support such efforts at low cost have remained unavailable.

mGPS provides a simple, accurate, and affordable means to localize AMR samples. By applying mGPS to the samples, we can predict their source, and because AMR genes have been discovered in each sample, we can differentiate local and nonlocal samples to trace AMR transmission. For example, assume that island α bacteria had only the red AMR and island γ only the blue AMR (Fig. 8). mGPS can be used to show that a bacterium from northern island β with the two AMRs is the likely product of AMR gene transfer from southern island β, which migrated from island γ (Fig. 8b) and reached the southern island β via its land bridges, which can now be better monitored.

**Fig. 9.**
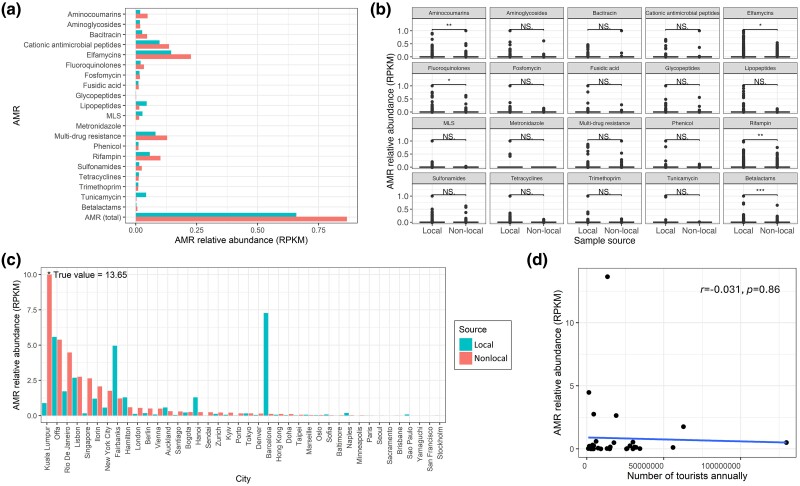
AMR relative abundance (RPKM values) patterns. a) Comparing RPKM values of each AMR between local and nonlocal (migrant) samples on a global level. Bar colors distinguish local from nonlocal samples. b) Comparisons of AMRs’ RPKM value (after min–max normalization) distributions of local and nonlocal samples globally. Significance was assessed using the Wilcoxon signed-rank test with the *P*-value marked in the plots as 0 to 0.001***, 0.001 to 0.01**, 0.01 to 0.05*, or NS (nonsignificant difference). c) Total AMR RPKM values for local and nonlocal samples per city. Colors as in a). d) The correlation of total AMR RPKM values of nonlocal samples and the annual number of tourists (supplementary table S3, Supplementary Material online) (*Pearson-test*, *n* = 35, *r* = −0.031, *P* = 0.86).

To study the influence of microorganism migration on AMR spread, we compared the relative abundance of 20 common AMRs using reads/kilobase/million mapped reads (RPKM) (Danko et al. 2021) in samples that mGPS predicted as local or nonlocal. We found that the RPKM values of distinct AMRs differ between the two groups, with the nonlocal samples having higher RPKM values overall (Fig. 9a). The two groups exhibited significant differences (Wilcoxon test: *P* < 0.05) in the RPKM values in aminocoumarins, elfamycins, fluoroquinolones, rifampin, and beta-lactams (Fig. 9b). The total AMR RPKM values varied by city. Nonlocal samples had higher AMR RPKM values in about 55% of cities. Among the top 15 cities with the highest AMR RPKM values, 75% had nonlocal samples with higher AMR RPKM values than local samples (Fig. 9c). Compared to locally predicted samples, nonlocal samples tend to have higher AMR relative abundance (Fig. 9a and c), demarking the transmission routes. Interestingly, we found no correlation between the AMR relative abundance of nonlocal samples and the city's tourist population annually (*Pearson test*, *n* = 35, *r* = −0.031, *P* = 0.86) (Fig. 9d).

**Fig. 10.**
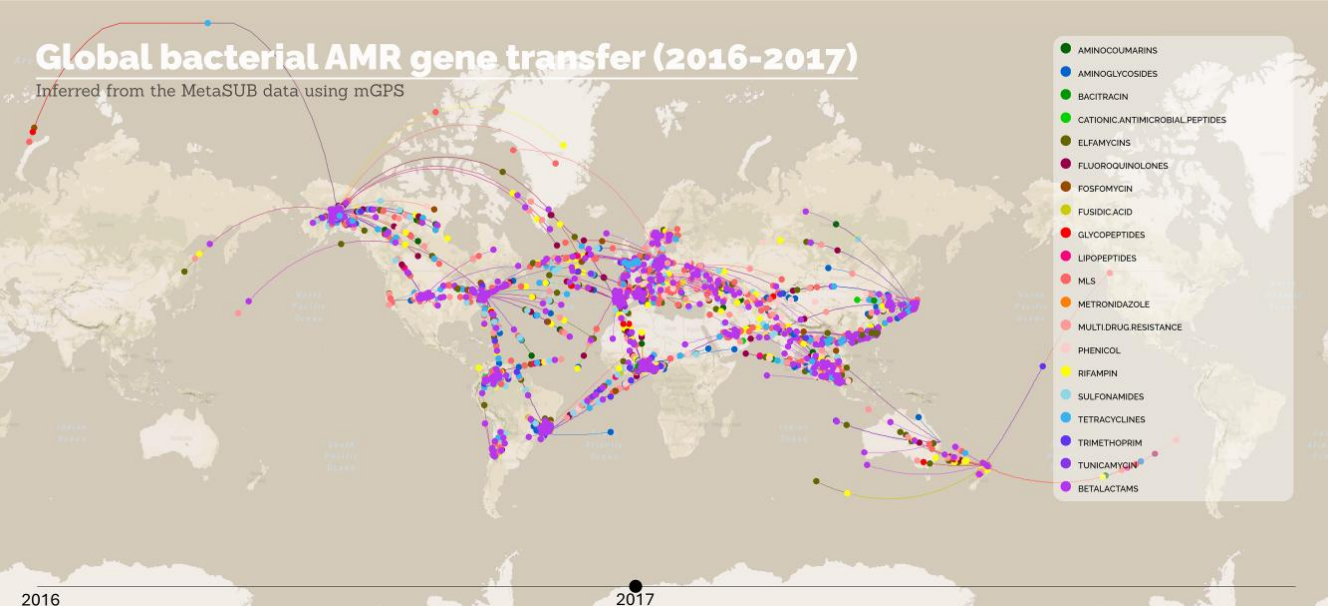
A snapshot of the AMR transmission at a global scale from the MetaSUB dataset. Samples move from mGPS predicted site of origins to their sampling site.

Finally, we sought to understand how AMRs are transmitted globally by charting the geographical start and end points of the 20 most common AMRs. mGPS was already applied to the AMR-containing samples, and it was predicted that they would be local or nonlocal (where the source site differs from the sampling sites). Considering mGPS's predicted source sites as the starting points and the sampling sites as the ending points, we could trace the migration route of each AMR. We used an animation to show how AMR transfers globally by plotting those 2016 and 2017 migrations when data were collected (supplementary videoS1, Supplementary Material online; Fig. 10). We found that in 2016, AMR was transmitted mainly from America, Western Europe, Eastern Africa, and Japan, whereas in 2017, the focus moved to Southeast and Eastern Asia and the whole of Europe (supplementary fig. S20, Supplementary Material online). We emphasize that AMR types vary by site. For example, the five most common AMRs (MLS, beta-lactams, elfamycins, rifampin) and multidrug resistance genes are globally transmitted (rifampin and multidrug resistance were not traced to Oceania). By contrast, tunicamycin originated in South and North America, West Africa, and West Europe and has spread to Northeast America and North Africa. Overall, our findings offer a longitudinal and latitudinal view of AMR transmission.

## Discussion

In recent years, multiple large-scale projects aimed at improving our knowledge of a global microbiome, with efforts ranging from ocean to soil to the built environment and even archeological sites (e.g. Sunagawa et al. 2015; Thompson et al. 2017; Delgado-Baquerizo et al. 2018; Habtom et al. 2019; Danko et al. 2021; Zhang et al. 2023). It is intriguing to ask to what extent the data generated by microbiome projects are geographically identifiable. Traditional community analyses revealed commonalities within and between communities (Leung et al. 2021) and that microbial abundance exhibits geographical differences (Wu et al. 2022). However, those studies were unable to biotrace microorganisms, nor could they distinguish local from nonlocal microorganisms, without which AMR transmissions cannot be tracked.

Past efforts to develop microbiome-based biotracing technology were limited by relatively small sample sizes, specific ecosystems, or specific sequencing technology and could not be applied universally (Mason-Buck et al. 2020; Robinson et al. 2021). In the absence of a biogeographical tool, studies resorted to classification instead of prediction. For instance, it was shown that the microbiome of nine offices across three cities showed a high degree of similarity between offices within the same city, allowing classifying samples to cities with 85% accuracy (Chase et al. 2016), that dust samples were classified to their national source with 90% accuracy based on the presence of fungi (Grantham et al. 2020), and that 88% of microbiome samples can be classified to cities (Danko et al. 2021).

This motivated us to develop the mGPS—the first ML-based tool that employs microbiome RSA data to predict their source communities. mGPS is a parameter-free tool that relies solely on the RSA of the most GITs over large geographical regions. mGPS is agnostic to the ecosystems’ ecological properties, different sequencing approaches, taxonomic definitions, and bioinformatics pipelines. However, in the case of multiple microbiome datasets that differ in those properties, mGPS may best be applied separately to each dataset, as done in this study, unless the datasets are produced using the same standards and pipelines. This is because microbiome research has experienced exponential growth over the past decade; however, studies still suffer from irreproducibility across investigations (Baykal et al. 2024), even when analyzing the same sample (Wood et al. 2021), due to using different pipelines and a lack of standardization. While there has been an increased emphasis on quality control and standardization in recent years (e.g. Amos et al. 2020; Meyer et al. 2023), these practices have not been adopted by all data-producing labs. These issues hinder any meta-analysis of different datasets. Briefly, mGPS first identifies GITs that exhibit distinct geographic distributions. Next, it is trained to associate their RSA patterns with geographical coordinates to predict their source location (Fig. 1). Interestingly, 12.5% of the MetaSUB GITs were potential pathogens like *P. aeruginosa* and *S. maltophilia*, both are globally widespread (Table 1). While it is reasonable to expect a large agreement between the sampling and predicted sites, as demonstrated in Figs. 3 and 5 to 7, predictions outside the sampling sites should be justified. For that, we developed a statistical framework that employed three statistical tests that together offer support for those predictions (Fig. 8). We have shown that mGPS is applicable to microbiome data from very different microbiome environments: urban, soil, and marine and trace samples from worldwide regions, countries, cities, broader areas within cities, and, in some cases, even to individual sites.

The high resolution of the MetaSUB dataset demonstrated the fine-scale resolution of mGPS in inferring biogeography. This was evident in Hong Kong, where mGPS could distinguish between two subway stations just 172 m apart. In New York City, mGPS could differentiate a kiosk from a nearby handrail that is less than a meter away. These results contrast with the low prediction accuracy in London, where only half of the samples were correctly assigned to their geographical clusters. We speculated that the high accuracy of prediction accuracy in Hong Kong is due to the cleaning protocols of their subway systems, described as “absolutely spotless,” which include regular cleaning, antibacterial coating to handrails and escalators, and implementing procedures to minimize contact between incoming and outgoing passengers (Maitin 2018), even before COVID-19. These practices “reset” the microbiome signatures from geographically diverse regions and reduce the mixing of microbiomes from different environments, which creates a unique microbiome profile for each station. By contrast, the London underground stations were described as “often dirty” and lacking sufficient handholds, resulting in passengers bumping into each other and mixing their microbiomes (Maitin 2018). Because cleaning practices improved post-COVID-19, repeating this analysis with newer samples would be intriguing.

It is well established that small sample sizes challenge ML tools and can reduce their accuracy. However, mGPS achieved high prediction accuracy for the soil and marine datasets despite their relatively small sample sizes. This success can be attributed to the high differentiation of the GITs in these biomes. Due to the vast differences between the three datasets, it is difficult to assess to what extent specific differences in protocol, sampling strategies, or sequencing techniques affected the accuracy of mGPS results. However, analyzing the within differences of each dataset showed that low sample sizes and uneven samples were the largest factors that reduced prediction accuracy.

Further analyzing the MetaSUB dataset, we found that migratory samples (i.e. predicted elsewhere from their sampling site) are typically richer in AMR genes. We also differentiated local and nonlocal taxa, which were predicted elsewhere than the sampling site, allowing tracking of the spread of AMR genes in 2016 and 2017. Although international travelers are proportionally much more likely to spread AMR (Hassan and Kassem 2020), we found that there was no correlation between the number of tourists in each region and the total RSA of the 20 common AMR genes for nonlocal samples, indicating that rising tourism may not be the main reason for the foreign AMR. Therefore, foreign AMR is considered more likely to be transmitted by drug-resistant bacteria brought about via alternative transmission routes like trade, animal transmission, and discharge of wastewater into the environment (Grenni et al. 2018; Kraemer et al. 2019). It is noteworthy that the timing of the tourism count does not exactly coincide with the AMR sample collection time. This can be further studied more accurately by collecting more temporal samples. For the antimicrobial compounds beta-lactams and fluoroquinolones (Rice 2012), which are highly susceptible to resistance, the relative abundance distribution of their resistance genes was shown to be significantly different between local and nonlocal samples, and the nonlocal samples exhibited higher RPKM values for both types of compounds. Combined with mGPS for their source prediction, it is expected to provide data and support for AMR biotracking, facilitate the construction of effective models for AMR transmission from a spatial scale, and provide more information for policy and environmental monitoring against antimicrobial treatments and outbreaks (Zhu et al. 2018).

Throughout this study, we trained and applied mGPS to each dataset separately to demonstrate the typical use of mGPS. An intriguing question is whether an all-environment generic mGPS model is feasible. While we cannot test it with the current datasets due to the nonoverlap of the taxa, we believe this is possible, provided the training data includes all environments of interest. This is supported by our observations that the GITs are environment specific. The existence of GITs in other environments in the training dataset will not affect a sample that completely lacks those GITs. Instead, the model will utilize the environment-specific GITs for that sample.

mGPS has several advantages. First, the input data (RSA data) required for the model training are easy to calculate and do not require additional annotation information such as environmental factors and functional microbial profiles (e.g. KEGG, resistance genes) (Casimiro-Soriguer et al. 2019). We do not deny that richer information would likely provide more accurate predictions, but this would defeat the applicability of mGPS to different datasets. Second, due to their small cell sizes and mostly single-celled lifestyle microbial communities have a higher diversity per area as plant and animal communities, hence they are prone to severe undersampling (Meyer et al. 2018), where rare species are not collected, and species abundance measures are not associated with the actual environment. mGPS reduces this risk by employing GITs, most of which have a higher dispersion in samples and cities (higher occupancy). Third, unlike current tools that use regional names to classify sample sources (Casimiro-Soriguer et al. 2019; Walker and Datta 2019), the final output of the mGPS regressor chain is latitude and longitude that can be directly tagged to the actual location in the map without the constraints of the class name (e.g. the classification labels need to be covered in the training set), thus achieving prediction of unsampled sites.

mGPS has several limitations. First, as with all ML methods, a relatively large training dataset (several hundred samples), globally uniform and balanced as much as possible, is required to yield accurate predictions. We can expect the prediction accuracy to improve over time as more data are accumulated. Second, imbalanced datasets may pose a challenge for mGPS. To overcome this challenge, we adopted three strategies: we removed cities with small sample sizes that insufficiently represented the microbial uniqueness of those cities; we trained the model using the XGBoost method, which is more robust for imbalanced datasets than alternative methods; and we demonstrated (in the soil dataset) that a simple resampling scheme can markedly improve the results. We also adopted a range of measures to evaluate the accuracy of mGPS, including AUC of ROC, F1 score, and AUPR. We acknowledge that further improvements can be made to reduce the impact of imbalanced datasets and will explore those in a separate study. Third, small, well-connected environments create a fertile ground for microbiome exchange and thereby reduce the accuracy of mGPS. For example, in the London metropolitan area, the major stations are sorted in a small and dense ring around London and branch into different lines; the closely knit stations homogenize the microbiome and prevent fine-scale geographical predictions, as in Hong Kong. Similar concerns exist for marine microbiome as it is unclear how localized it is. Fourth, temporal changes in microbiome communities may bias mGPS predictions. For example, in the MetaSUB dataset, all the sampling days were carried out around June 21 every year. We demonstrated via a temporal replication that mGPS (trained on the GSD2017 dataset) could accurately predict a novel dataset (GSD2016). Although the accuracy slightly dropped compared to the original model, the results show a promise that temporal effects are likely not a major barrier to the effectiveness of the mGPS algorithm. The effect of seasonality remains unknown for this dataset; however, both the soil and marine datasets were sampled yearlong. Here again, prediction accuracy is likely due to their small sample sizes rather than the effects of seasonality. We note that while soil bacterial communities exhibit distinct differences among geographical sites, they exhibit high similarities over the years (Liu et al. 2019). Much remains unknown about the mechanisms that govern compositional dissimilarity in biological communities over time and space, and large-scale studies are necessary to determine the seasonal effects on the microbiome. Currently, the existing contextual data are not comprehensive enough, but even assuming that the taxa abundance exhibits a large difference between the seasons if trained on seasonal data, mGPS could be modified to localize the microbiome and distinguish between seasons. Finally, the microbial community may change as the environment changes (Yang et al. 2020) (e.g. train lines added or removed, floodings). However, if the nature of the disturbance is short, the microbiome can be expected to revert to its predisturbed levels over time; otherwise, mGPS should be retrained for the new microbiome signature.

We envision that, with time, biogeographical applications will become enhanced for more worldwide microbiome communities due to the addition of GITs to the training dataset and potentially accounting for seasonality. Therefore, our results should be considered a lower bound to the full potential of mGPS for biogeography. Our study also provides additional insights into the relationships between bacteria and geography by showing that taxa exhibit varying degrees of relationship, and GITs should be preferred for biogeographical applications. mGPS is freely and publicly available and has a multifunctional, interactive, and user-friendly interface. We finally caution that with DNA sequencing now being routinely utilized at many locations, accumulating metagenomes, may make microbiome tracking a reality, which raises ethical, consent, ownership, legal, and privacy questions (Hawkins and O'Doherty 2011; Mason et al. 2014; Shamarina et al. 2017; Elhaik et al. 2021; Robinson et al. 2021).

## Materials and Methods

### Global Datasets

We analyzed three different microbiome datasets that differ in their ecosystem's ecological properties, different sequencing approaches, taxonomic definitions, and bioinformatics pipelines: the MetaSUB urban biome (*n* = 4,728) (Danko et al. 2021), with samples collected from urban mass transit systems in 60 worldwide cities during two global city sampling days (gCSD) in 2016 and 2017 available at https://github.com/MetaSUB/MetaSUB-metadata and https://portal.geoseeq.com/sample-groups/0625c15e-cf0d-4241-b33d-e967d55b91e62021. Samples’ taxonomic profiles were generated by KrakenUniq (v0.3.2) (Breitwieser et al. 2018), and the RSA, i.e. the fraction of DNA in a sample from a given taxon, was calculated after subsampling to 100,000 classified reads and dividing the number of reads of each taxon by the total number of reads in each sample (Danko et al. 2021). Unless specified otherwise, we combined the annual datasets to reduce data missingness. The name and coordinates of the city of origin were given for each sample. Seven cities with unclear geographical coordinates were removed, leaving 4,135 samples from 53 cities across 6 continents for the biogeographical analysis. The soil microbiome dataset (*n* = 237) (Delgado-Baquerizo et al. 2018) consisted of soil samples collected from sites across 18 countries across continents. Phylotypes were identified based on the 16S rRNA gene using the Ribosomal Database Project classifier (Cole 2005) and the Greengenes database (DeSantis et al. 2006) and then calculated RSAs after the samples were rarefied to 10,000 sequences (Delgado-Baquerizo et al. 2018). The marine dataset (*n* = 131) consisted of samples collected from nine global oceanic bodies during the Tara Ocean expedition (Sunagawa et al. 2015) (https://www.ebi.ac.uk/metagenomics/studies/ERP001736#overview). The taxonomy was profiled using 16S tags and metagenomic OTUs in the SILVA database (Quast et al. 2013). fetchMG (Sunagawa et al. 2013) is a tool for identifying microbial OTUs from metagenomic data based on single-copy phylogenetic marker genes. fetchMG-inferred profiles were employed to identify metagenomic OTUs (quasispecies). We analyzed the RSAs of the MetaSUB project provided by Danko et al. (2021). For the soil and marine dataset, the RSAs were calculated from the reads data by dividing the number of reads of each taxon by the rarefied 10,000 sequences (soil) and the total number of reads in each sample (marine) separately. All the datasets included the latitude and longitude coordinates of the sampling sites.

### Microorganism Distribution and Pathogenicity

UMAP plots were calculated using the R package umap (version 0.2.10.0) (McInnes, Healy, and Melville 2018). We calculated the regional average RSAs (a city in MetaSUB, a country in soil datasets, and an oceanic body in the marine dataset) and the global average RSA of microorganisms weighted by the sample size per region. We also calculated the occupancy by counting the number of times each microorganism appeared in the samples and regions. That is, sample occupancy and regional occupancy denote the percentage of samples or regions where a given taxon was present with RSA higher than zero, respectively. To calculate the occupancy of taxa at the phylum level, we annotated the taxonomic hierarchy of taxa using the Microbiome Directory V2.0 (Sierra et al. 2019) and GTDB (Parks et al. 2022). The heterotypic synonym of microorganisms was adjusted manually according to their name in the Microbe Directory, NCBI_taxonomy, and GTDB taxonomy in GTDB metadata. To calculate the sum of occupancy of each phylum, we clustered the taxa according to their phylum. To measure the microbial diversity, we sorted microorganisms in each dataset according to the rank of sample occupancy and found the most common taxa. Taxa that appeared in more than 95% of samples were considered *common*, whereas *rare* taxa appeared in less than 5% of samples. The distributions of common and rare taxa were compared using the RSA sum of each group per region. All the analyses were done in R (version 4.0.3) (R Core Team 2020). We calculated Pearson correlations using the function *stat_cor* in “ggpubr” package (version 0.4.0) (Kassambara 2020) and Wilcoxon signed-rank test using the function *geom_signif* in “ggsignif” package (version 0.6.2) (Ahlmann-Eltze and Patil 2021) and visualized the results using the “ggplot2” package (version 3.3.3).

Pathogenicity annotation was obtained from the Microbe Directory. Potential pathogens were divided into three groups based on their hosts: animal, plant, and dual-kingdom pathogens. The sample occupancy, regional occupancy, regional average RSA, and global average RSA were calculated as per above. For the alpha diversity indexes, we utilized functions from the “vegan” package (version 2.5-7) (Oksanen et al. 2013) to calculate various metrics. Specifically, we used the *estimateR* function to compute the observed species and the *diversity* function to determine the Evenness index, Shannon–Wiener diversity index, and Gini–Simpson diversity index. Because pathogenicity depends on various conditions (e.g. host susceptibility, environmental conditions, and interactions with other organisms), we considered the respective organisms’ potential pathogens.

### mGPS Implementation

#### QC Procedure

MetaSUB cities with insufficient sampling data (less than eight samples) were removed, leaving 4,070 samples from 40 cities with 3,669 taxa (including nine taxa recorded in the removed city samples that have a global RSA of zero otherwise). A second fine-scaled subdataset of the global MetaSUB dataset included three extensively sampled MetaSUB cities: Hong Kong, New York, and London. After filtering out samples with missing sampling coordinates and insufficient data, the dataset included 664 samples from 33 stations (Hong Kong), 105 samples from 29 stations (New York), and 542 samples clustered into 6 regions according the coordinates using *k*-means (kmeans() function in R) from 175 stations (London). Countries with insufficient sampling data (less than three samples per site) were removed from the soil dataset, leaving 231 samples from 13 countries with 511 taxa (Delgado-Baquerizo et al. 2018). Given the limited data size, we employed the SMOTE (Chawla et al. 2002) by function *SMOTE* in R package “smotefamily” (version 1.4.0) to oversample and ensure that sample sizes for all countries were not less than 10 (*k* = 3, *dup_size* was set to oversample countries to 10 samples). Due to their distinct geographic locations, Alaska and Hawaii were individually extracted from the USA during the oversampling process. The marine dataset had even sample sizes (5 ≤ *n* ≤ 34) and remained unchanged.

#### Feature Selection

Features are the variables that contribute to predicting the outcome. Selecting informative, discriminating, and independent features is crucial for mGPS to accurately predict geographical locations. In this context, the features represent the dominant taxa within microbial communities, which display varying abundance levels (RSA) across different geographical locations, thus providing geographic information. We termed those taxa GITs. To computationally identify the features (or GITs), we carried out recursive feature elimination using random forests and their well-established feature importance measures to remove redundant and nonpredictive variables. For that, the dataset is first randomly split into training (80%) and testing (20%) datasets. An initial random forest classifier is then trained for the classification of the relevant target variable—city for global MetaSUB data, station for local MetaSUB data subsets, country for soil data, and oceanic body for marine data—using the training data and all possible predictor variables (all taxa). The prediction accuracy is then calculated for the testing set. The accuracy of out-of-bag predictions for the random forest model is recorded. For each sample *i*, the prediction is made using the decision trees within the forest that did not use *i* for training, and the same is done after permuting each predictor variable. The difference between the two measures is averaged over all trees and normalized by the standard error. Based on this measure, the variables are ranked from the most to least important. Using this ranking, we retained five subsets of predictor variables $S_{i}$, representing the *i* most important variables. For MetaSUB, i=(50,100,200,300,500,1500); for the soil data, i=(20,30,50,100,200); and for the marine data, i=(100,200,300,500). For each subset *Si*, the model is retrained, and predictions are made for the held-back testing set. This process is repeated five times using different training and testing splits to reduce selection bias. The subset size that produces the highest average classification accuracy is considered the optimal size. Given this optimal subset size *i*, the most informative variables with the *i* highest average importance values are selected as the optimal predictor variables. For MetaSUB, a subset of 200 taxa was optimal (Table 1; supplementary fig. S21, Supplementary Material online). A separate feature selection process identified features for each of the three cities, where sampling was done at a fine-scale station level in New York (100), London (200), and Hong Kong (400). For the soil and marine data, 200 informative features (taxa) were found (Table 1). Variable importance plots are used to illustrate the importance of GITs in model prediction accuracy. These plots demonstrate the importance of each variable (GIT) by showing how much the model's accuracy decreases when each variable is excluded. The more significant the reduction in accuracy, the more critical the variable is for successful classification. The variables are presented in descending order of importance. The feature selection is built into mGPS, but users can adjust the feature size.

#### Model Training

To predict the geographical coordinates from the RSA data of the GITs, mGPS is trained to find a set of hyperparameters (GIT combinations) per set of hierarchical classes (continent, country, city, and station and the site's coordinates). The core mechanism of the mGPS model involves a chained series of gradient-boosted decision trees, i.e. a multilayered geographical model that employs independent tree-like models to predict the geographical coordinates (Fig. 1).

More specifically, consider the geographical hierarchy in the MetaSUB global dataset: *M*_1_ (continent), *M*_2_ (country, city, or site), *M*_3_ (latitude), and *M*_4_ (longitude). mGPS capitalizes on this hierarchical structure and utilizes correlations between latitude and longitude by employing a prediction chaining method, shown to improve prediction accuracy for multitarget problems (Melki et al. 2017). For our mGPS model (Fig. 1g), a vector *x* of the predictor variables (the RSA of the GITs) is used as input. For the first-level submodel *M*_1_, it predicts the first level of the geographical hierarchy. The predicted class probabilities from *M*_1_ are then augmented with the input vector *x*, which is passed as input for the next level submodel *M*_2_*—*the prediction of the next level in the geographical hierarchy. As before, the predicted class probabilities are augmented with the *M*_2_ input vector, which is passed as input for submodel *M*_3_, a regression model for latitude prediction. This prediction is augmented by the $M_{3}$ input vector passed as input to submodel *M*_4_, a regression model for longitude prediction. These submodels make up the mGPS prediction model. The predictions for submodels *M*_2_, *M*_3_, and *M*_4_, are given as the final model output $\left\{y_{1},\;y_{2},\;y_{3\;}\right\}$, respectively. *M*_2_ produces predictions at the country level for soil data, city level for MetaSUB global data, and transit level for MetaSUB's three local subsets. In the latter case, submodel *M*_1_ was omitted as there is no variation at a regional (continental) level. To train the mGPS model, 5-fold cross-validation is carried out at each level using only the training data; out-of-fold predictions are then passed to the next level submodel for training. The gradient boosted decision trees (GBDT) algorithm (Friedman 2001) builds on the concept of a decision tree. A decision tree uses a tree-like structure (or flowchart) in which each internal node is a test, and branches represent the test's outcome test, which leads to other nodes until the end nodes (or leaves) with the final outcome are reached. GBDT is based on iterations of nodes in a decision tree, randomly and additively generated based on a subset of the features found in the input data. Each node is built to improve the shortcomings of the previous node and produce the most appropriate separation between the features. All the underlying mGPS submodels employ GBDT with the XGBoost implementation (Chen and Guestrin 2016) designed to maximize speed and performance. XGBoost requires the careful tuning of a series of crucial hyperparameters. These include shrinkage, maximum depth of trees, the maximum number of iterations, minimum loss reduction for splitting, subsample ratio of columns, and subsample ratio of the training instances. These are optimized using the grid search method with 5-fold cross-validation during training to maximize prediction accuracy while not overfitting the training data. In this approach, 80% of the data were used to train the model, including hyperparameter tuning. The trained mGPS model was then used to generate predictions for the held-out 20% unseen dataset (supplementary fig. S22, Supplementary Material online). This was repeated five times using five disjointed testing datasets, resulting in out-of-fold predictions generated for all samples, which were used to assess mGPS's accuracy. All the results reported here, including AMR transfer patterns, were obtained from samples that were not used to train mGPS. All downstream analyses and predictions were done on the unseen dataset. We repeated the training procedure for each dataset.

#### Interface

To make mGPS more accessible, we developed a user-friendly interactive interface. mGPS can be used to train a new prediction model using our existing datasets, train a new prediction model using a new dataset, and finally predict the geographical source of samples provided by a prediction model. The interface provides various flags and plots.

### Evaluation of mGPS Performance

We used the term *sampling site* to denote the place from which samples were physically collected, the *predicted site* to denote mGPS's predicted location, and the *source site* to denote the original site from which the microbiome migrated to the sampling site (if different).

When analyzing MetaSUB global predictions, the *M*_2_ submodel was trained for city predictions. For the local predictions, in the case of Hong Kong, New York, and London, the *M*_1_ submodel was bypassed (as the continent of origin shows no variation at a local level), and the *M*_2_ submodel was trained for transit station or regional clusters, and coordinate predictions were generated using the same cross-validation procedure as for global predictions, including a separate feature elimination procedure carried out for each city. When applying the model to soil data, the *M*_1_ submodel was trained for continent prediction, the *M*_2_ submodel for country prediction, and *M*_3_ and *M*_4_ for latitude and longitude predictions, respectively. When applying the model to the marine data, the *M*_1_ submodel was trained for oceanic body prediction and *M*_2_ and *M*_3_ for latitude and longitude predictions, respectively. The accuracy of the classification hierarchy (M2) for mGPS models was reported for each model. To assess the accuracy of mGPS coordinate predictions, the distances between the sampling and predicted sites were calculated using the Haversine formula, which determines the great-circle distance between two points on a sphere, i.e., the shortest distance over the Earth’s surface. For the soil datasets, the distances were calculated from the predicted coordinates to the nearest sampling country border. Samples assigned to the sampling country based on predicted latitude and longitude were considered to have a distance of zero from that country.

To estimate mGPS assignment accuracy, we utilized the leave-one-out approach at the spatial cluster level. First, due to the high heterogeneity of the dataset, we divided each locality (country [relevant for the Soil microbiome] or city [relevant for the global MetaSUB microbiome]) into distinct spatial clusters. Clusters were obtained by applying *k*-means clustering (*K* = 120) to the haversine distances calculated from latitude and longitude coordinates. Due to their high dispersal, clusters were not calculated for the marine dataset. This generated more homogeneous sample clusters for each locality; each was held out when training the model. The predictions were made for this held-out cluster, and this was repeated for all clusters. We reported the sensitivity and specificity of the prediction model when it predicted the sampling site (although not a measure of accuracy), which were calculated for each locality and then averaged across all localities.

To further assess the accuracy of the mGPS model, we subjected our model to predict the origins of a shuffled microbial dataset. This evaluation determines the ability of mGPS to discern regional patterns in taxa's RSA compared to random predictions. For that, we randomized the RSA values of each taxon for each sample across all samples. We then applied the final model, trained on the original dataset, to predict the geographic origins of the shuffled samples.

ROC curves were used to evaluate the classification hierarchy of mGPS models and were calculated using the OVO and OVA approaches using the R package “pROC” (version 1.18.4) (Robin et al. 2011). OVO compares each pair of classes, considering one of the classes in a pair as the positive class and the other class as the negative class. Each pair has two ROCs because each class in the pair can be positive. OVA approach compares each class with all the rest, considering one class as the positive class and the others as the negative classes. After obtaining the ROC curves for all classes, we plotted the average ROC curve with one standard deviation. Here, we interpolated all ROC curves at 1,000 points using the *approx* function in the R package “stats” (version 4.2.2) (R Core Team 2020). Then, the mean and one standard deviation of all the true positive percentages for all classes were calculated for each false positive percentage point. The precision-recall curve (PR) and area under the curve (AUPR) were calculated using the R package “PRROC” (version 1.3.1) (Grau et al. 2015). For each class, we labeled it as a positive class, with others as negative class.

### Distinguishing Local from Nonlocal Samples and Comparing Their Biodiversity

Local samples were defined as samples that mGPS predicted to their sampling site (city-level), with nonlocal samples considered the outcome of migration when this is not the case. To calculate the RACs, we adopted an abundance cutoff of 10^−6^ RSA for each region using the R function *rankabundance* in the “Biodiversity” package (Kindt and Coe 2005). The Kolmogorov–Smirnov test, implemented in the R function *ks.test* in the “stats” package, was used to evaluate the difference in RSA distributions.

### AMR Transmissions

Using the AMR gene RPKM values dataset that describes the relative abundance of AMR (Danko et al. 2021), we compared the average RPKM values of each AMR gene in local and nonlocal samples globally and per city. To compare the RPKM distribution in local and nonlocal samples, we normalized the RPKM values of each AMR gene into a range of [0.1] by min–max normalization (Patro and Sahu 2015). Using the prediction and sampling sites as the first and second data points, respectively, we plotted the transmission of the nonlocal AMR genes during the two sampling years (2016 to 2017). The animation was produced using the professional GPS package licensed from Anath Genomic Consultants AB.

## Supplementary Material

evae209_Supplementary_Data

## Data Availability

mGPS is a freely available public tool with a friendly user interface developed with the shiny package in R (version 4.0.3). mGPS code, interface code, tutorial, and example files are publicly and freely available via GitHub (https://github.com/YaliZhang98/mGPS).
