# Supplementary material for: Microbiome Geographic Population Structure (mGPS) Detects Fine-Scale Geography
Source: Genome Biol Evol. 2024 Oct 7;16(11):evae209. doi: 10.1093/gbe/evae209 (PMC11557374; doi:10.1093/gbe/evae209)
Supplement: evae209_Supplementary_Data [file evae209_supplementary_data.zip › Zhang et al. 2023 - Supp.pdf]

Supplementary materials

The supplementary materials include:

Figures S1-S22

Tables S1-S3

Supplementary Text 1-3

A.

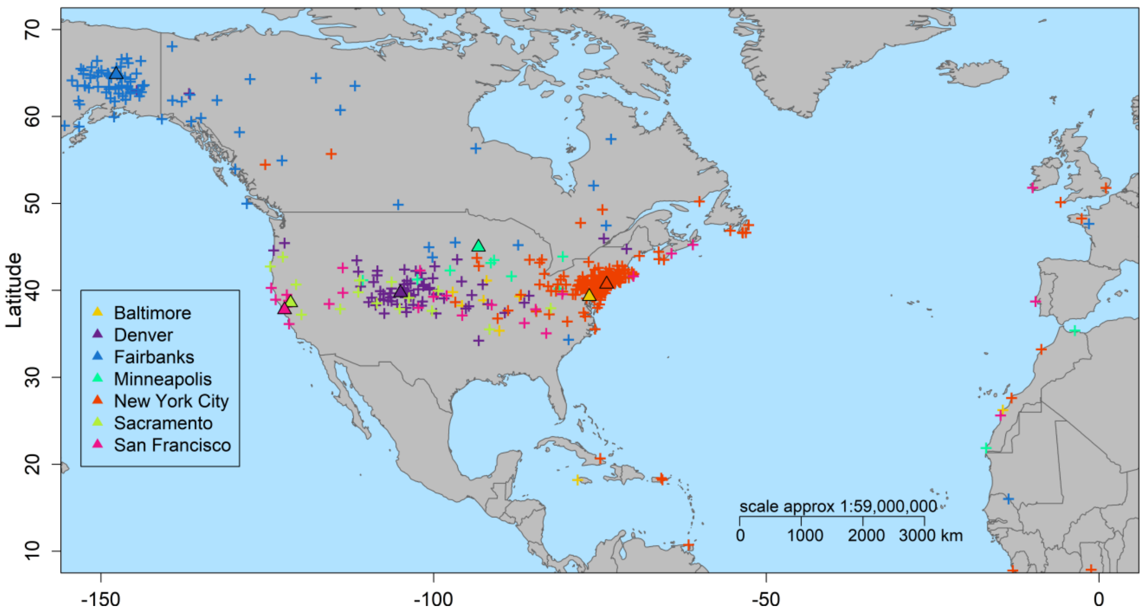

70
60
50
40
30
20
10
Latitude
-150
-100
-50
0
Baltimore
Denver
Fairbanks
Minneapolis
New York City
Sacramento
San Francisco
scale approx 1:59,000,000
0 1000 2000 3000 km

B.

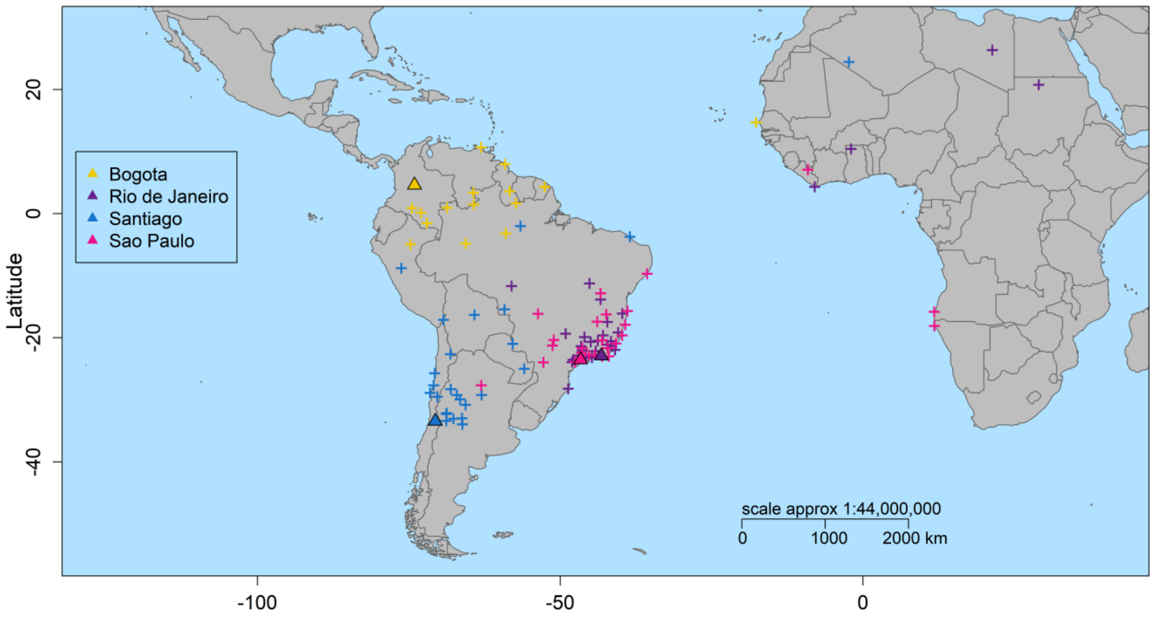

20
0
-20
-40
Latitude
-100
-50
0
Bogota
Rio de Janeiro
Santiago
Sao Paulo
scale approx 1:44,000,000
0 1000 2000 km

C.

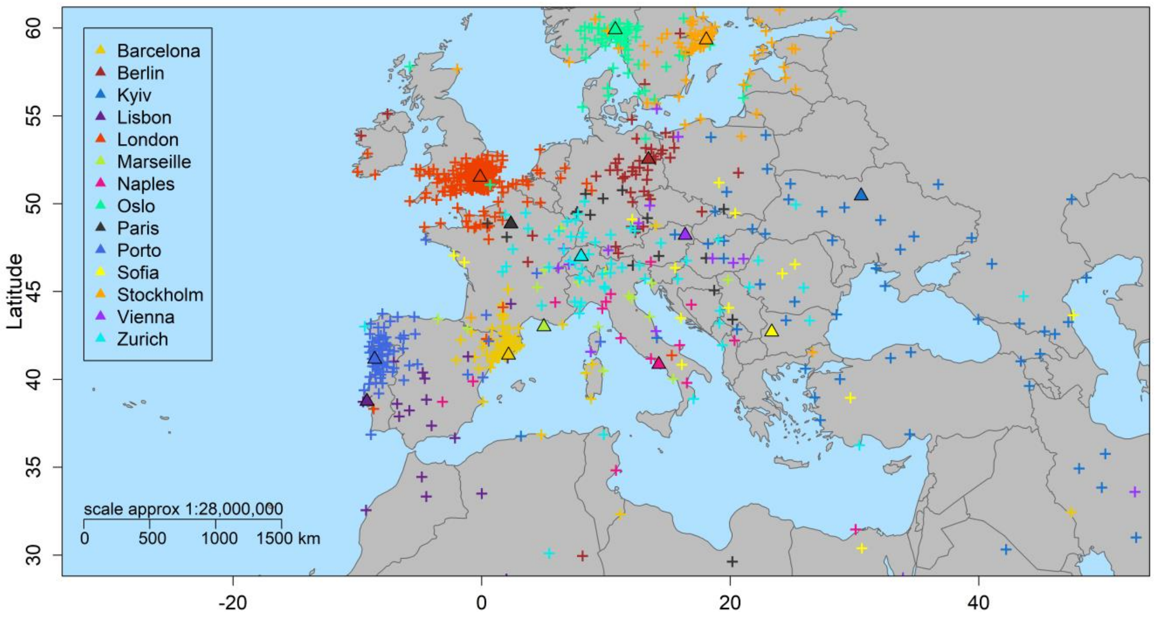
Barcelona
Berlin
Kyiv
Lisbon
London
Marseille
Naples
Oslo
Paris
Porto
Sofia
Stockholm
Vienna
Zurich
scale approx 1:28,000,000
0 500 1000 1500 km
Latitude
60
55
50
45
40
35
30
-20
0
20
40

D.

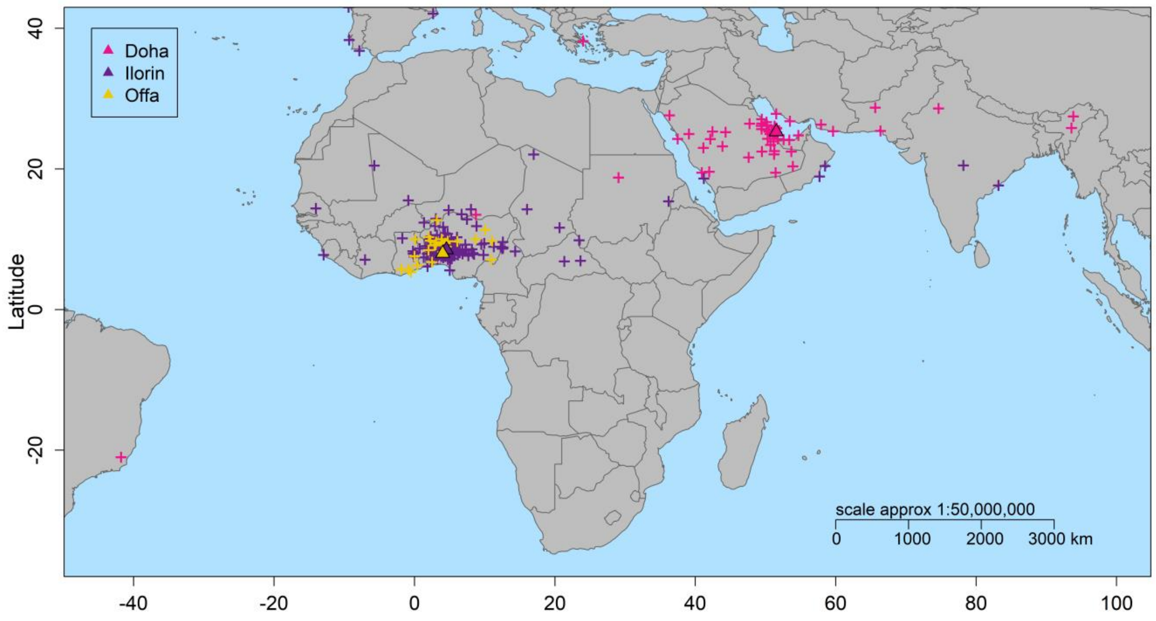
Doha
Ilorin
Offa
scale approx 1:50,000,000
0 1000 2000 3000 km
Latitude
40
20
0
-20
-40
-20
0
20
40
60
80
100

E.

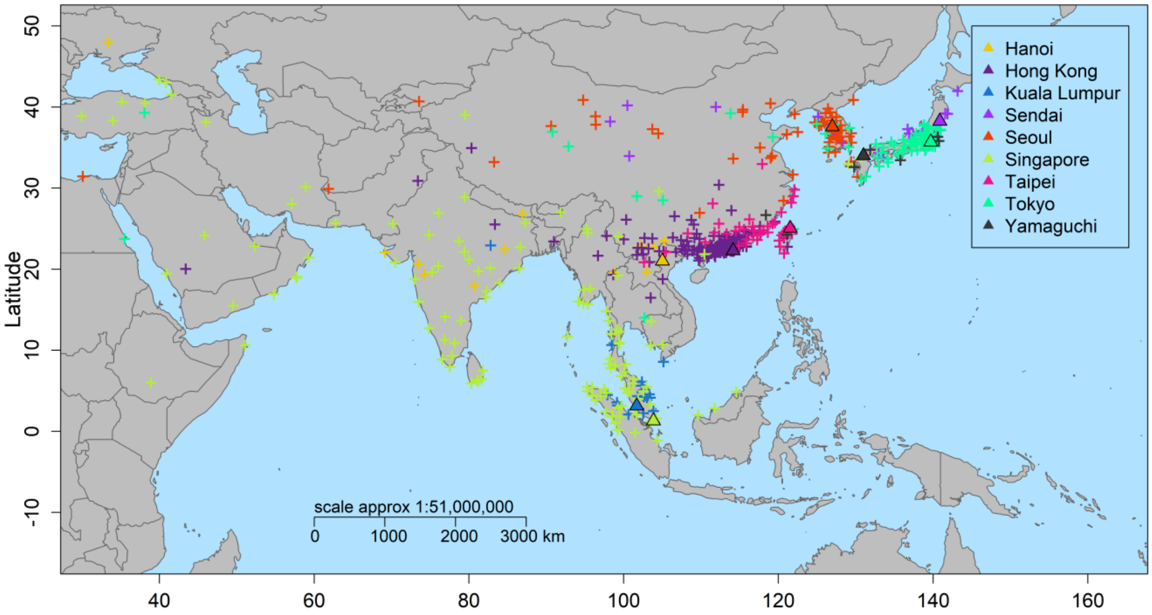

F.

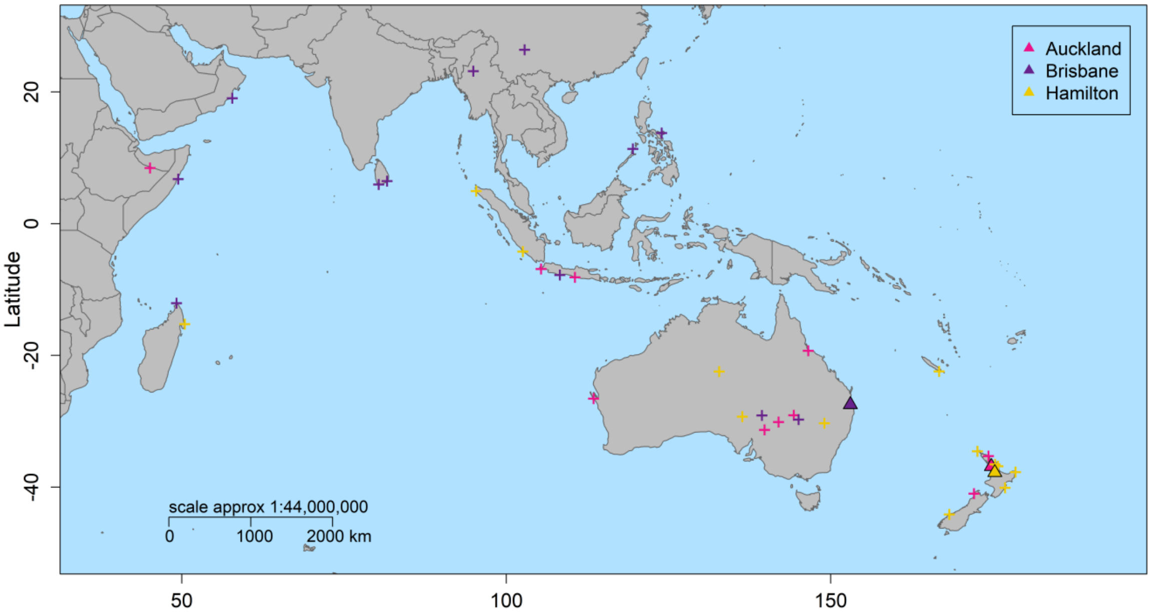

Figure S1

mGPS geographical predictions for the MetaSUB's global dataset per region. Figures A-F specifies the regional predictions (crosses) colored-coded according to the sampled city (triangle, see also the legend).

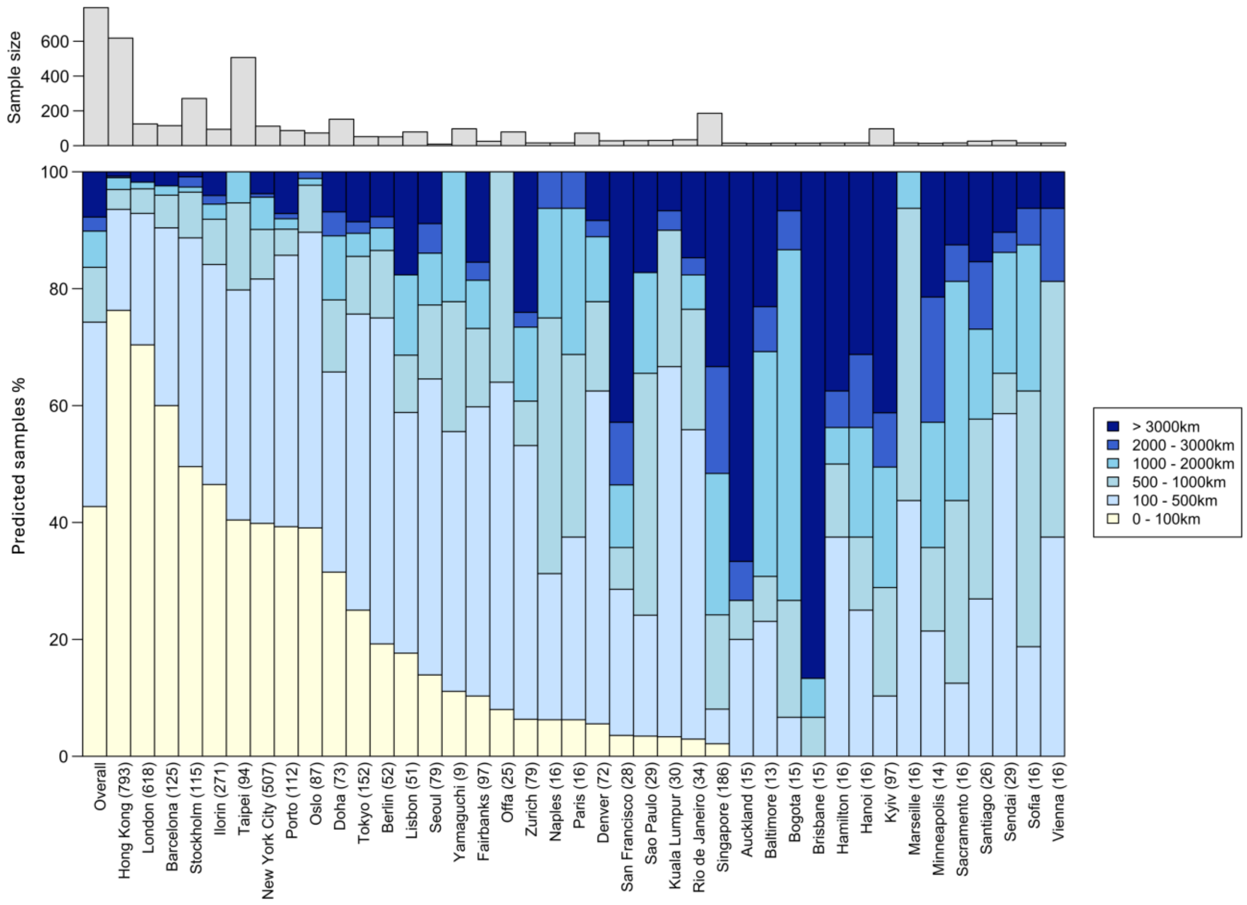

Figure S2

Accuracy between mGPS-predicted sources and sampling sites for the global MetaSub microbiome dataset. Sampling sites are shown as bar charts along the x-axis; the number of samples analyzed at each site is shown in brackets and is visualized as gray bars in the upper panel. The y-axis shows the accuracy of sample source prediction represented by six distance groups. Overall, ~43% of samples were predicted within less than 100km of their sampling site. The highest prediction accuracy was found in the Hong Kong dataset and the lowest in the Vienna dataset. The panel displays results for QCed sites and is sorted based on increasing inaccuracy from left to right.

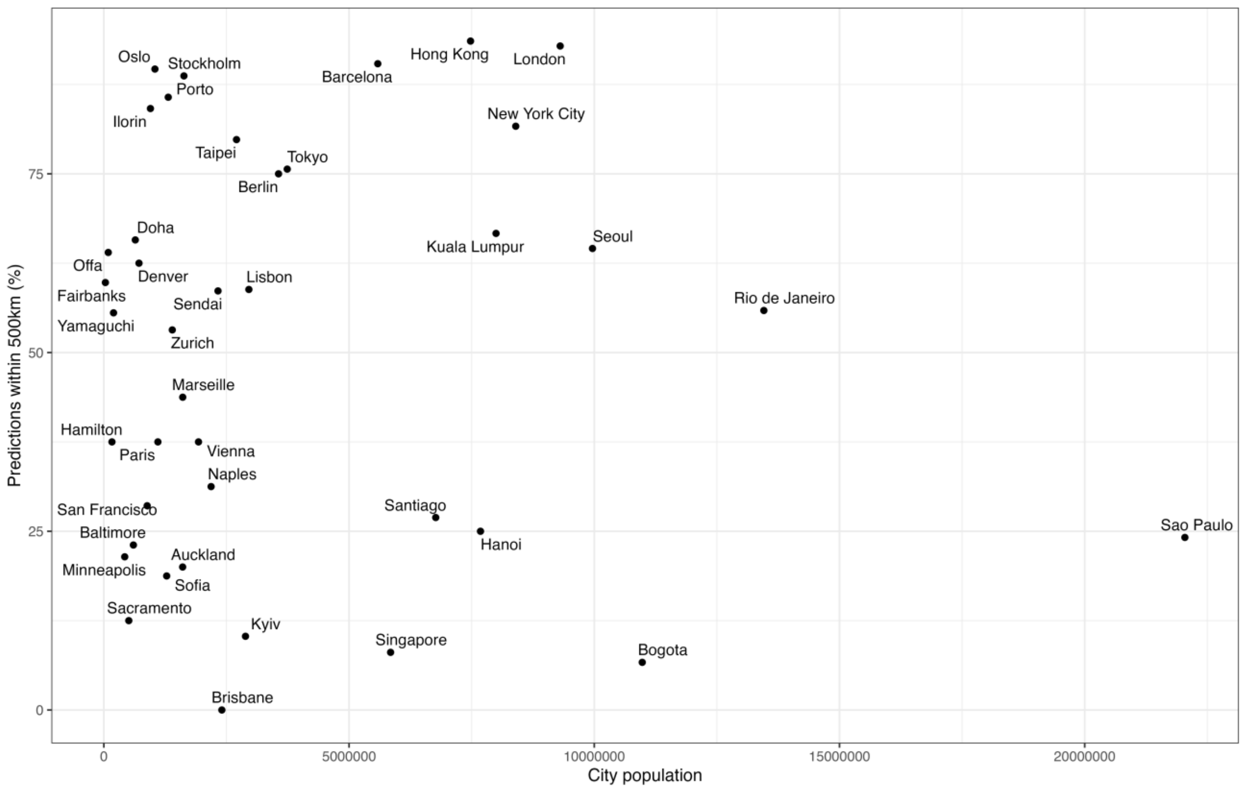

Figure S3

Distances between mGPS predicted sites and sampling sites per population size for the global MetaSub dataset. The population size is plotted against the proportion of samples predicted within 500km of the sampling site per city. There is no correlation between the geographical distance of the predicted and sampling sites and the city population size (*T-test*, n=40, r = 0.017, p-value=0.92, 95% *CI*=[-0.33, 0.30]).

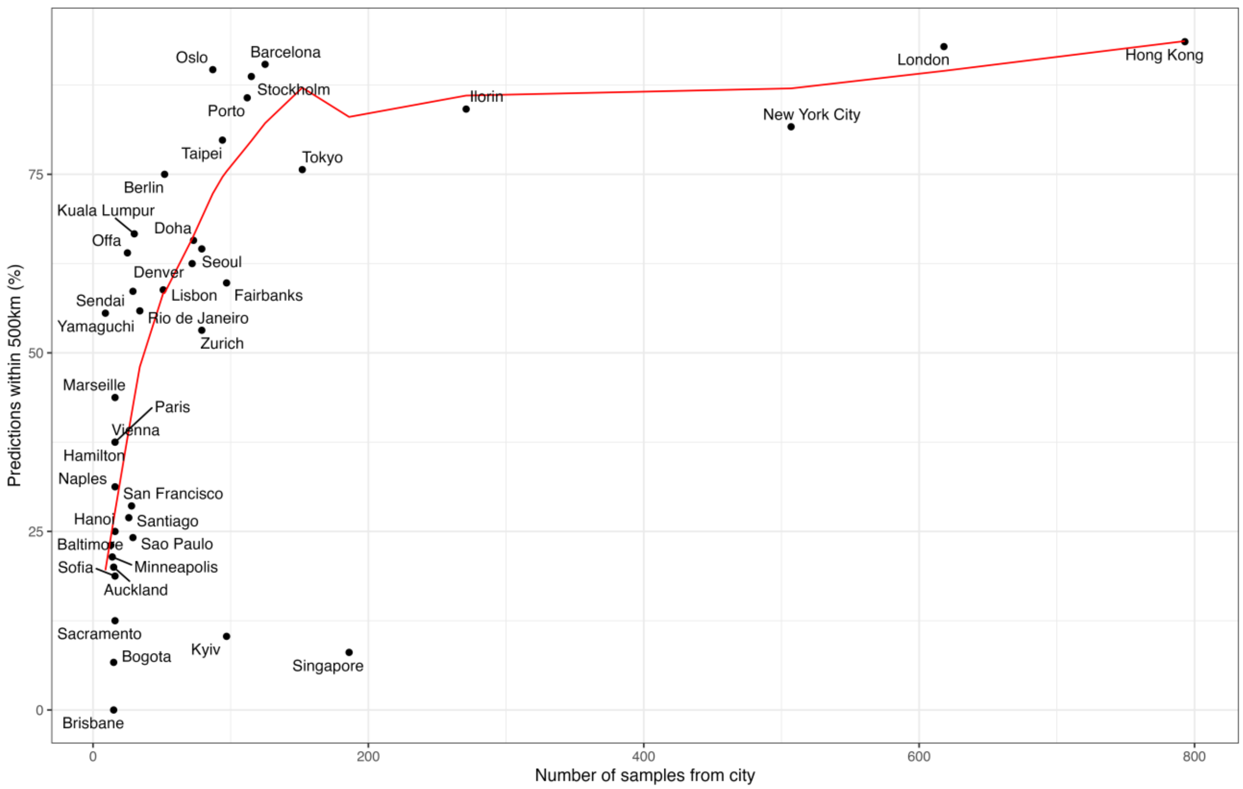

Figure S4

Distances between mGPS predicted sites and sampling sites for the global MetaSub dataset. The number of samples from each city present in our dataset is plotted against the geographical distance between mGPS predicted sites and the sampling sites per city. The shortest geographical distances between the predicted and sampling sites were found for cities that are well-represented in the training dataset (*T-test*, $n = 40$, $r = 0.53$, *p-value* = 0.0005, *CI* = [0.26, 0.72]).

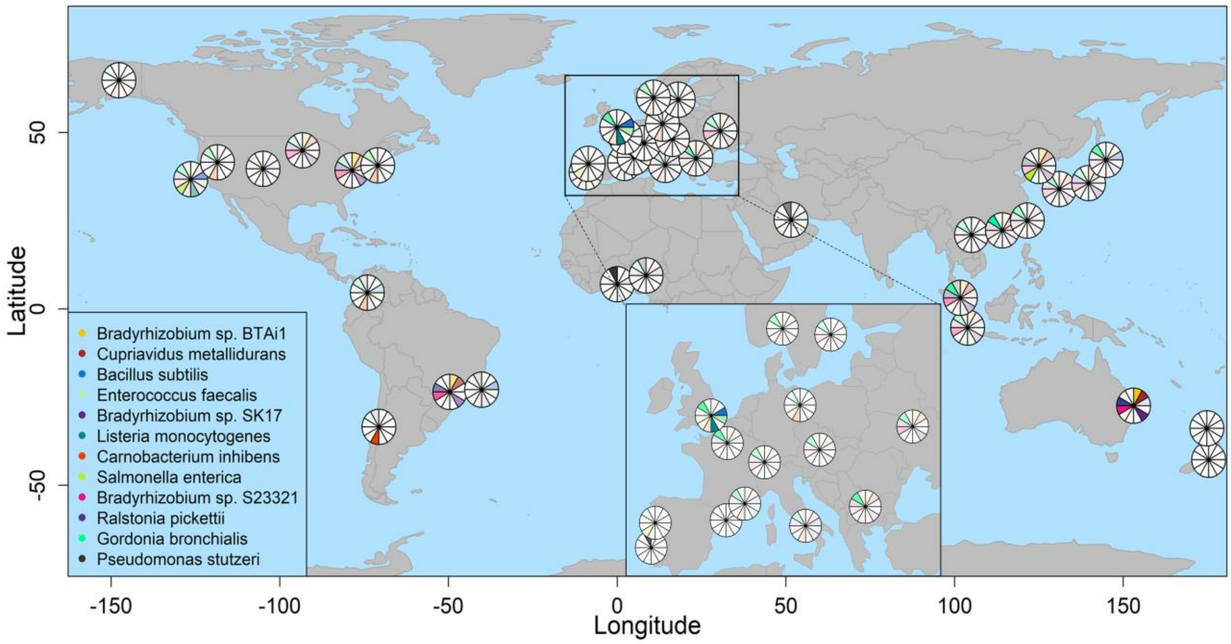

Figure S5

Differences in median taxa abundance between cities for the 12 most informative global MetaSUB GITs. The median city abundance of each taxon is normalized to represent the differences in relative sequence abundance between cities. For each city, each color in the pie chart corresponds to a taxon, and the shade of the color indicates the abundance of that taxon in this city. Darker colors for each taxon represent the taxon in that city with a high abundance relative to other cities.

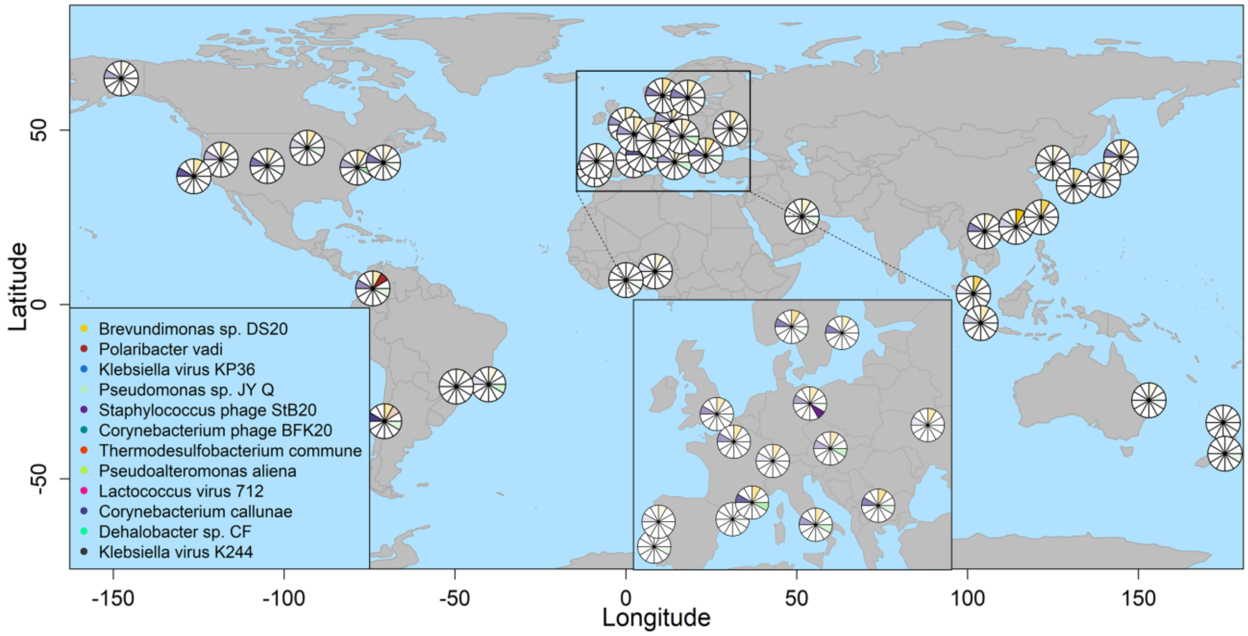

Figure S6

Differences in median taxa abundance between cities for 12 randomly selected taxa from the MetaSUB global analysis. The median city abundance of each taxon is normalized to represent the differences in relative sequence abundance between cities. For each city, each color in the pie chart corresponds to a taxon, and the shade of the color indicates the abundance of that taxon in this city. Darker colors for each taxon represent the taxon in that city with a high abundance relative to other cities.

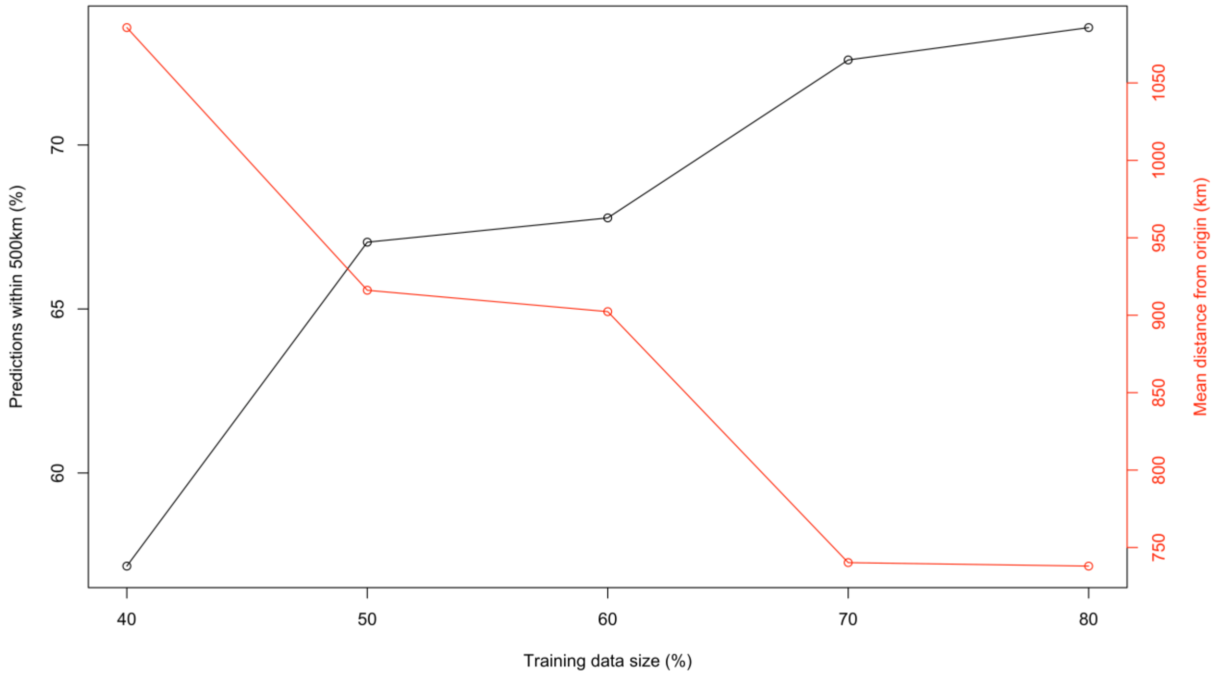

Figure S7

Global MetaSub prediction distances on a holdout test set consisting of 20% of the full MetaSUB dataset with proportional representation of all cities. The increasing proportions of the remaining data were used to train the model before generating predictions for the holdout set, i.e., the initial 80% represents the entire training data, which is sliced in each stage. The geographical distance between the predicted and sampling sites depends on the number of training samples.

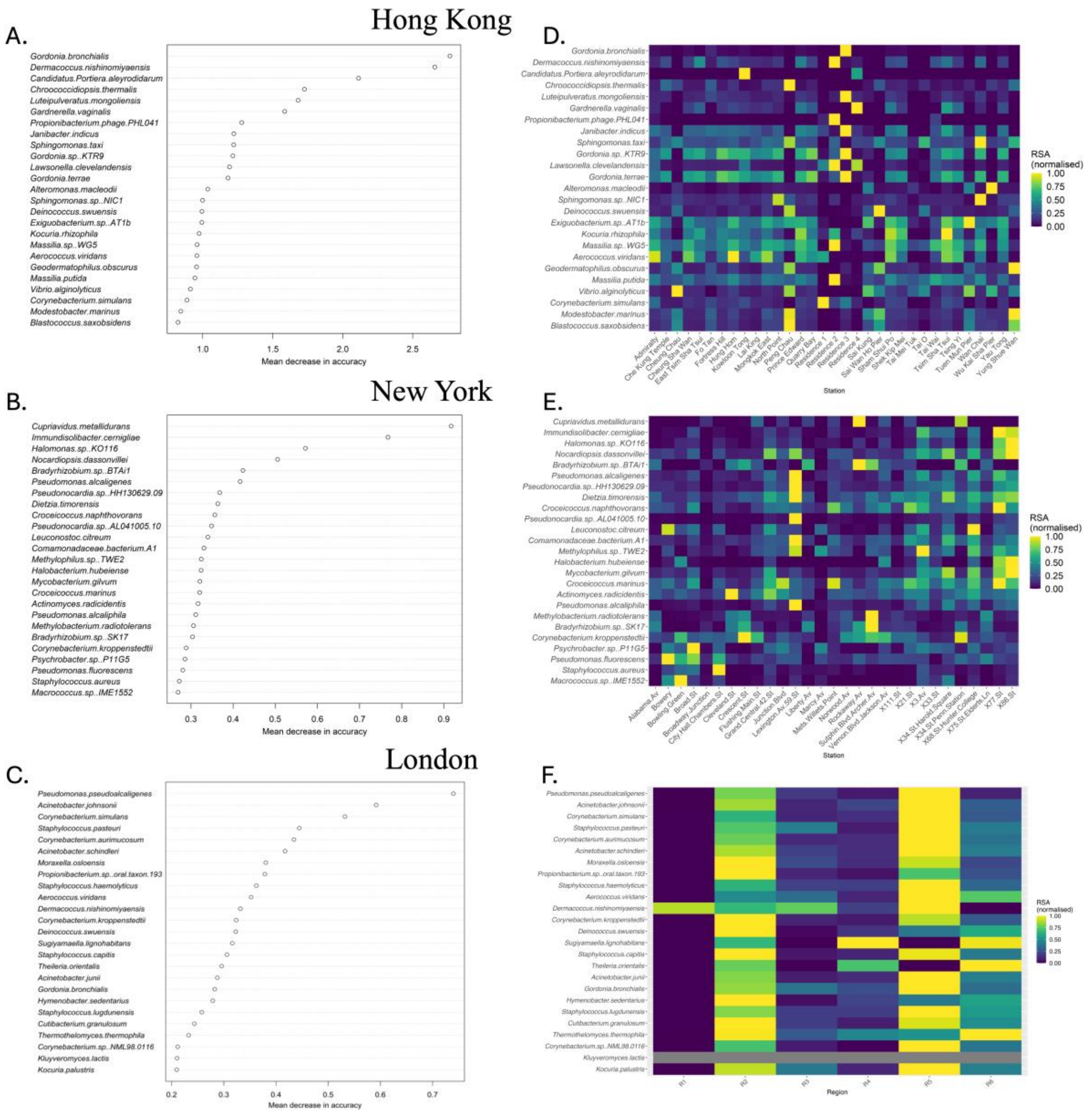

Figure S8

The top 25 GIT (A, B, and C) and the geographic differences in median taxa abundance between transit stations (D, E, and F) for the three highly sampled MetaSUB cities. The variable importance (mean decrease accuracy) plot for the random forest classifier for the soil microbiome data (A, B, and C) shows the importance of the top 25 GIT (variables) for microbiome geographical predictions. The prediction accuracy is first calculated using the entire testing dataset (out-of-bag data). Then, the abundance values for one species are randomized, and the prediction accuracy is calculated again. The difference between these two measures is the mean decrease in accuracy (*x*-axis). For each city, the heatmap contains the top 25 GIT (rows) and all sampled stations (columns). The median station abundance of each taxon is normalized to represent the differences in relative abundance between stations. The normalized abundance values range from 0 (very dark blue) to 1 (bright yellow), the latter representing stations with a higher abundance of the taxon relative to other stations. The row is shaded grey in F because this taxon appeared in 22% of samples with low RSA and a median value of 0 across all regions, and standardization cannot be conducted.

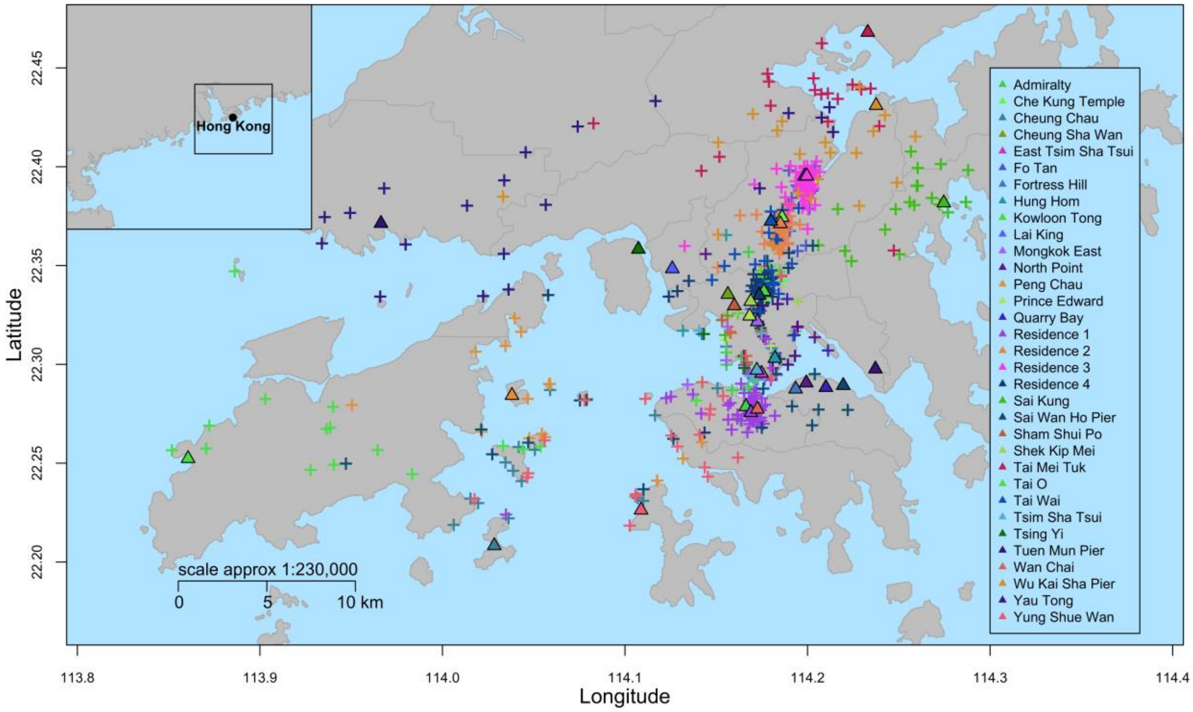

Figure S9

mGPS predictions for Hong Kong microbiomes. Markers show the predicted location coordinates for all Hong Kong samples from the MetaSUB dataset. Marker colors correspond to their sampling station. Triangles represent all 33 sampled stations (legend).

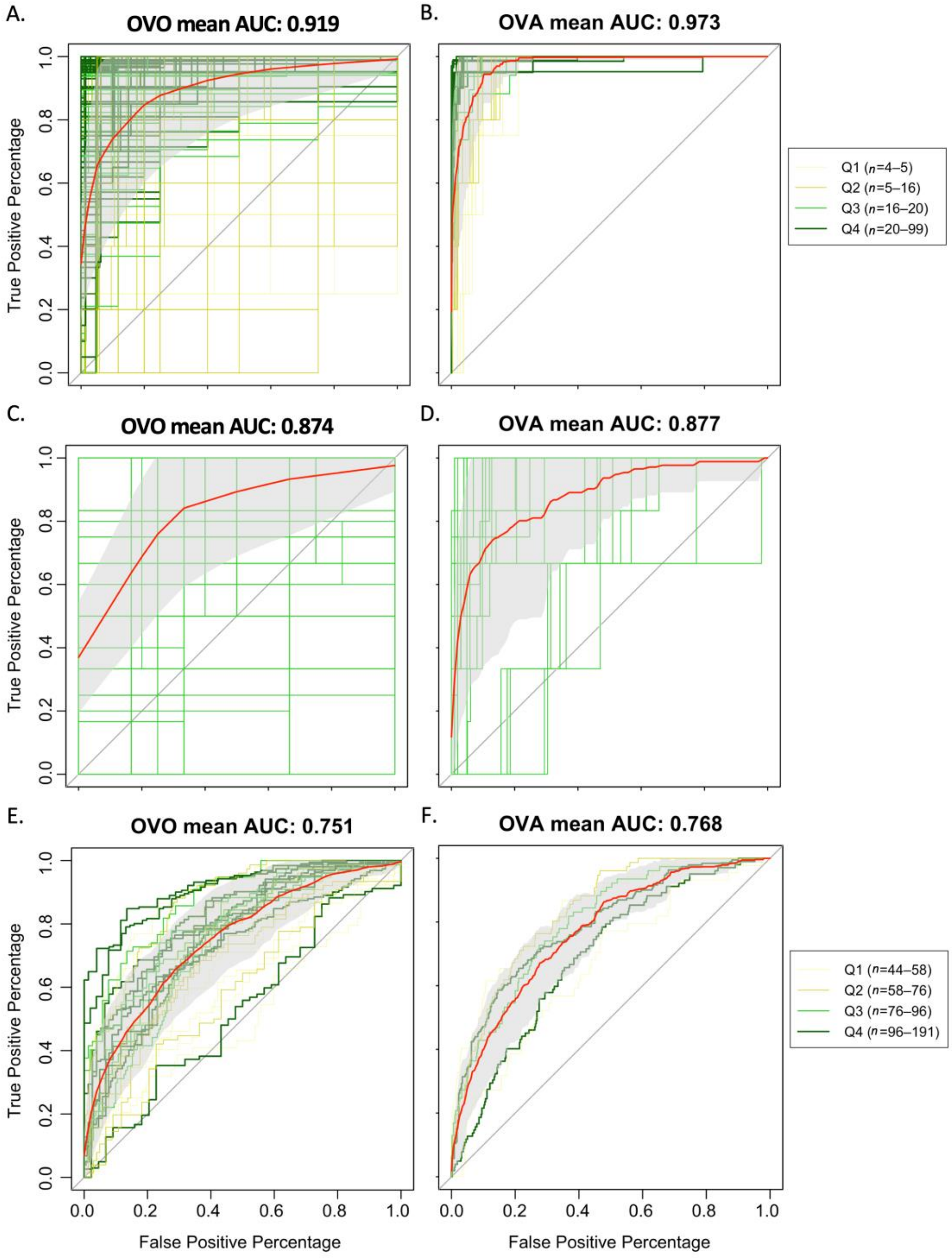

Figure S10

ROC curves for mGPS classification of MetaSub samples within three cities: Hong Kong (A–B), New York (C–D), and London (E–F) using the OVO (A, C, and E) and OVA (B, D, and F) approaches. OVO approach compares all possible two-class combinations of the dataset. OVA approach compares each class against all the others. The ROC curves for each comparison group are color-coded according to the quartile sample size (*n*) of the continents or cities (see legend), except in New York City, where *n* ranged from *3–6*. The red line shows the mean ROC curve. The grey background represents one standard deviation of the mean. The horizontal line represents an AUC of 0.5.

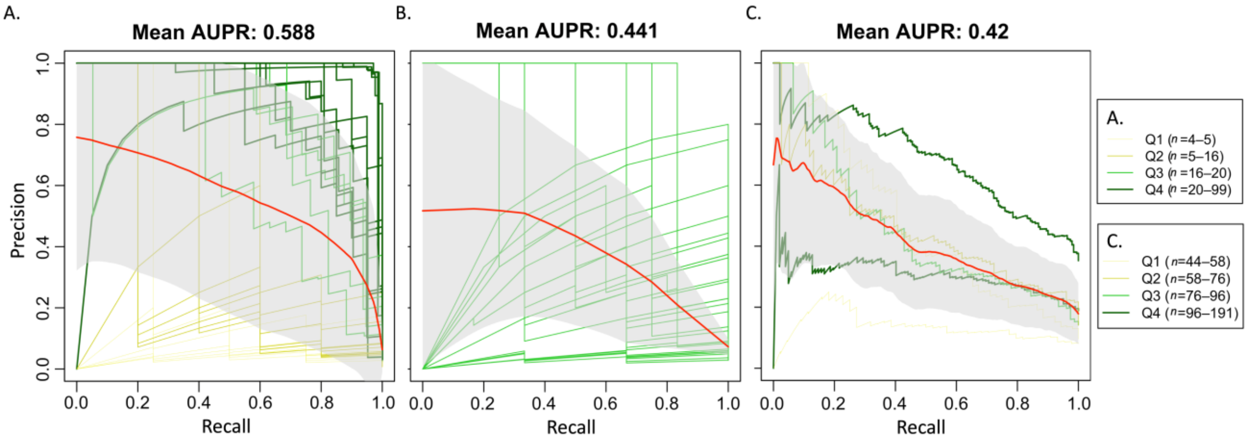

Figure S11

PR curves for mGPS classification of stations or super stations in the MetaSub three sub-cities Hong Kong (A), New York (B), and London (C). The curves for each comparison group are color-coded according to the quartile sample size (*n*) of the continents or cities (legend), except in New York City, where *n* ranged from *3–6*. The red line shows the mean ROC curve. The grey background represents one standard deviation of the mean. The horizontal line represents an AUC of 0.5.

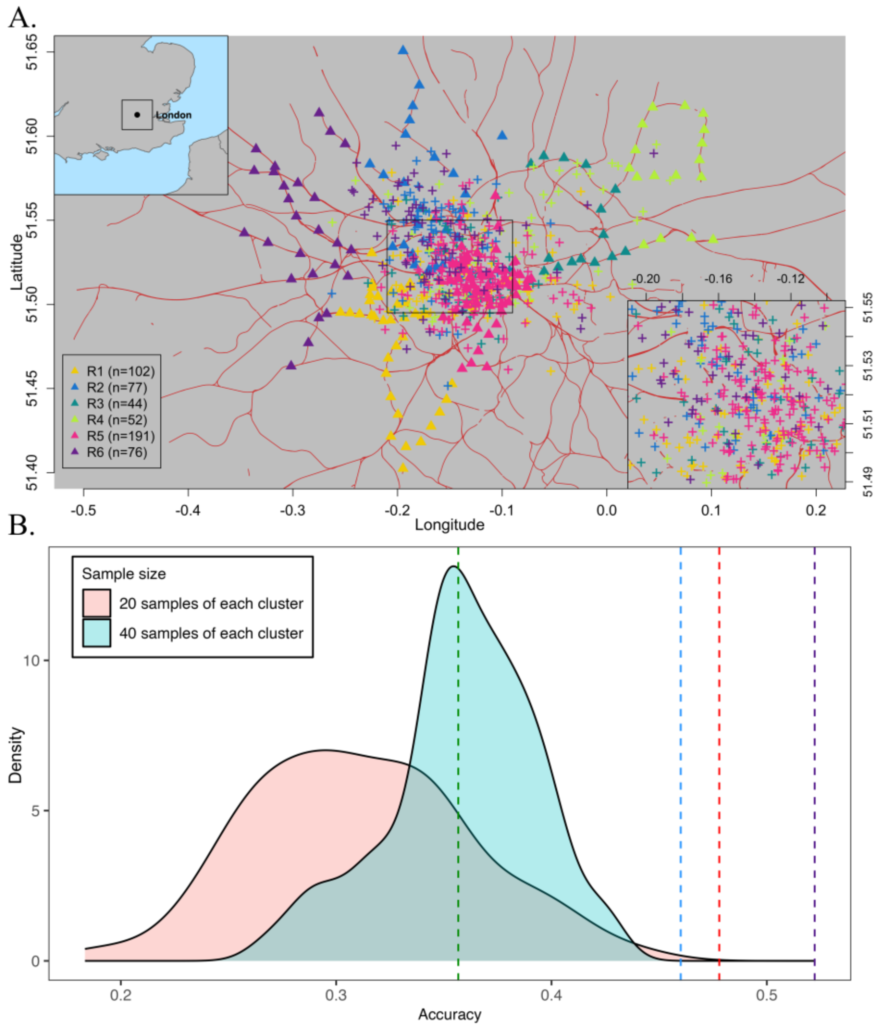

Figure S12

mGPS predictions for London microbiomes for the original dataset (A) and random subsets (B). A. Points represent all predicted locations for London MetaSUB data. Triangles denote all sampled stations, while '+' symbols indicate the predicted samples. To handle the lack of regional boundaries and the varied geographical density of stations, we applied *k*-means clustering (*K*=6) to station coordinates (see legends for the counts). Stations and their corresponding predictions were color-coded based on these clusters. The top left inset depicts the sampling area. The bottom right inset shows predictions for the central region. B. To assess its prediction accuracy across different sample size subsets, the model was trained using 20 and 40 samples randomly selected from each cluster, repeated 100 times. Accuracy distributions were plotted for each cohort. Dashed lines represent model accuracy for subsets randomly selected from each of the six clusters (R1-6), distinguished by colors: green (100:40:40:40:40:40), blue (40:40:40:40:100:40), and purple (40:40:40:40:160:40), which can be compared with the accuracy of the original dataset (in red). These results demonstrate that the mGPS predictor is not always biased towards regions with larger sample sizes. It is also essential to consider the specific characteristics of the samples within the regions. For instance, the R5 sample, which is positioned in the central region as a large cluster (green line), exhibits higher accuracy compared to the R1 sample as a large cluster (blue line). These results demonstrate that the mGPS predictor is unbiased towards regions with larger sample sizes and that using larger sample sizes (over even sample sizes) enhances prediction accuracy.

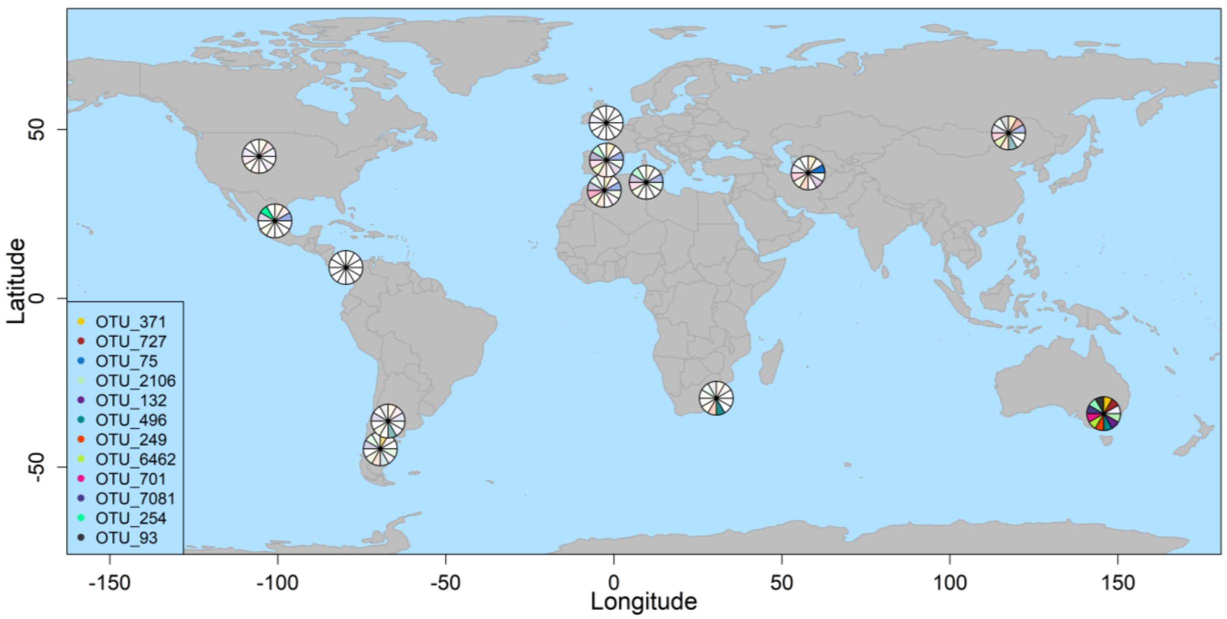

Figure S13

Differences in median taxa abundance between countries for the 12 most informative soil microbiome GITs. The median country abundance of each taxon is normalized to represent the differences in relative sequence abundance between countries. For each country, each color in the pie chart corresponds to a taxon, and the shade of the color indicates the abundance of that taxon in this country. Darker colors for each taxon represent the taxon in that country with a high abundance relative to other countries.

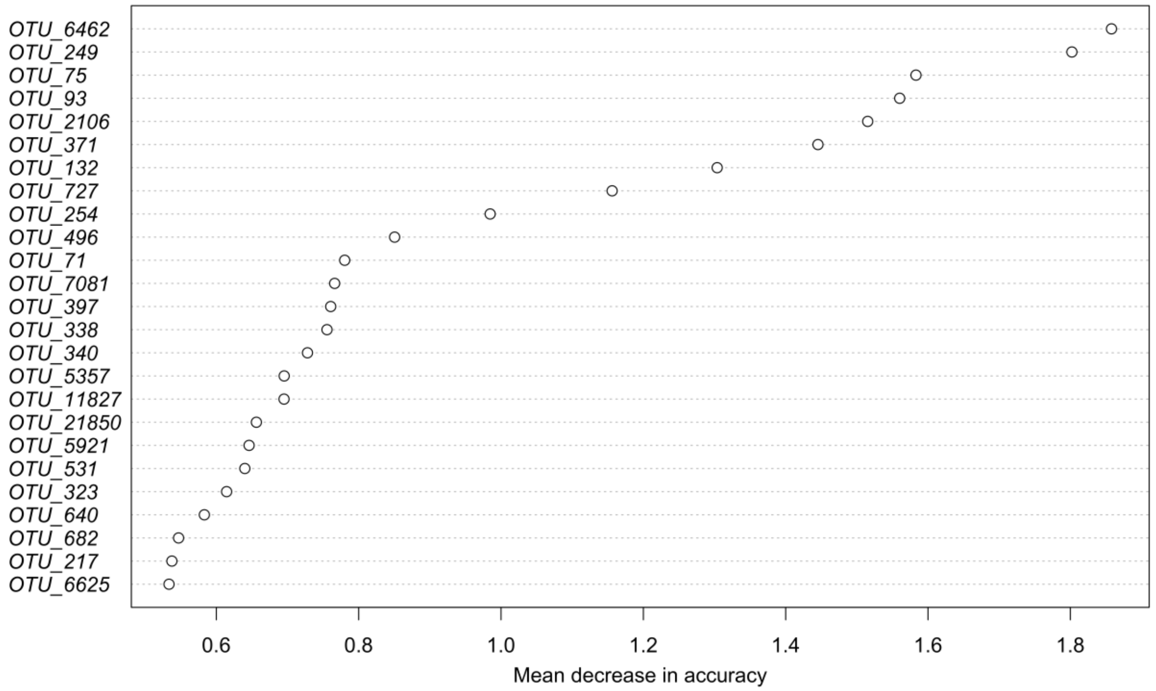

Figure S14

Variable importance (mean decrease accuracy) plot for the random forest classifier for the soil microbiome data. The plot shows the importance of the top 25 GIT (variables) for global soil microbiome geographical predictions. The prediction accuracy is first calculated using the entire testing dataset (out-of-bag data). Then, the abundance values for one species are randomized, and the prediction accuracy is calculated again. The difference between these two measures is the mean decrease in accuracy (*x*-axis). In other words, the highest *x*-value indicates that *OTU_6462* has more influence on the prediction accuracy of the testing dataset than any other species.

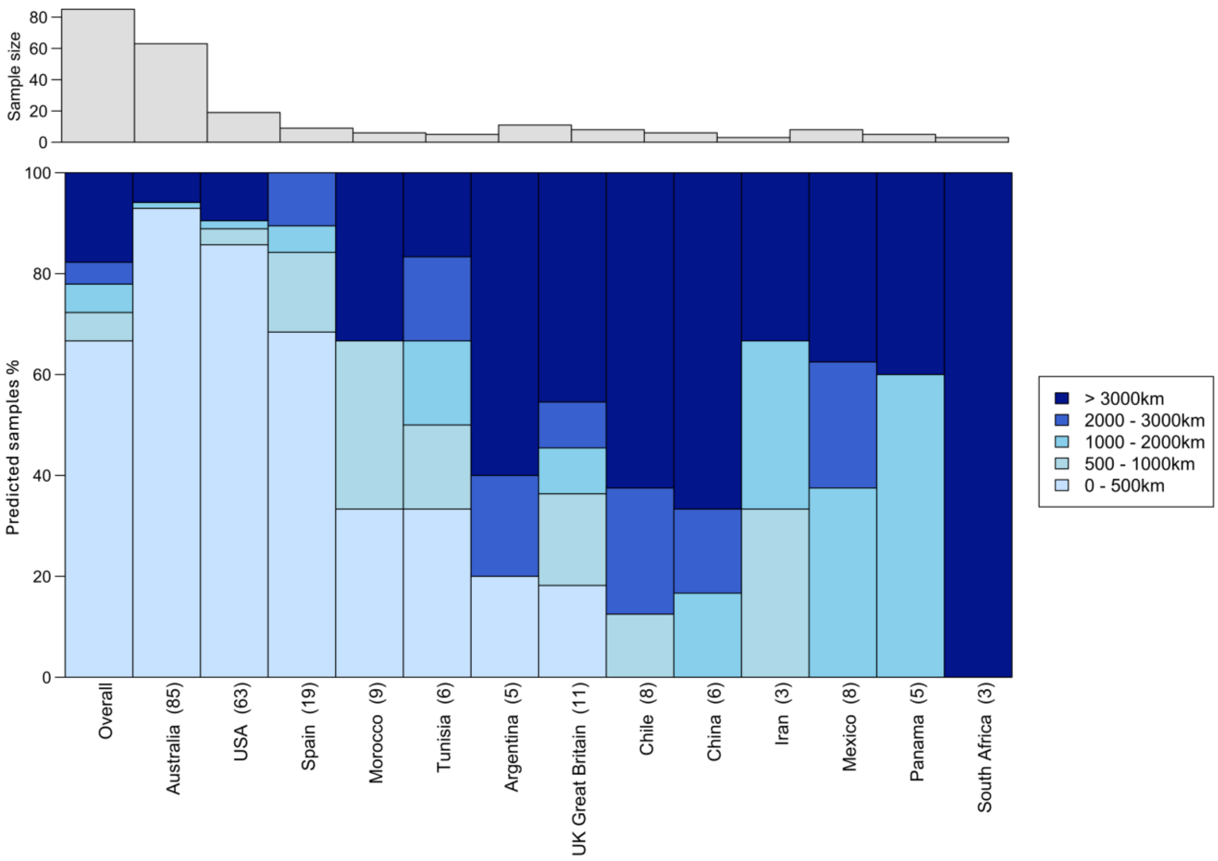

Figure S15

Accuracy between mGPS-predicted sources and sampling sites for the soil microbiome dataset. Sampling sites are shown as bar charts along the x-axis. The number of samples analyzed at each site is shown in brackets and is visualized as gray bars in the upper panel. The y-axis shows the accuracy of sample source prediction represented by six distance groups. Overall, ~70% of samples were predicted within less than 500km of their sampling site. The highest prediction accuracy was found in the Australian dataset and the lowest in the Panama dataset. The panel displays results for QCed sites and is sorted based on increasing inaccuracy from left to right.

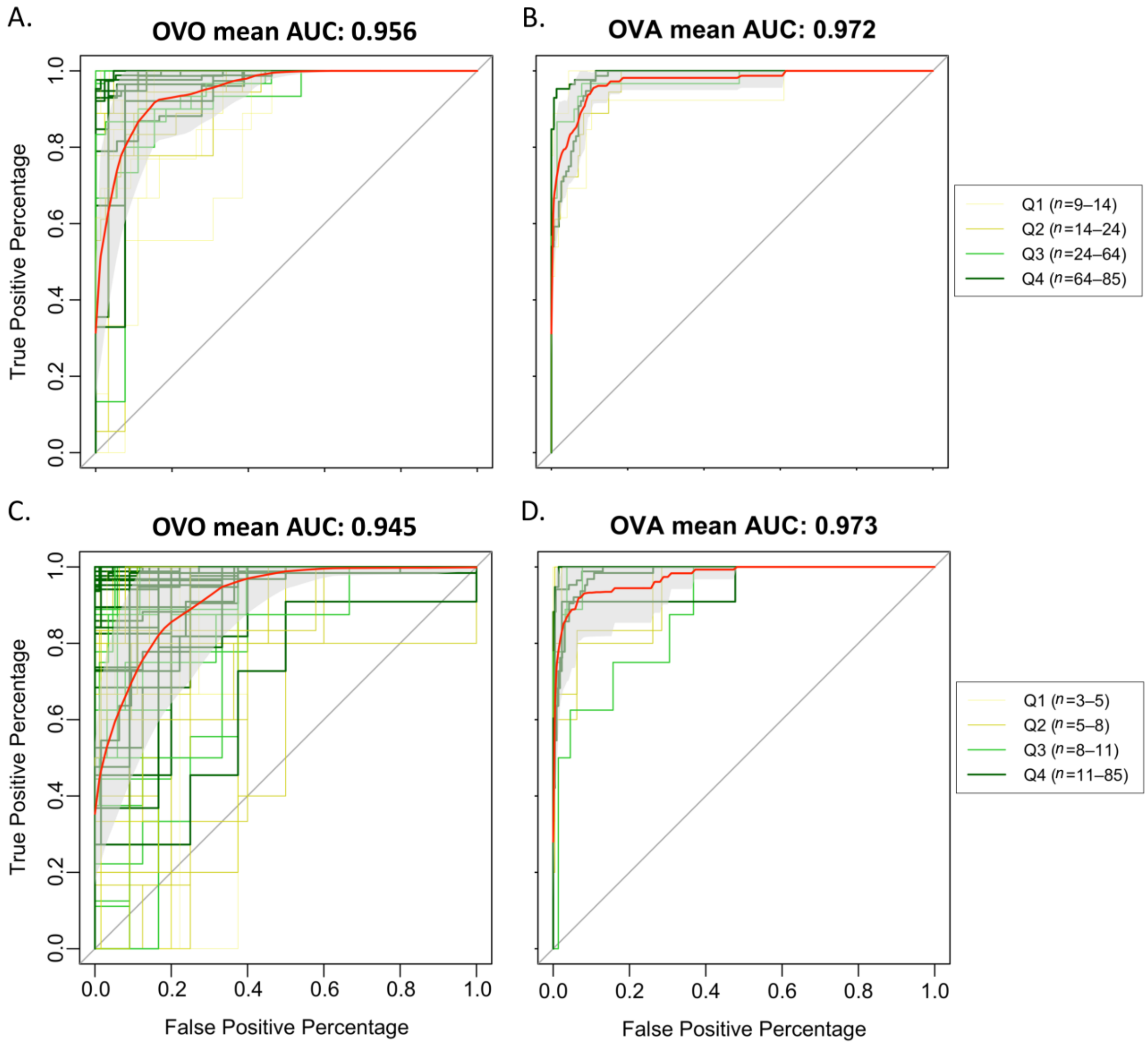

Figure S16

ROC curves for mGPS classification of Soil samples to continents (A–B) and cities (C–D) using the OVO (A, C) and OVA (B, D) approaches. OVO approach compares all possible two-class combinations of the dataset. OVA approach compares each class against all the others. The ROC curves for each comparison group are color-coded according to the quartile sample size (*n*) of the continents or cities (see legend). The red line shows the mean ROC curve. The grey background represents one standard deviation of the mean. The horizontal line represents an AUC of 0.5.

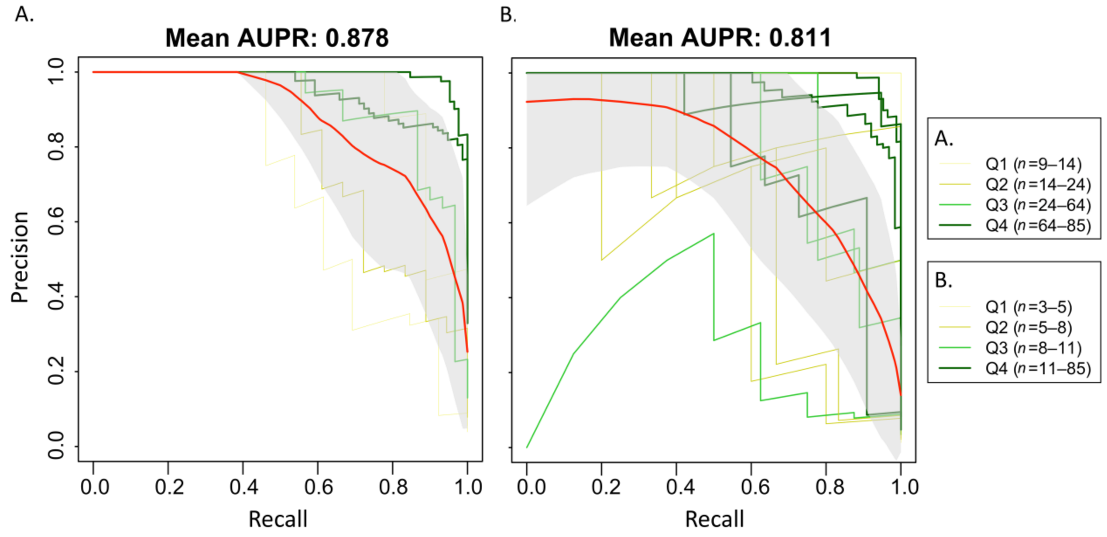

Figure S17

PR curves for mGPS classification of soil samples to continents (A) and countries (B). The curves for each comparison group are color-coded according to the quartile sample size (*n*) of the continents or cities (see legend). The grey background represents one standard deviation of the mean. The horizontal line represents an AUC of 0.5.

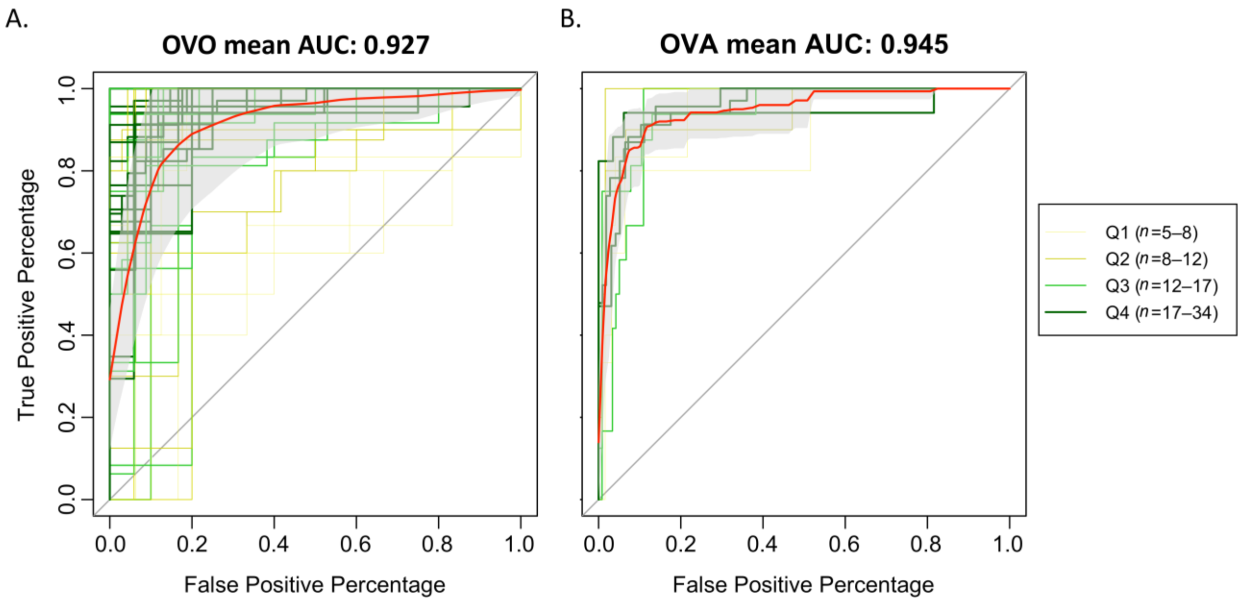

Figure S18

ROC curves for mGPS classification of marine samples to water bodies using the OVO (A) and OVA (B) approaches. OVO approach compares all possible two-class combinations of the dataset. OVA approach compares each class against all the others. The ROC curves for each comparison group are color-coded according to the quartile sample size (*n*) of the continents or cities (see legend). The red line shows the mean ROC curve. The grey background represents one standard deviation of the mean. The horizontal line represents an AUC of 0.5.

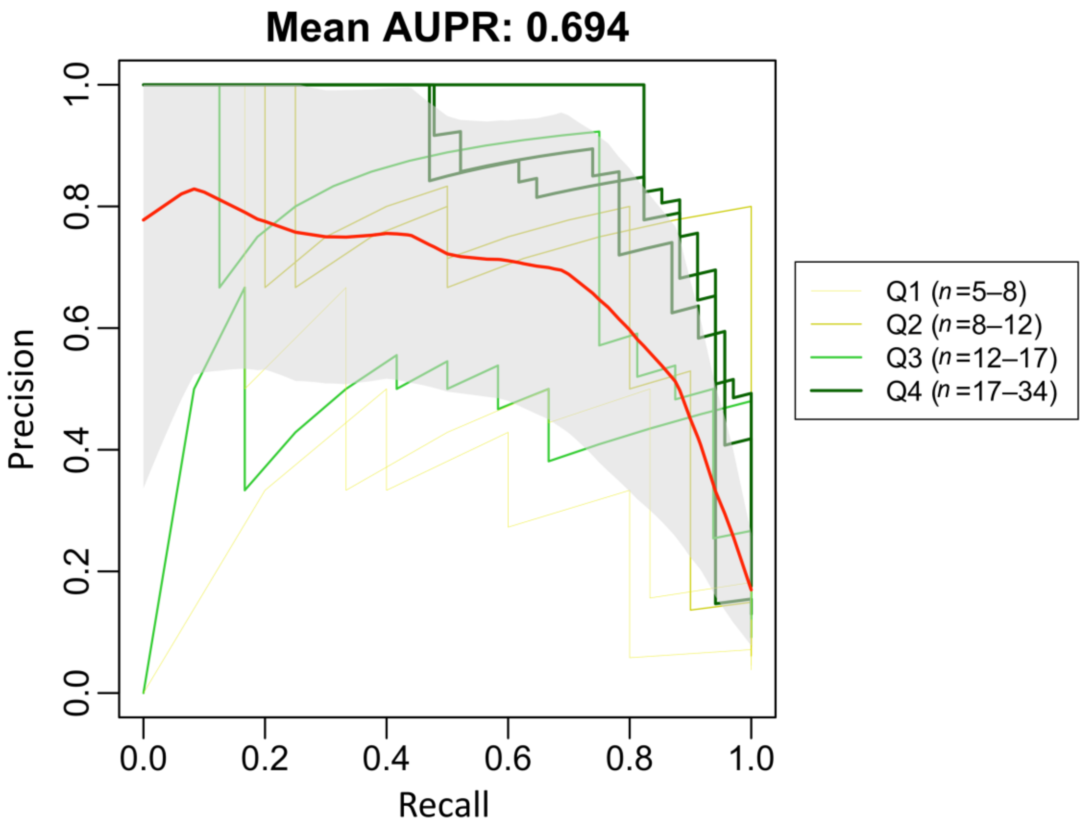

Figure S19

PR curves for mGPS classification of marine samples to water bodies. The curves for each comparison group are color-coded according to the quartile sample size (*n*) of the continents or cities (see legend). The grey background represents one standard deviation of the mean. The horizontal line represents an AUC of 0.5.

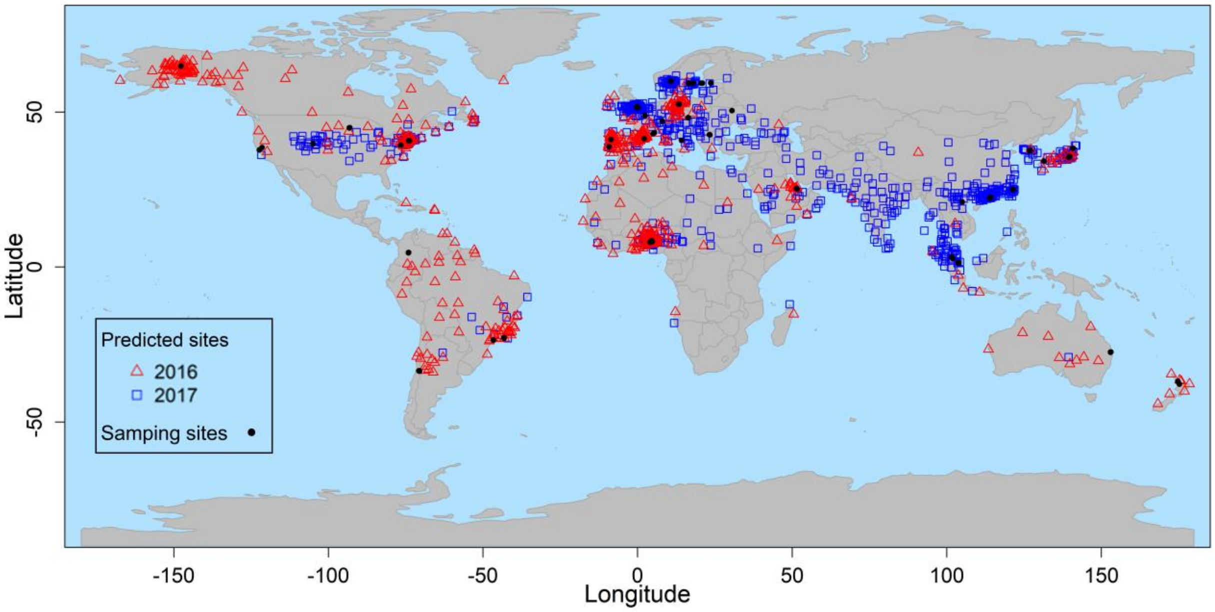

Figure S20
mGPS-predicted sites for AMR gene transfer between 2016 and 2017. mGPS predicted a flow of AMR genes from the colored symbols to the sampling sites (black circles). This is animated on a timeline in AMR_transmission.mp4.

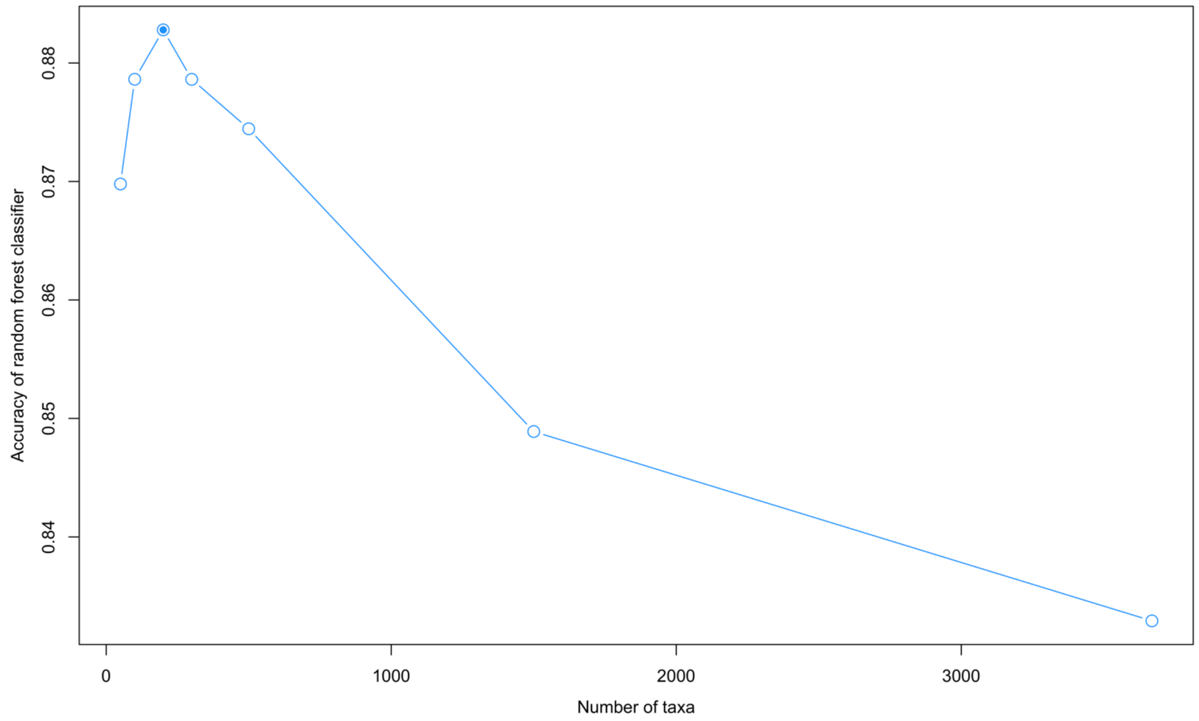

Figure S21

Accuracy of random-forest city classifier used for recursive feature elimination for different-sized MetaSUB subsets of the most important variables (species). The results indicate that many taxa do not exhibit geographic information clustering and cannot improve the model prediction. A smaller subset of 200 species as predictor variables (GIT) yields the highest prediction accuracy.

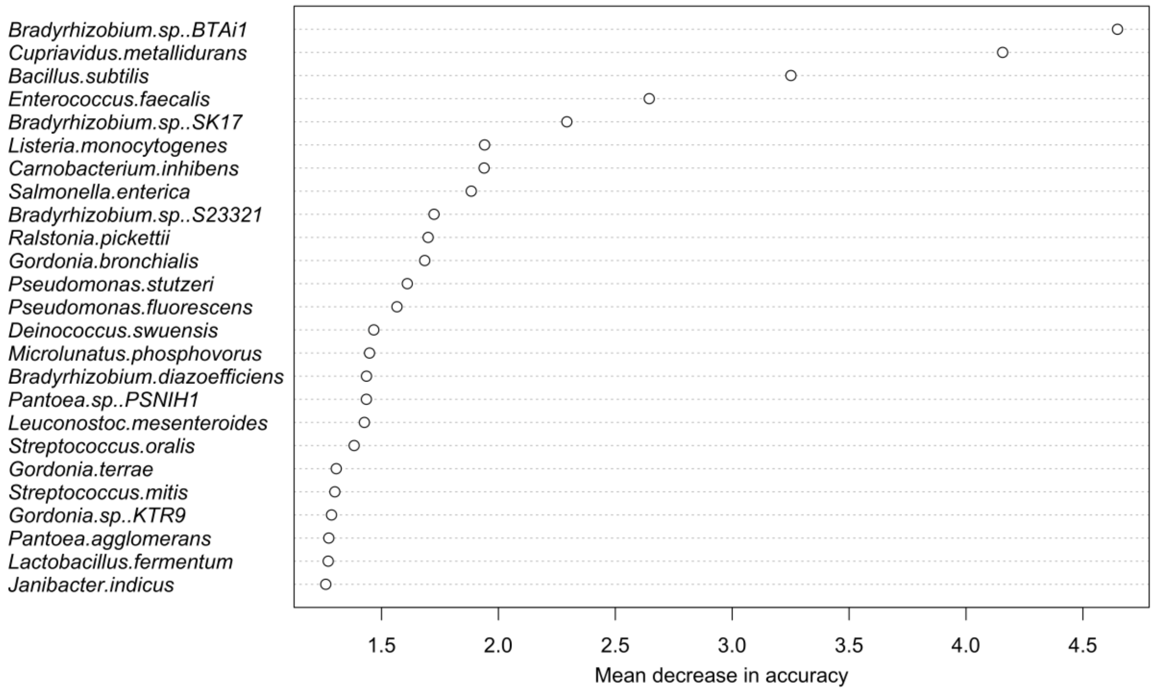

Figure S22

Variable importance (mean decrease accuracy) plot for the random forest classifier for the MetaSUB microbiome data. The plot shows the importance of the top 25 GIT (variables) for global MetaSUB microbiome geographical predictions. The prediction accuracy is first calculated using the entire testing dataset (out-of-bag data). Then, the abundance values for one species are randomized, and the prediction accuracy is calculated again. The difference between these two measures is the mean decrease in accuracy (x-axis). In other words, the highest x-value indicates that *Bradyrhizobium sp. BTAi1* has more influence on the prediction accuracy of the testing dataset than any other species.

Table S1A

Prediction statistics for the global MetaSUB predictions for the city of origin. The F1 score measures the percentage of predictions made to the sampling sites. Balanced accuracy is the arithmetic mean of sensitivity and specificity.

City	Sample size	Sensitivity	Specificity	Precision	F1	Prevalence	Balanced accuracy
Auckland	15	0.27	1	0.27	0.27	0	0.63
Baltimore	13	0.46	1	0.86	0.6	0	0.73
Barcelona	125	0.99	1	0.98	0.99	0.03	1
Berlin	52	0.88	1	0.92	0.9	0.01	0.94
Bogota	15	0.87	1	1	0.93	0	0.93
Brisbane	15	0.67	1	0.83	0.74	0	0.83
Denver	72	0.9	1	0.87	0.88	0.02	0.95
Doha	73	0.95	1	0.87	0.91	0.02	0.97
Fairbanks	97	0.89	1	0.85	0.87	0.02	0.94
Hamilton	16	0.5	1	0.53	0.52	0	0.75
Hanoi	16	0.69	1	0.92	0.79	0	0.84
Hong Kong	793	0.99	0.99	0.96	0.97	0.19	0.99
Ilorin	271	0.96	1	0.94	0.95	0.07	0.98
Kuala Lumpur	30	0.93	1	1	0.97	0.01	0.97
Kyiv	97	0.62	0.99	0.69	0.65	0.02	0.81
Lisbon	51	0.82	1	0.82	0.82	0.01	0.91
London	618	0.98	1	0.98	0.98	0.15	0.99
Marseille	16	0.81	1	0.93	0.87	0	0.91
Minneapolis	14	0.71	1	0.71	0.71	0	0.86
Naples	16	0.62	1	0.77	0.69	0	0.81
New York	507	0.97	0.99	0.94	0.96	0.12	0.98
Offa	25	0.72	1	0.9	0.8	0.01	0.86
Oslo	87	0.93	1	0.96	0.95	0.02	0.97
Paris	16	0.5	1	0.67	0.57	0	0.75
Porto	112	0.93	1	0.97	0.95	0.03	0.96
Rio de Janeiro	34	0.85	1	0.94	0.89	0.01	0.93
Sacramento	16	0.81	1	0.93	0.87	0	0.91
San Francisco	28	0.86	1	0.92	0.89	0.01	0.93
Santiago	26	0.88	1	0.92	0.9	0.01	0.94
Sao Paulo	29	0.9	1	0.9	0.9	0.01	0.95
Sendai	29	0.83	1	0.96	0.89	0.01	0.91
Seoul	79	0.89	1	0.93	0.91	0.02	0.94
Singapore	186	0.85	0.99	0.78	0.82	0.05	0.92
Sofia	16	0.69	1	1	0.81	0	0.84
Stockholm	119	0.97	1	0.93	0.95	0.03	0.98
Taipei	94	0.95	1	0.98	0.96	0.02	0.97
Tokyo	152	0.9	1	0.88	0.89	0.04	0.95
Vienna	16	0.69	1	0.79	0.73	0	0.84
Yamaguchi	9	0	1	NA	NA	0	0.5
Zurich	79	0.66	0.99	0.71	0.68	0.02	0.83

Table S1B

Prediction statistics for the global MetaSUB predictions for the smallest cities of origin (<100 samples). The F1 score measures the percentage of predictions made to the sampling sites. Balanced accuracy is the arithmetic mean of sensitivity and specificity.

City	Sample size	Sensitivity	Specificity	Precision	F1	Prevalence	Balanced Accuracy
Auckland	15	0.4	0.99	0.5	0.44	0.01	0.7
Baltimore	13	0.62	1	0.89	0.73	0.01	0.81
Berlin	52	0.88	0.99	0.88	0.88	0.04	0.94
Bogota	15	0.87	1	1	0.93	0.01	0.93
Brisbane	15	0.67	1	0.77	0.71	0.01	0.83
Denver	72	0.92	0.99	0.9	0.91	0.06	0.96
Doha	73	0.96	0.99	0.89	0.92	0.06	0.98
Fairbanks	97	0.93	0.99	0.89	0.91	0.08	0.96
Hamilton	16	0.56	0.99	0.56	0.56	0.01	0.78
Hanoi	16	0.88	1	0.82	0.85	0.01	0.94
Kuala Lumpur	30	0.87	1	1	0.93	0.03	0.93
Kyiv	97	0.87	0.98	0.77	0.82	0.08	0.92
Lisbon	51	0.96	1	0.91	0.93	0.04	0.98
Marseille	16	0.81	1	0.87	0.84	0.01	0.91
Minneapolis	14	0.79	1	0.85	0.81	0.01	0.89
Naples	16	0.56	1	0.82	0.67	0.01	0.78
Offa	25	0.88	1	1	0.94	0.02	0.94
Oslo	87	0.99	1	0.95	0.97	0.07	0.99
Paris	16	0.63	1	0.77	0.69	0.01	0.81
Rio De Janeiro	34	0.94	1	0.97	0.96	0.03	0.97
Sacramento	16	0.81	1	0.93	0.87	0.01	0.91
San Francisco	28	0.82	1	0.85	0.84	0.02	0.91
Santiago	26	0.96	1	0.86	0.91	0.02	0.98
Sao Paulo	29	0.97	1	0.97	0.97	0.02	0.98
Sendai	29	1	1	0.97	0.98	0.02	1
Seoul	79	0.94	0.99	0.89	0.91	0.07	0.96
Sofia	16	0.69	1	1	0.81	0.01	0.84
Taipei	94	0.99	1	0.95	0.97	0.08	0.99
Vienna	16	0.63	1	0.71	0.67	0.01	0.81
Yamaguchi	9	0.56	1	0.71	0.63	0.01	0.78
Zurich	79	0.72	0.98	0.74	0.73	0.07	0.85

Table S2

A confusion matrix for mGPS predictions for 131 marine microbiome samples.

		Observed								
		Arabian Sea n=10	*Indian Ocean* n=12	*Mediterranean Sea* n=8	*Mozambique Channel* n=5	*North Atlantic Ocean* n=17	*North Pacific Ocean* n=16	*Red Sea* n=6	*South Atlantic Ocean* n=23	*South Pacific Ocean* n=34
**Predicted**	*Arabian Sea*	8	0	0	0	0	0	0	0	0
	Indian Ocean	1	6	0	1	0	0	2	0	1
	Mediterranean Sea	0	0	7	0	1	1	0	0	0
	Mozambique Channel	0	2	0	3	0	0	0	0	0
	North.Atlantic Ocean	0	0	1	0	13	1	0	2	0
	North Pacific Ocean	0	0	0	0	0	11	0	1	1
	Red Sea	1	0	0	1	0	0	3	0	0
	South Atlantic Ocean	0	2	0	0	1	0	0	18	4
	South Pacific Ocean	0	2	0	0	2	3	1	2	28

Table S3
Annual tourist numbers for each city in the MetaSUB dataset.

City	Number of tourists annually	Citation	Description
Auckland	3800000	https://camperchamp.com.au/statistics/new-zealand/	
Baltimore	26200000	https://www.bizjournals.com/baltimore/news/2018/09/25/baltimore-drew-more-than-26-million-visitors-last.html	
Barcelona	32000000	https://www.responsibletravel.com/copy/overtourism-in-barcelona	
Berlin	129700000	https://www.statista.com/statistics/568463/tourism-arrivals-berlin-germany-by-origin/	
Bogota	4500000	https://thecitypaperbogota.com/news/colombia-saw-record-tourism-in-2019-with-4-5-million-visitors/23974	
Brisbane	9100000	https://camperchamp.com.au/statistics/australia/queensland-tourism/	
Denver	31000000	https://www.denver.org/articles/post/denver-tourism-repeats-31m-visitors-in-2018/	
Doha	1800000	https://www.statista.com/statistics/1016135/qatar-overnight-tourists-number/	
Fairbanks	NA	NA	Data not available
Hamilton	6000000	https://investinhamilton.ca/industries/tourism-2/	
Hanoi	28000000	https://www.vietnam-briefing.com/news/vietnams-tourism-industry-continues-growth-2018.html/	
Hong Kong	56000000	https://www.reuters.com/article/us-health-coronavirus-hongkong-tourism/hong-kong-worlds-most-visited-city-faces-tourism-bust-idUSKBN28003Y	
Ilorin	NA	NA	Data not available
Kuala Lumpur	13434300	https://www.worlddata.info/asia/malaysia/tourism.php	
Kyiv	1600000	http://www.xinhuanet.com/english/2018-02/08/c_136959947.htm	
Lisbon	4500000	http://www.xinhuanet.com/english/2018-04/05/c_137088807.htm	
London	30000000	https://www.condorferries.co.uk/uk-tourism-statistics	
Marseille	4100000	https://web.archive.org/web/20130511062726/http://www.marseille.fr/sitevdm/decouvrir-marseille/une-ville-de-tourisme	
Minneapolis	34500000	https://www.minneapolis.org/media/news-releases/minneapolis-st-paul-sets-visitor-record-2018/	
Naples	13100000	https://luggagehero.com/blog/italy-travel-statistics/	
New York	62800000	https://www.nytimes.com/2017/11/19/nyregion/new-york-city-tourism.html	
Offa	NA	NA	Data not available

Oslo	1500000	https://www.statista.com/statistics/806411/international-tourist-arrivals-in-norway/	
Paris	30000000	https://www.condorferries.co.uk/france-tourism-statistics	
Porto	1600000	http://www.xinhuanet.com/english/2018-04/05/c_137088807.htm	
Rio de Janeiro	1300000	https://www.statista.com/statistics/976338/brazil-tourist-arrivals-destination-city/	
Sacramento	4300000	https://www.downtownsac.org/learn-about-downtown/	
San Francisco	25800000	https://www.sftravel.com/article/san-francisco-travel-reports-record-breaking-tourism-levels-2018-gives-projections-2019	
Santiago	NA	NA	Data not available
Sao Paulo	14900000	https://cidadedesaopaulo.com/v2/pqsp/dados-e-fatos/?lang=en	
Sendai			Data not available
Seoul	9110000	https://www.worlddata.info/asia/south-korea/tourism.php	
Singapore	19100000	https://www.statista.com/statistics/977993/total-international-visitor-arrivals-singapore/	
Sofia	992075	https://investsofia.com/wp-content/uploads/2019/10/Sofia-Tourism-and-Air-Transport-Market-Report-2019-ENG.pdf	
Stockholm	2600000	https://www.worlddata.info/europe/sweden/tourism.php	
Taipei	7350000	https://newsroom.mastercard.com/wp-content/uploads/2016/09/FINAL-Global-Destination-Cities-Index-Report.pdf	
Tokyo	15200000	http://www.xinhuanet.com/english/2018-04/05/c_137088807.htm	
Vienna	17600000	https://metropole.at/record-vienna-tourism/	
Yamaguchi	3435900	https://www.mlit.go.jp/common/001141407.pdf	
Zurich	2240000	https://newsroom.mastercard.com/wp-content/uploads/2016/09/FINAL-Global-Destination-Cities-Index-Report.pdf	

Supplementary Text 1: The urban, soil, and marine microbiomes

Microorganisms in the built, soil, and marine environments

Analysis of 4,135 samples collected from 53 cities in 2015-2017 by the MetaSUB project identified 3,660 taxa (Danko et al. 2021), 2,910 of which were annotated by the Microbiome Directory (Sierra et al. 2019) and GTDB (Parks et al. 2021). The overall average relative sequence abundance (RSA) of bacteria out of all the taxa found in this project was ~97%, with a minority of viruses (0.01%) and archaea (0.05%). The remaining taxa were Eukaryotes (0.97%) or undefined organisms (~1.5%), all of which were analyzed in this project. Sample richness ranged from six to 2,209 taxa. Based on sample occupancy, we defined three microbiome groups: the *common taxa* (found in more than 95% of samples) and the *rare taxa* (found in fewer than 5% of samples), with the remaining range of sample occupancy (5-95%) considered *intermediate taxa*. The common taxa (1%) accounted for 25% of all abundance, whereas the rarest taxa (~45%) had a global RSA of 0.16% (Figure S1.1A1). On average, most of the taxa found in cities are neither globally abundant nor rare and show a patchy distribution across sites, i.e., exhibit varying proportions in different localities (Figure S1.2A1).

Based on rank abundance curves and the distance-decay of community similarity, it is expected that the number of taxa shared among all samples decreases with the number of studied samples. This is because microorganisms have a patchy spatio-temporal distribution in which few microorganisms are common, while the vast majority are rare and often present in just one sample (Pascoal, Costa, and Magalhães 2020). Our findings deviated from this expectation because, using the 20% cutoff, on average, 60-80% of the samples shared more taxa than the 40-60% and 20-40% samples (Figure S1.1A1). This is most likely due to the role of humans facilitating dispersal across urban microbes, thereby altering natural distribution patterns. Among the most common taxa are *Pseudomonas putida*, *Pseudomonas aeruginosa*, and *Pseudomonas stutzeri*, all belonging to the phylum Pseudomonadota (Figure S1.3A1), alongside other common taxa belonging to the Actinobacteria and Firmicutes phyla.

Taxa counts in cities ranged widely (547-2,838). In terms of regional occupancy, 8% of the taxa were found in all cities, and 11% were found in only one of the cities (Figure S1.1A2). Hamilton (Oceania) had the highest sample average abundance of rare taxa, followed by Offa (Nigeria). Hong Kong (China) (51%), followed by Hamilton (Oceania) (30%), had the sample with the highest rare taxa abundance. The rare taxa are completely absent from 121 samples and are most prevalent in London, followed by Tokyo. Eastbourne (England) had the lowest abundance of common taxa, followed by Brisbane (Australia) (Figure S1.2A1). The RSA of city taxa differed between cities and was uncorrelated with the sample's average number of taxa (*Pearson-test*, n=53, r=0.19, p-value=0.18) (Figure S1.2A2).

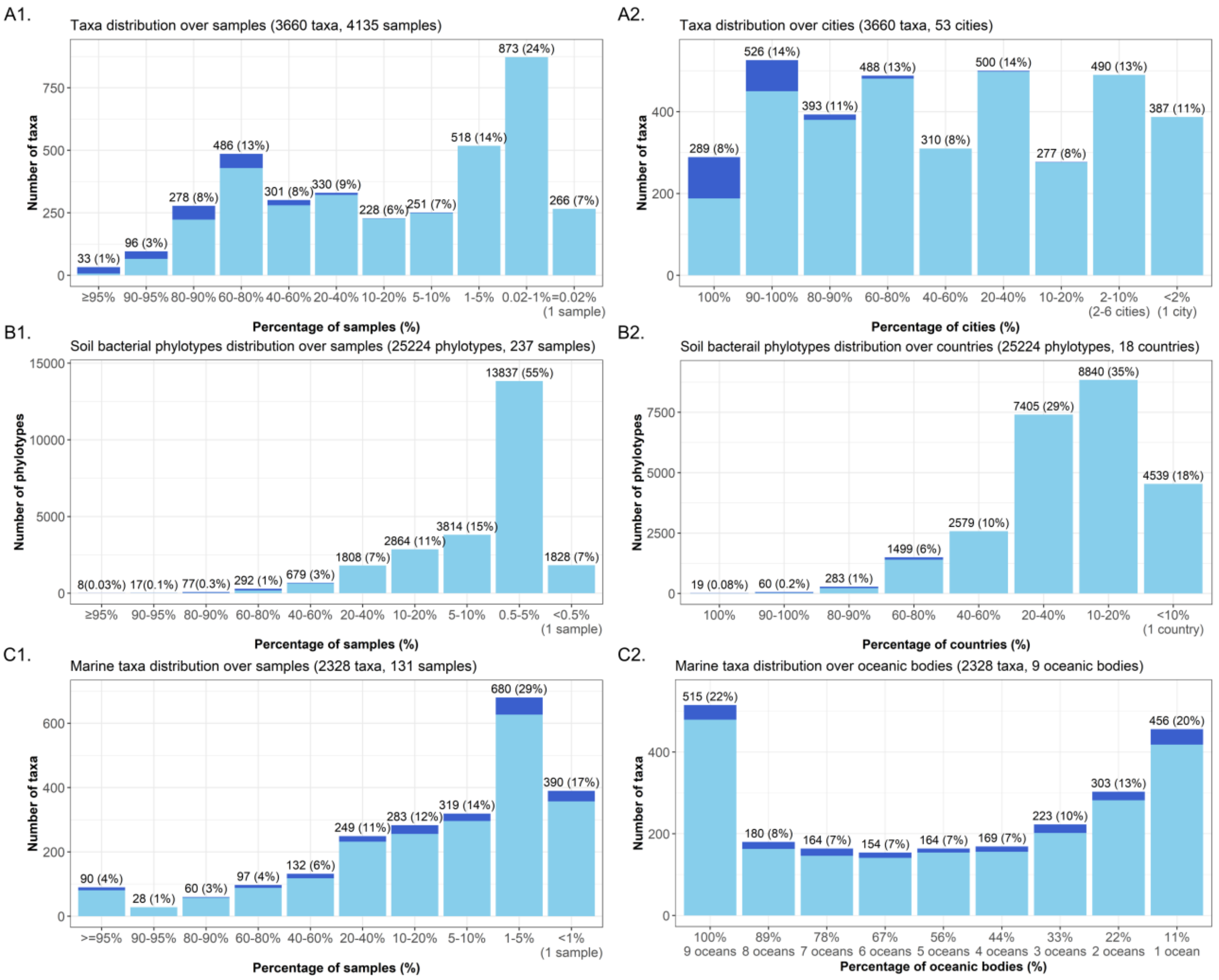

Figure S1.1. **The occupancy distribution of taxa in the MetaSUB (A), soil (B), and marine (C) datasets.** Sample occupancy plots (left) show the number and percentage of non-unique taxa per sample. Regional occupancy plots (right) show the number and percentage of non-unique taxa per region (e.g., city or country). GITs are shown by the dark blue color, whereas the light blue color marks non-GITs.

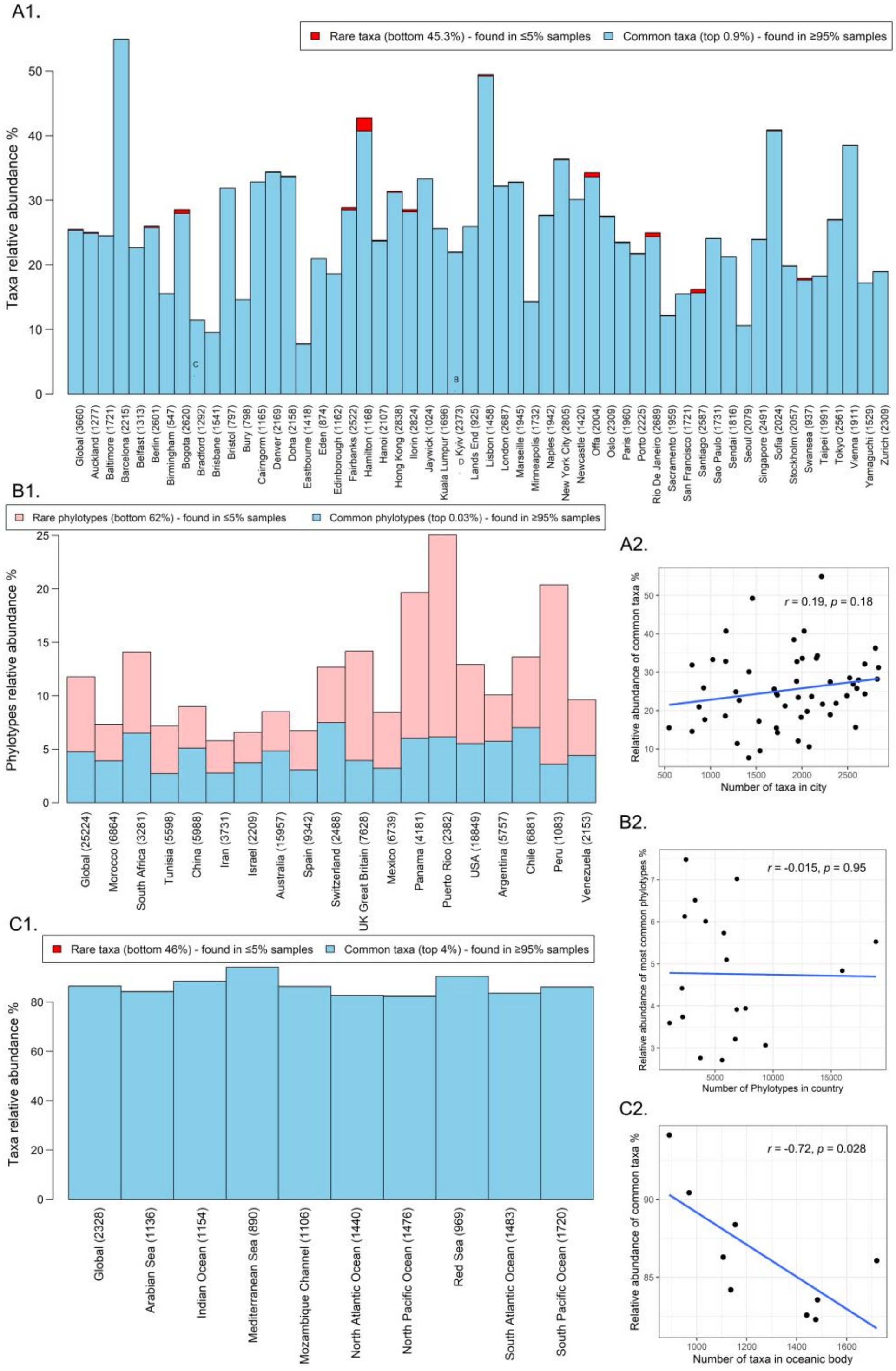

Figure S1.2. **The RSA of common and rare taxa in the MetaSUB, soil, and marine datasets.** RSA of the common and rare taxa group per city in the MetaSUB dataset (A1) per country in the soil dataset (B1) and per oceanic water body in the marine dataset (C1). The correlation of common taxa RSA and the number of taxa for cities in the MetaSUB dataset (*Pearson-test*, *n*=53, *r*=0.19, *p*-value=0.18) (A2), for countries in the soil dataset (*Pearson-test*, *n*=18, *r*=-0.015, *p*-value=0.95) (B2), and for oceanic water bodies in the marine dataset (*Pearson-test*, *n*=9, *r*=-0.72, *p*-value=0.028) (C2).

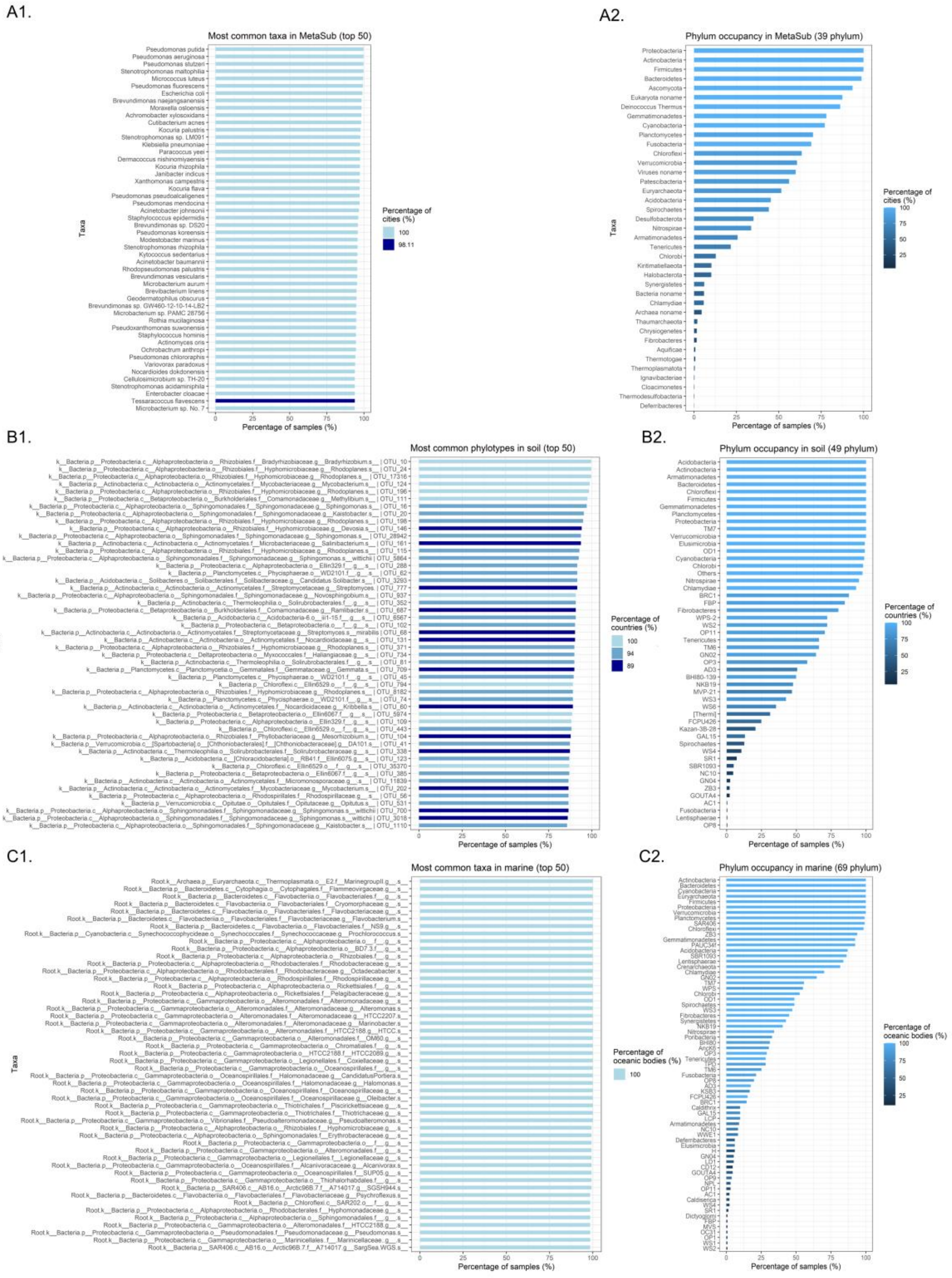

Figure S1.3. **Microbial diversity in the MetaSUB (A), soil (B), and marine (C) datasets.** Plots show the occupancy distribution of the most common taxa in samples (left) and the occupancy of taxa at the phylum level (right). Row colors and lengths represent the regional occupancy of the taxon/phylum in the cities/soil regions/oceanic water bodies and the sample occupancy in all samples in each dataset, respectively. The synonym of Proteobacteria phylum is Pseudomonadota phylum.

The soil dataset comprised 237 samples from 18 countries across six continents (Delgado-Baquerizo et al. 2018). Classified using the Operational Taxonomic Unit (OTU), 25,224 bacterial phylotypes were identified. With global RSAs of 4.8%, only a small proportion (0.03%) of the phylotypes were common taxa. Rare phylotypes accounted for more than 60% of the phylotypes, with a total RSA of 7%. Furthermore, 7% of phylotypes were extremely uncommon, appearing in only one sample (Figure S1.1B1). Only 19 phylotypes (less than 1%) were shared across all countries, whereas 18% were country-specific (Figure S1.1B2). The most common soil phylotypes were Baradyrhizobium, Rhodoplanes, and Mycobacterium, all of which were identified on genus level and belonged to the Pseudomonadota phylum (Figure S1.3B1). 11 phyla were shared in all samples, and only the WS2 phylum was not ubiquitous among the reported soil dominant phylotypes and just shared in 72% of the samples (Figure S1.3B2). With 18,849 phylotypes, the United States has the highest microbiome richness, while Peru has the lowest, with just 1,083 phylotypes. Unlike in the urban and marine environments (Figure S1.2A1, S1.2C1), in soil, common phylotypes did not dominate the RSA (Figure S1.2B1). The largest RSA of rare phylotypes was found in Puerto Rico, followed by Peru. Israel, Iran, and Morocco had the lowest abundance of rare phylotypes (Figure S1.2B1). No correlation existed between the number of phylotypes and the RSA of the common phylotypes in the country (*Pearson-test*, n=18, r=-0.015, p-value=0.95) (Figure S1.2B2).

The Tara Oceans dataset includes 2,328 taxa (average RSA of 95% bacteria and 5% archaea) from 9 oceanic water bodies with non-zero abundance (Sunagawa et al., 2015). Taxa richness in the samples ranges from 204 to 845. The common taxa group accounts for 4%, with an average RSA of 86%. By contrast, the rare taxa account for 46%, with an average RSA of 0.05% (Figure S1.1C1). Marine microbes with higher occupancy were more abundant than urban and soil microbes. Some taxa (33) appeared in all the samples, and 22% of taxa were shared in all oceanic water bodies (Figure S1.1C2). Actinobacteria, Bacteroidetes, and Cyanobacteria are the most common phylum, and all are widely distributed in both soil and marine (Figure S1.3C2). Oceanic water bodies had a taxa count ranging from 890 (Mediterranean Sea) to 1,720 (South Pacific Ocean), with 6% of the taxa found in one oceanic water body. The Arabian Sea had the most abundant rare taxa, and the North Atlantic Ocean had the least abundant rare taxa (Figure S1.2C1). The RSAs of taxa differed by oceanic water bodies, and the total global average RSA of the common taxa group was correlated with taxa number per city (*Pearson-test*, n=9, r=-0.72, p-value=0.028) (Figure S1.2C2). The difference in the RSA of the common microorganisms between different oceanic water bodies was less than that between different cities in the MetaSUB. This might be because microorganisms circulate and migrate more easily in water than on land.

Pathogenic microorganisms in the built, soil, and marine environments

Pathogens are highly informative GITs (Supplementary Text 2). Assessing pathogenicity, we found 310 pathogens in the MetaSUB dataset: 222 animals and 73 plant pathogens. Fifteen pathogens affected both kingdoms. Pathogens occupancy distribution followed the taxa distribution, i.e., only 2% of the pathogens were found in over 95% of samples, whereas 39% were found in less than 5% of samples (Figure S1.4A1). Moreover, over a third of all the pathogens were found in 20-80% of all samples. The most common pathogens were *Pseudomonas aeruginosa*, *Stenotrophomonas maltophilia*, and *Pseudomonas fluorescens* (Figure S1.5A1), all of which are gram-negative bacteria and are infectious to humans. Amongst these are opportunistic pathogens. By contrast, the 17 rarest pathogens were distributed across an equal number of samples (Figure S1.5A2). Animal pathogens were the most frequent pathogens, both in counts and global average RSA (Figure S1.2A1B1). All the cities harbored the same 11% pathogens, whereas 10% were found only in one of the cities. This pattern deviated from what we observed per taxa (Figure S1.1A2), as more pathogens were shared across cities (Figure S1.4A2). Barcelona hosted the highest pathogen abundance, followed by several sites in England (Figure S1.6A S1.8A). The RSAs of city pathogens

differed between cities and were moderately negatively correlated with the number of pathogens per city (*Pearson-test*, *n*=53, *r*=-0.45, *p*-value=0.00078) (Figure S1.6C).

The soil dataset included 248 species-level phylotypes, 62 of which were annotated by the Microbiome Directory. Of those, seven OTUs were pathogenic: four were animal, and two were plant pathogens. The remaining one *Serratia marcescens* affected both animals and plants (Figure S1.5B). *Pseudomonas vindiflava*, a plant pathogen that causes bacterial blight in Kiwifruit, was the most frequent pathogen phylotype, followed by *Stenotrophomonas maltophilia*, which can infect humans. No pathogen phylotypes were shared in all countries. The national average RSA of pathogens was 0.055%, and there were no continental characteristics in the abundance of pathogens. Panama had the highest RSA of pathogens (0.4%), followed by the United Kingdom (0.1%) (Figure S1.7B). No pathogen phylotypes were found in Switzerland (one sample), Israel (one sample), Peru (one sample), South Africa three samples), and Tunisia (six samples), which is likely due to their small sample size and undersampling of pathogens in those regions. For instance, in the first microbiome survey of the study archeological site of Tel Megiddo in Israel, Zhang et al. (2023) identified soil pathogens. The number of soil pathogens moderately positively correlated with their RSA in each country after removing one outlier (Panama). (*Pearson-test*, *n*=17, *r*=0.49, *p*-value=0.044) (Figure S1.8).

In the marine dataset, 50 pathogens were found and annotated by Microbiome Directory: 37 animal and 10 plant pathogens. Three additional pathogens infect both hosts. Pathogens were found in substantially lower sample occupancy in marine samples than in urban ones. Only one animal pathogen was present in 67% of samples, and the rest of the pathogens were present in less than 45% of samples (Figure S1.4B1). More than one-fourth of the pathogens were present in one sample. *Cylindrospermopsis raciborskii* (animal pathogen), *Salipiger mucosus* (plant pathogen), and *Orientia tsutsugamushi* (animal pathogen) were the most frequent infections (Figure S1.5C1). Marine pathogens had a relatively low average abundance (~0.02%), with animal pathogens being the most abundant. Among nine oceanic water bodies, six pathogens were ubiquitous, whereas 16 pathogens were unique to a specific oceanic water body (Figure S1.4B2). Samples with a high RSA of pathogens did not appear to be enriched in specific areas, and their distribution was more scattered (Figure S1.7C). Overall, the South Atlantic Ocean had the highest pathogen average RSA, while the Arabian Sea had the lowest. (Figure S1.6B) The pathogen abundance value of an oceanic water body was independent of the number of pathogens it contains (*Pearson-test*, *n*=9, *r*=0.32, *p*-value=0.4) (Figure S1.6D).

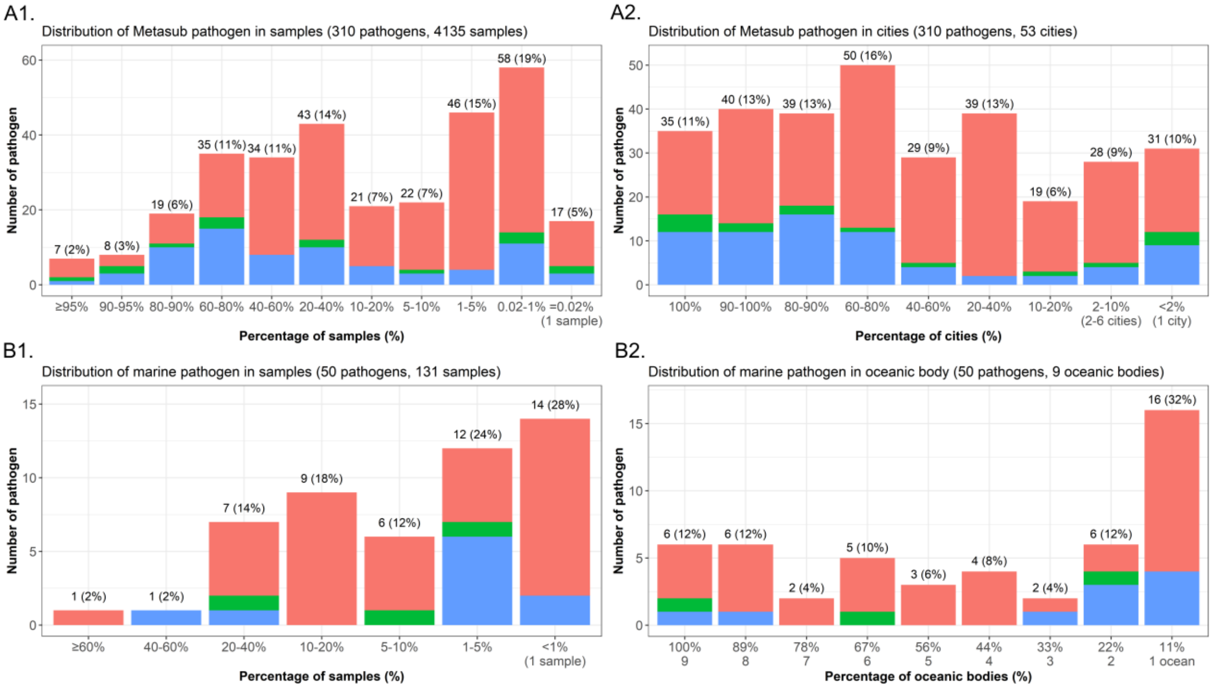

Figure S1.4. **The occupancy distribution of 310 and 50 pathogens in the MetaSUB (A) and marine (B) datasets, respectively.** Plots show the sample occupancy (left) as the number and percentage of pathogens in samples and regional occupancy (right) as the number and percentage of pathogenes across cities and oceanic water bodies. Pathogens are color-coded by their animal (red), plant (blue), and both animal and plant (green) hosts.

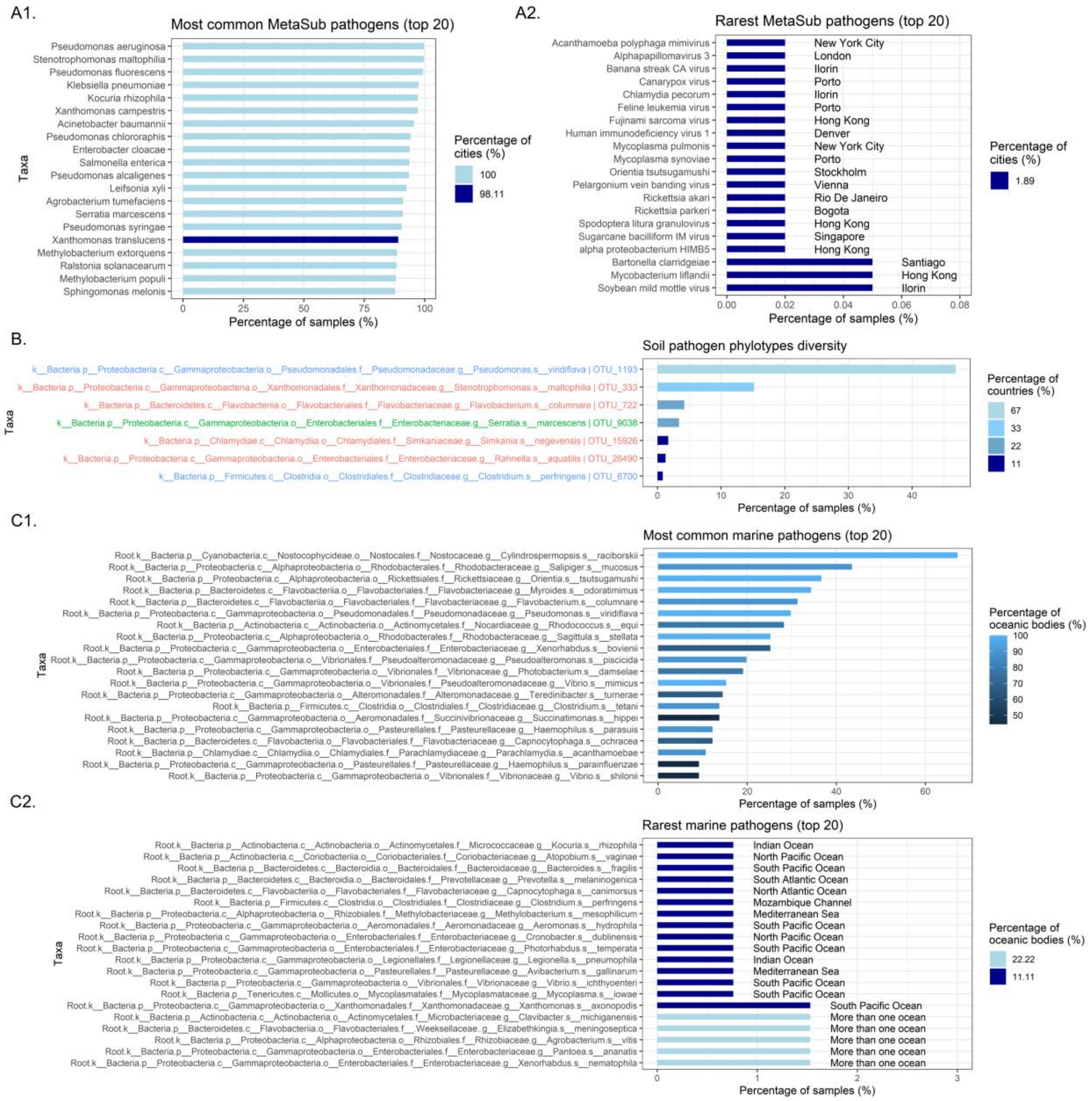

Figure S1.5. **Pathogen diversity in the MetaSUB (A), soil (B), and marine dataset (C).** The distribution of the most common and rarest pathogens in the MetaSUB (A) and marine (C) datasets. (B) The distribution of pathogens in the soil dataset. The text color in (B) represents the pathogen types: animal pathogen (red), plant pathogen (blue), and pathogen infecting both kingdoms (green). Row colors and lengths represent the regional occupancy of the pathogens in the city/soil region/oceanic water body and the sample occupancy in all samples.

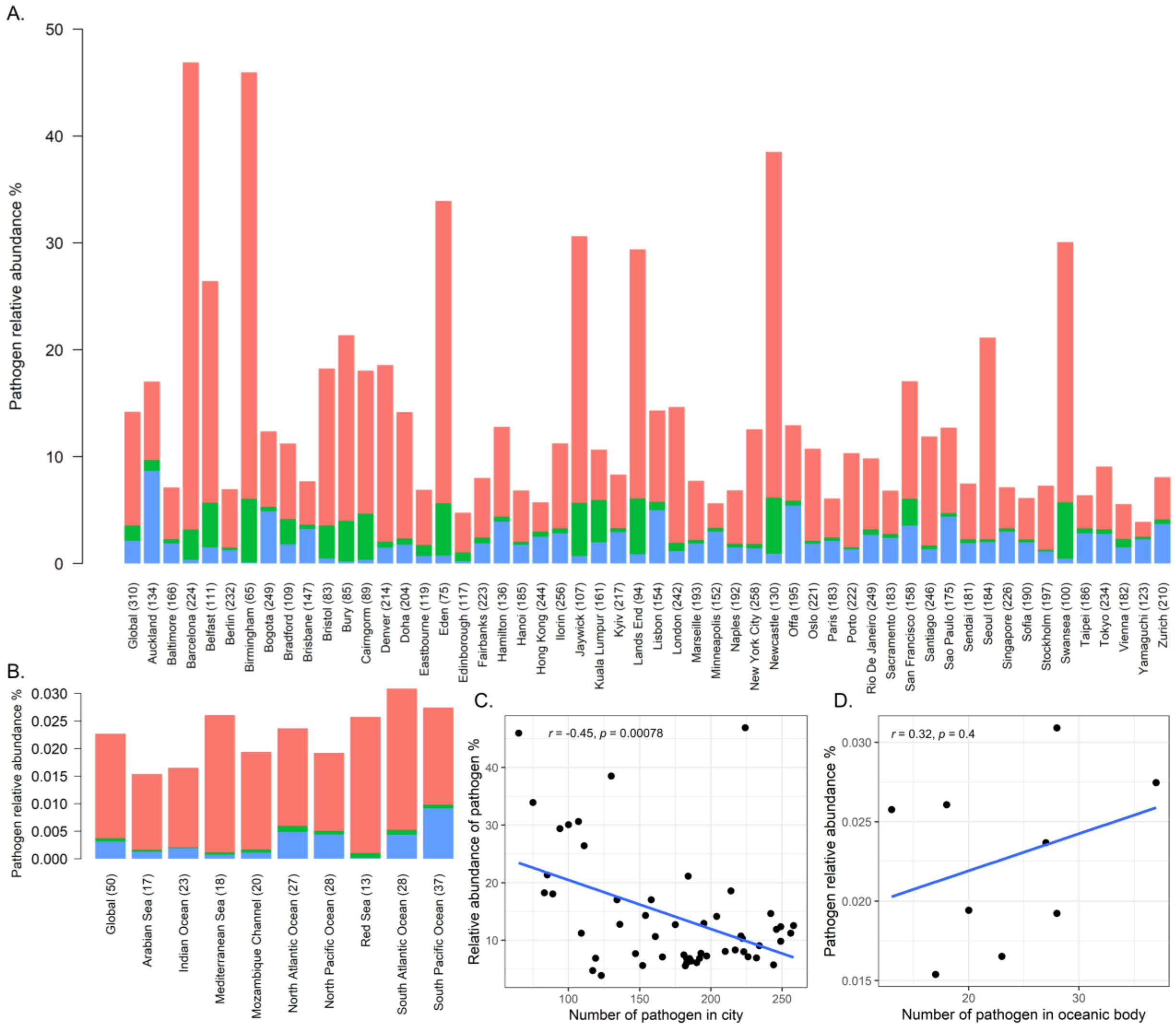

Figure S1.6. **The RSAs of 310 pathogens in the MetaSUB dataset and 50 pathogens in the marine dataset.** The relative sequence abundance of animal and plant pathogens per city (A) and per oceanic water body (B). Pathogens are color-coded by their animal (red), plant (blue), and both animal and plant (green) hosts. The correlation of pathogen number and RSA (%) were calculated for the MetaSUB (*Pearson-test*, *n*=53, *r*=-0.45, *p*-value=0.00078) (C) and marine (*Pearson-test*, *n*=9, *r*=0.32, *p*-value=0.4) (D) datasets.

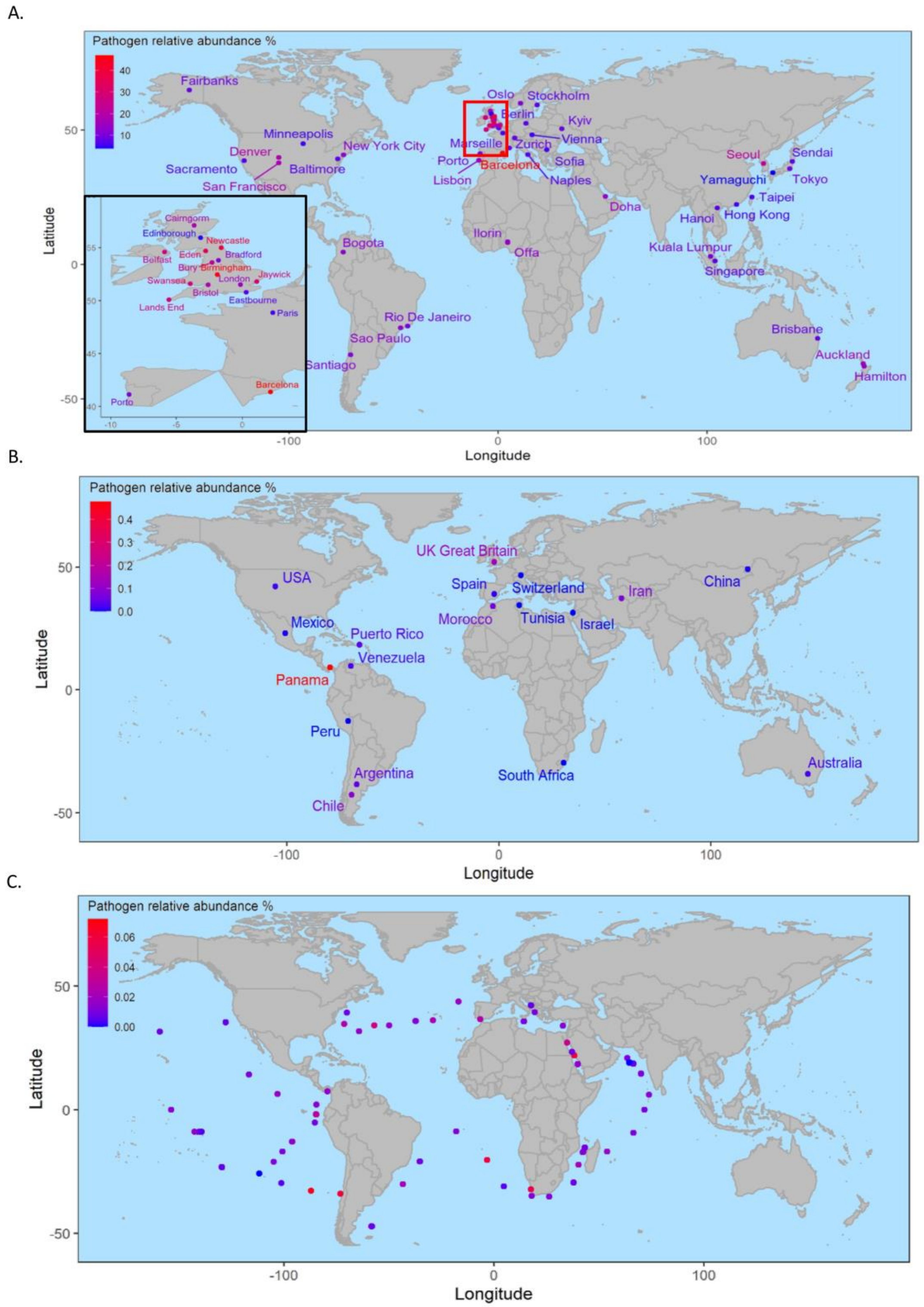

Figure S1.7. **Pathogen global biodiversity in the MetaSUB, soil, and marine datasets.** (A) The RSA of pathogens per city in MetaSUB. Inset shows England. (B) The RSA of pathogens per country in the soil dataset. (C) The RSA of pathogens per sample in the marine dataset. Sampling sites are marked in dots, and their color reflects the RSA of the pathogens.

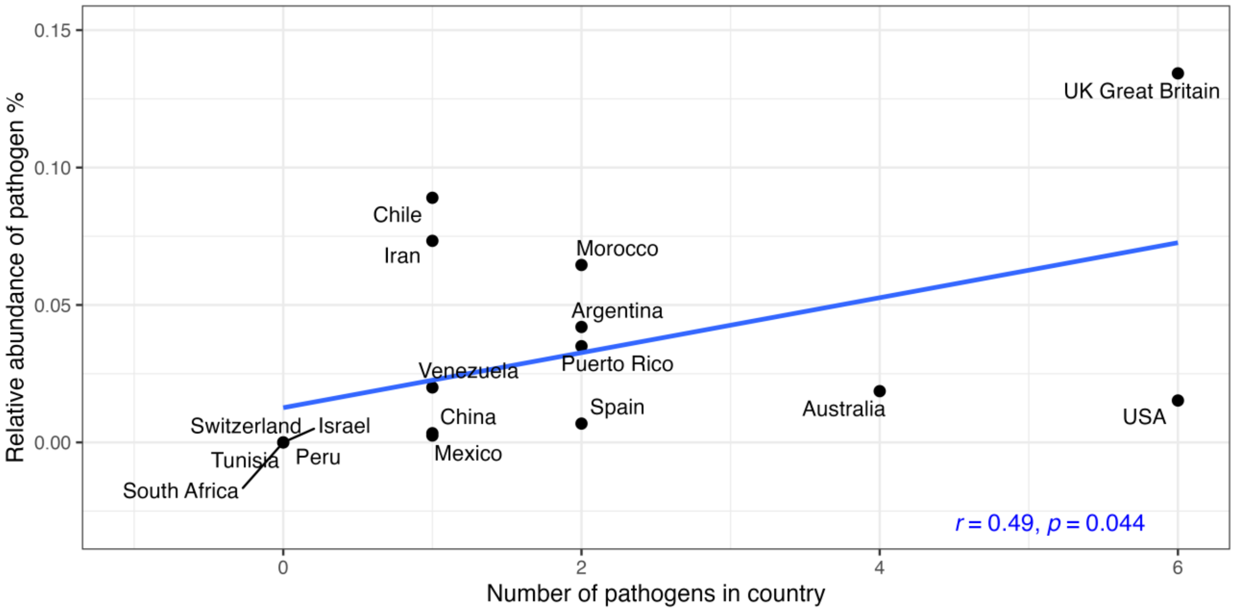

Figure S1.8. **The correlation of pathogen number and RSA (%) for countries in the soil dataset.** The number of pathogens in Switzerland, Israel, Peru, South Africa, and Tunisia is zero. There is a slight linear positive correlation between the relative abundance of pathogens and their numbers in countries (*Pearson-test*, n=17, r=0.49, p-value=0.044).

Supplementary Text 2: Characterizing the GITs used for biolocalization

The hallmarks of GITs

We focused on the MetaSUB dataset due to its large size and high taxa biodiversity to further analyze the 200 GITs (features) used by mGPS to biolocalize the samples. First, we investigated the association between GITs occupancy and their RSA. GIT occupancy was correlated with the log global RSA value (*T-test*, n=200, r=0.77, p-value<2.2e-16), with GITs of greater occupancy having larger global RSA (Figure S2.1A). Second, GITs were found to have a greater global RSA than non-GITs, with the more informative ones (i.e., ranked by *feature importance* as the most informative taxa for biogeography) for biogeographical predictions having the highest global RSA (Figure S2.1B).

This finding holds for both presence in sample and sampling sites, with the most informative GITs found in almost all the studied locations (Figure S2.1C-D). In other words, GITs are more ubiquitous compared with non-GITs, which allows their utility as *markers of geography*. This finding may appear counterintuitive, as GITs may be imagined as unique to cities. However, while there are unique taxa to cities, they do not appear in all the samples of that city and cannot be used as geographical markers of those cities. In addition, the predictability of such a tool would be questionable as it would be highly specific to the studied sites by design. Due to the sampling sites, those taxa likely appear unique to those cities (and transit systems). By contrast, selecting globally abundant taxa with high regional variation is more effective (Figures S5-6 and S8). As such, Pseudomonadota, Actinobacteria, and Firmicutes were the most abundant GIT phylum (Figure S2.2B). While Gammaproteobacteria, Actinomycetia, and Alphaproteobacteria were the most abundant GIT class (Figure S2.2C). As expected, the RSAs of GITs varied by city, with Barcelona having the highest GIT abundance (Figure S2.2A).

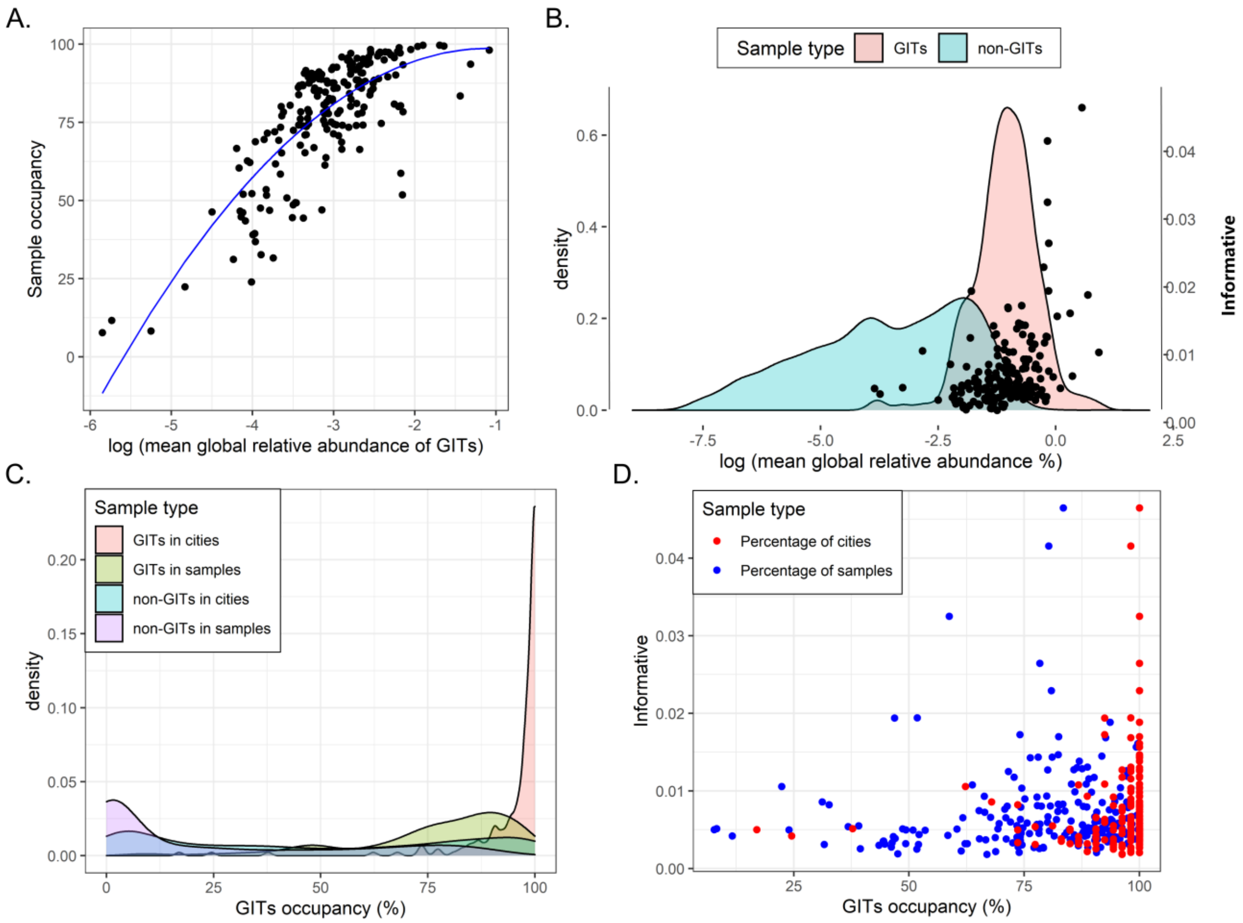

Figure S2.1. **Comparing the biodiversity of GIT and non-GIT taxa.** (A) GIT occupancy versus log(global RSA). (B) The line plot shows the probability density function (left y-axis) of the RSA (log scale) of GIT (pink) and non-GIT (green) taxa. The dot plot shows the relationship between the GIT RSA (black dots) and their importance value (right y-axis). (C) The distribution of sample occupancy and regional occupancy of GIT and non-GIT taxa. Higher density reflects a higher number of taxa. (D) The relationship between the GITs sample occupancy (blue) and regional occupancy (red) per their importance value (see methods).

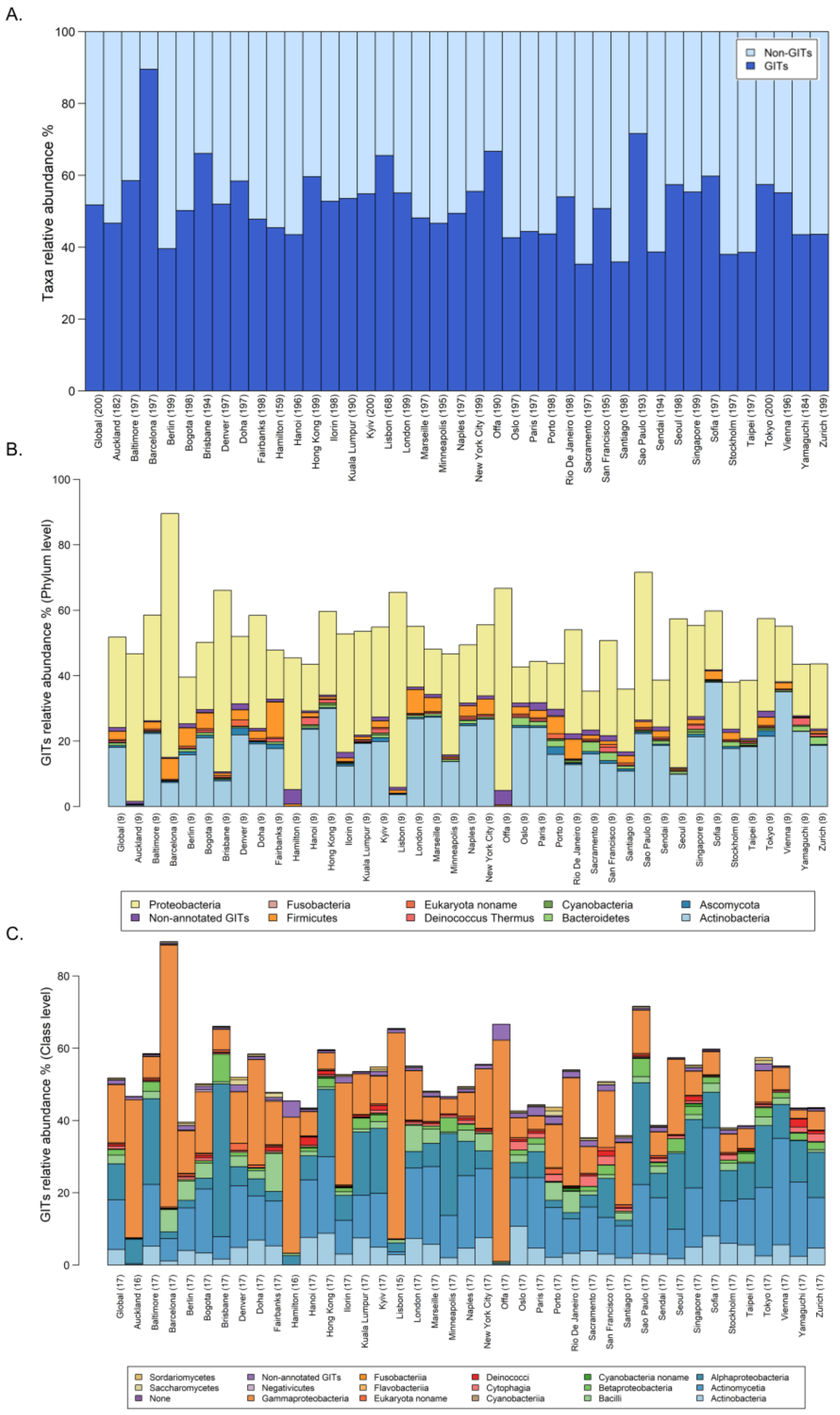

Figure S2.2. **RSA of GITs and non-GITs per city.** GITs and non-GITs RSA comparison per city (A). GITs are classified on phylum level (B) and class level (C). Colors represent different GIT types, with the remainder representing non-GITs (not shown). The synonym of Proteobacteria phylum is Pseudomonadota phylum.

The abundance of pathogenic GITs

Of the 200 MetaSUB GITs, 25 were pathogens (17 animal pathogens, nine plant pathogens, and one pathogen common to both kingdoms). Each city had 22-25 pathogenic GITs. Although pathogenic GITs represent one-eighth of the pathogenic taxa, their total RSA exceeded 50% of all pathogenic taxa. In other words, mGPS considers pathogen abundance an essential feature in predicting the origin of samples. The median pathogen RSA per city is 5% and does not vary with geography or the proportion of pathogenic GITs (Figure S2.3A). Contrary to the expectation, city population size is not associated with the pathogen's total RSA and alpha diversity, implying that larger cities do not contain a higher diversity of infectious agents than smaller cities (Figure S2.3B-C and S2.4).

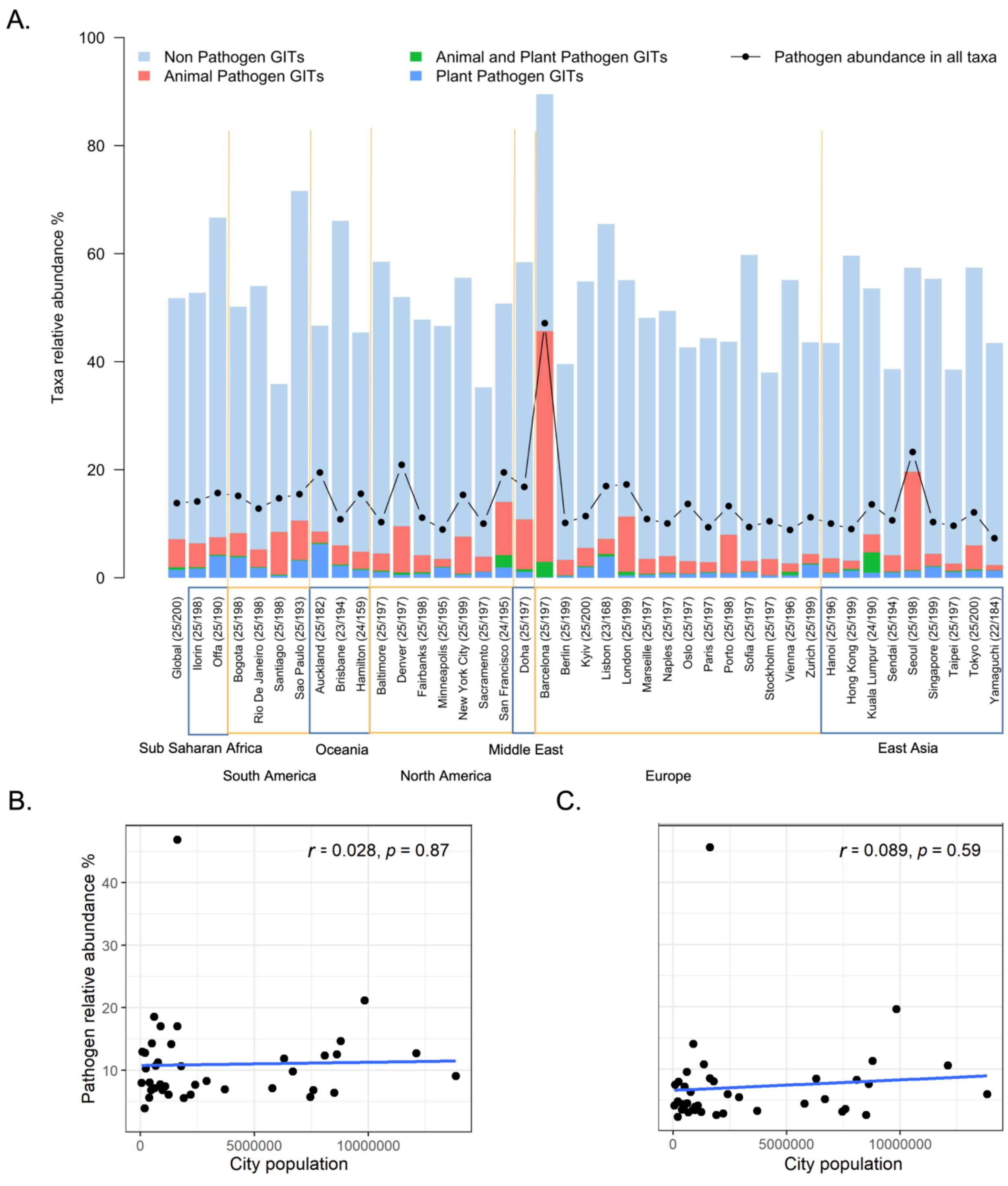

Figure S2.3. **The distribution of pathogenic GITs.** (A) The RSA of pathogenic and non-pathogenic GITs per city. Colors represent different types of pathogens, with the remainder representing non-GITs (not shown). Cities are sorted by region. The global RSA of pathogens is shown. The relationship between the urban population and the RSA of all pathogens (*Pearson-test*, *n*=40, *r*=0.028, *p*-value=0.87) (B) or GIT pathogen (*Pearson-test*, *n*=40, *r*=0.089, *p*-value=0.59) (C).

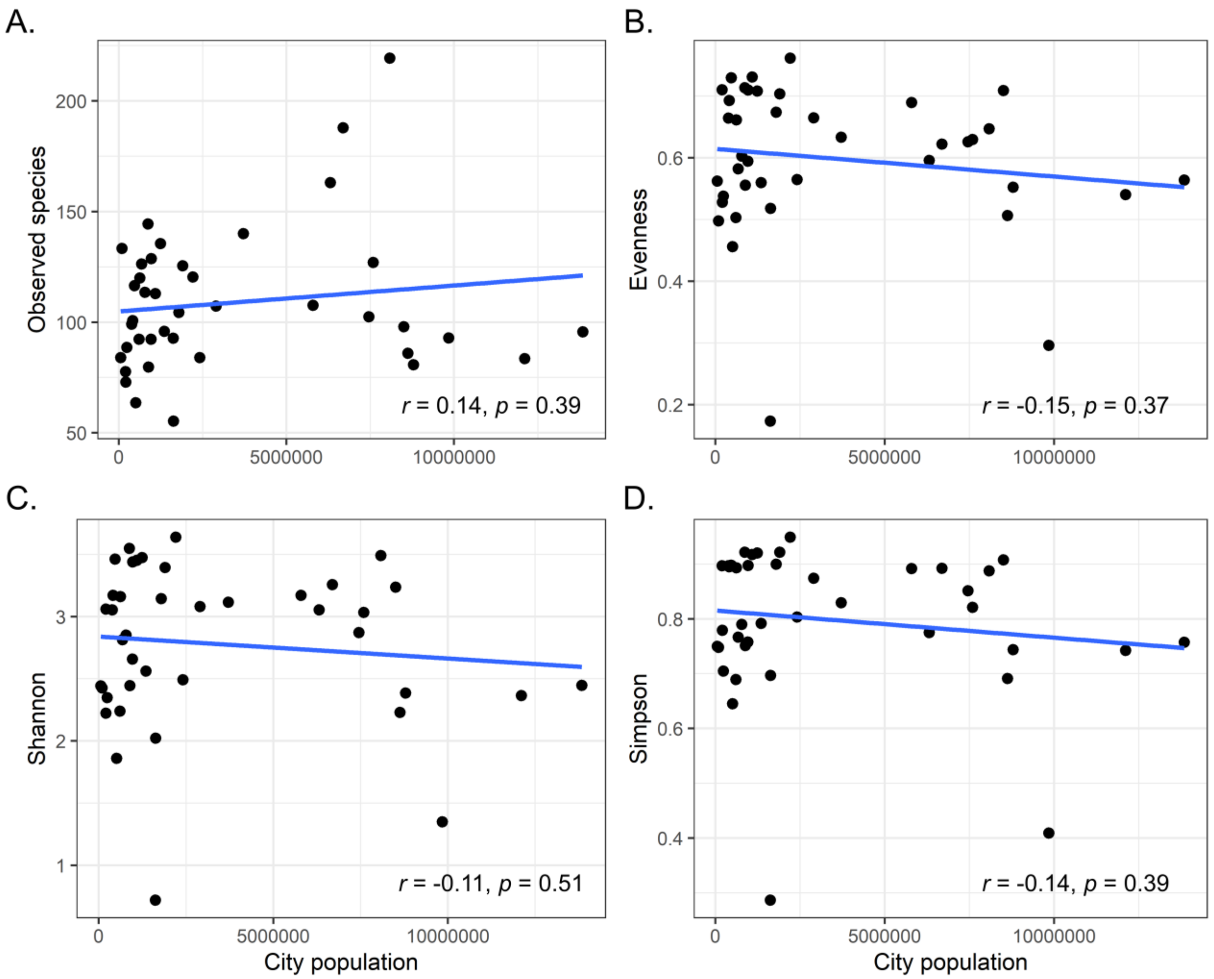

Figure S2.4 **Correlation between alpha diversity of 310 pathogens and city populations in the MetaSUB dataset.** The average observed species number (*Pearson-test*, *n*=40, *r*=0.14, *p*-value=0.39) (A), evenness index (*Pearson-test*, *n*=40, *r*=-0.15, *p*-value=0.37) (B), Shannon index (*Pearson-test*, *n*=40, *r*=-0.11, *p*-value=0.51) (C), and Gini-Simpson index (*Pearson-test*, *n*=40, *r*=-0.14, *p*-value=0.39) (D) of pathogen in each city versus city population. Contrary to the expectation, city population size is not associated with the pathogen total RSA and alpha diversity, implying that larger cities do not contain a higher diversity of infectious agents than smaller cities (Figure S2.3B-C and S2.4).

Supplementary Text 3: Distinguishing local and non-local taxa using mGPS

mGPS localizes samples to source communities

Differentiating local from non-local taxa is a key strength of mGPS as it allows for comparisons between the two cohorts to be carried out. To test if mGPS bio traces microorganisms to their source sites, we compared the RSA distribution of geographically informative taxa (GIT) in mGPS predicted sites to the distributions of local and non-local taxa. Our rationale was that samples harbor different taxa, some of which function as markers of geography and can indicate the geographical source of the sample when the sample is non-local. Analyzing the GITs of the test sample may show a significant difference in their RSA distribution between the sampling site and the predicted site (Figure 7). If the RSA of the non-local GITs is more similar to that of GITs in their mGPS predicted site (orange) than to GITs in their sampling site (green), it confirms their non-local status and source site. For example, a GIT affiliating with *Salmonella enterica* sampled in Europe (*sampling site*) was predicted in North America (*predicted site*). To test the reliability of this prediction, we compared the RSA distributions of GITs from both sites to that of *S. enterica* (Figure S3.1A1) and showed that *S. enterica* exhibits a significantly different distribution than European GITs but a similar distribution to North American GITs. Moreover, the RSAs of European GITs significantly differ from the distribution observed in RSAs of North American GITs. Searching this pattern for GITs across the three main regions using the 15 most informative GITs (Figure S3.1), we confirmed it in 8.7 GITs, on average, which demonstrates the validity of mGPS predictions rather than erroneous assignment. The remaining cases that could not be confirmed were likely because the particular GITs tested were not well represented in the tested target site. This does not affect the accuracy of mGPS predictions because mGPS considers all GITs.

We further evaluated two negative controls. First, we shuffled the mGPS predicted non-local samples with the local samples, then randomly selected 20 samples and repeated the comparison of GITs RSA with GITs from the sampling and predicted sites (Figures S3.1A2, S3.1B2, S3.1C2). We show that due to the dilution of the non-locally predicted GITs, the mixed samples were not significantly different from both sites or had a more similar distribution to the sampling site, contrastingly with the original scheme (Figures S3.1A1, S3.1B1, S3.1C1). Second, to test how often the segregation pattern appears for non-GIT taxa, we randomly selected 15 non-GITs and tested them in the aforementioned scheme. We repeated the analysis 1,000 times for three regions. On average, out of 15 non-GITs, we observed the pattern in 2.6 non-GITs. Our findings thereby demonstrate that GITs are more informative than non-GITs for biogeographical predictions and that mGPS can distinguish local and non-local samples.

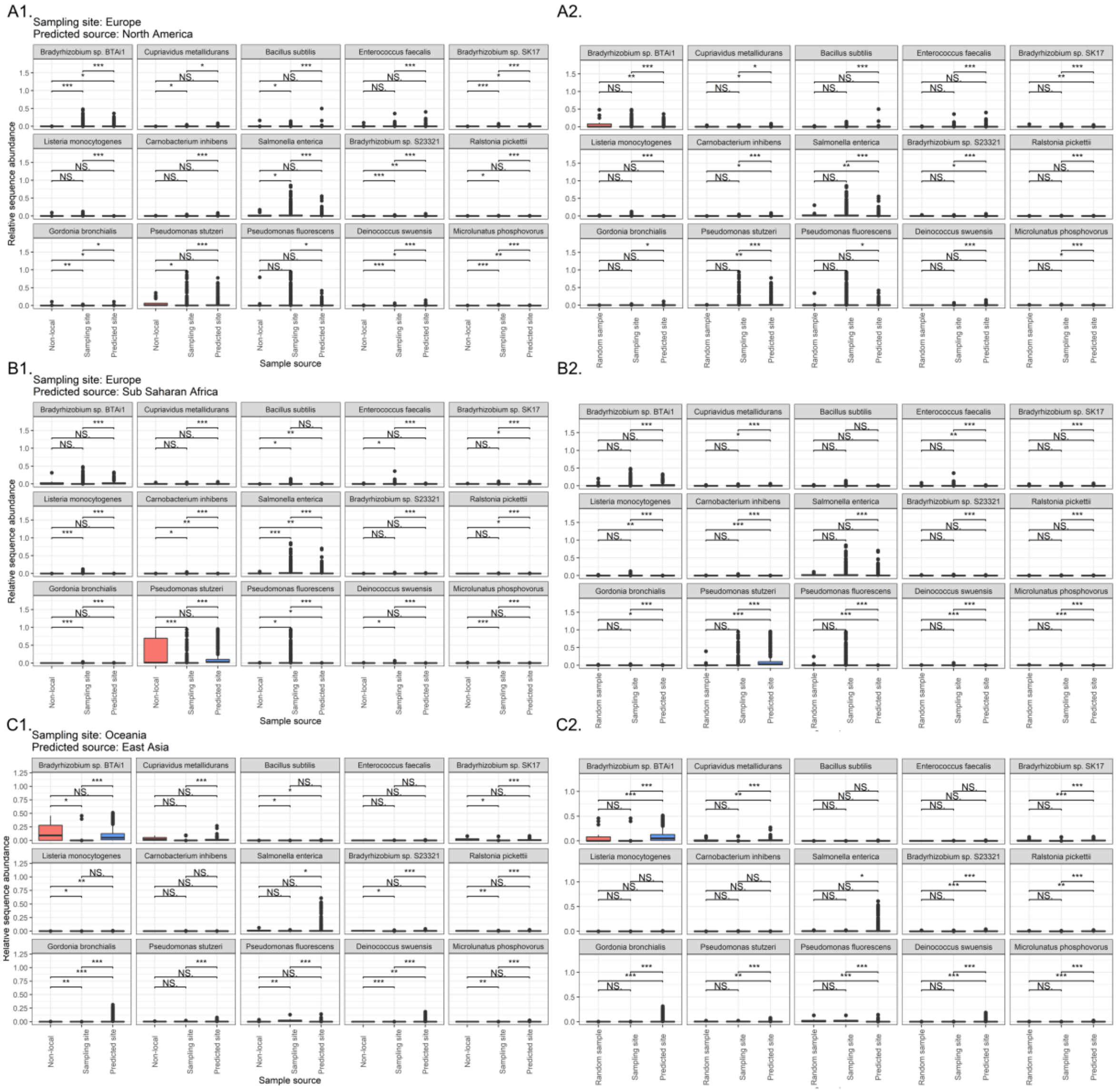

Figure S3.1. **Comparisons of RSA distributions of the 15 most informative GITs predicted non-locally with the respective distributions from their sampling, and mGPS predicted sites across three sampling sites.** Each figure pair (rows) shows 15 GITs (squares) predicted by mGPS to have migrated from outside the continent, with the left figures analyzing the GITs from the test samples and the right ones GITs from random samples. To evaluate the European and Oceanian GITs predicted to source communities in North America, Sub-Saharan Africa, and East Asia, we compared their RSA distributions to those of local GITs from all 1,264, 1,264, and 23 samples, respectively, with all 24, 21, and 10 samples from the predicted sites, respectively. In each subfigure (A-C), the RSA distributions of the non-local GITs (left) or randomly selected GITs (right) are compared to those of local GITs (*sampling site*) and GITs from the *predicted site*. For example, consider *Bradyrhizobium sp. BTAi1s*, predicted as a non-local GIT (A1). Its RSA distribution is significantly more different than the RSA distribution of local GITs in the sampling site (Europe) compared to the local GITs in the predicted site (North America). By contrast, the RSA of *Bradyrhizobium sp. BTAi1s* (A2) exhibit no significant difference compared to the RSA distribution of the local GITs in the sampling (Europe) or predicted site (North America), although the local GITs from the two sites have different RSAs. Significance was assessed using the Wilcoxon signed-rank test with the *p*-value marked in the plots as 0-0.001***, 0.001-0.01**, 0.01-0.05* or NS (non-significant difference).

Rank abundance curve of local and non-local samples differentiated by mGPS

Rank abundance curves (RAC) show the species richness and evenness, a meaningful index for the microbial environment. To find the difference between local and non-local samples differentiated by mGPS, firstly, we calculated the RAC of these two cohorts on the global level. The smallest non-local sample count was found in the Middle East, while the highest number of exotic samples was found in Oceania. Globally, non-local samples are fewer than the local ones, whereas the trend of their RACs has no significant difference (Figure S3.3). On the regional level, Europe, the Middle East, Oceania, North America, and sub-Saharan Africa show a significant difference in RAC trend between local and non-local samples, except for East Asia and South America (Figure S3.2). The number of microorganisms varied by region. The regions with higher taxa richness were less affected by the microorganisms introduced by the foreign samples, resulting in a smaller difference in the RAC trend between the two cohorts. Oceania has the lowest taxa richness (*x*-axis), followed by the Middle East, potentially explaining the different RAC trends between local and non-local taxa.

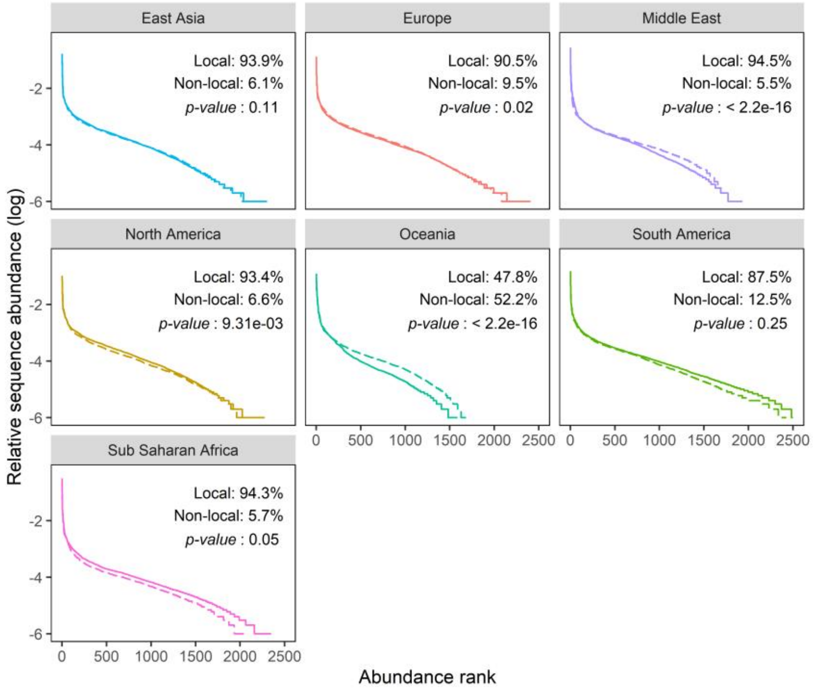

Figure S3.2. **Rank abundance curves of local and non-local samples per region.** Each subplot shows the percentage of samples predicted locally and non-locally and the *p-value* of the Kolmogorov–Smirnov test comparing the two RAC distributions. Linetype distinguishes local (solid line) and non-local (dotted line) samples.

We repeated the analysis locally, testing the RACs per city, and summarized them according to regions (Figure S3.3). Taxa richness and uniformity varied by city, even for neighboring ones. In European cities, excluding Paris and Zurich, the non-local samples contained fewer taxa than the local ones. Only in Kyiv and Paris were the RACs for local and non-local samples not significantly different. In all the Middle East, Sub-Saharan Africa, North America, and South America cities, we observed fewer taxa in the non-local samples than in local ones and significant differences between the RAC trends of the two cohorts. Interestingly, Santiago (South America) exhibited the most different RAC trends for local and non-local samples. Oceanian cities were unique in having the lowest microbial richness of all cities. Moreover, unlike in other cities, the non-local samples from Auckland and Brisbane had higher microbial richness and

uniformity than the local samples. Yamaguchi (Japan) had the most incoming migrant microorganisms. This is likely because Yamaguchi is a small region with three sides surrounded by the sea and is a tourist attraction. Indeed, the non-local samples were predicted to be the main East Asian cities, such as Tokyo and Hong Kong.

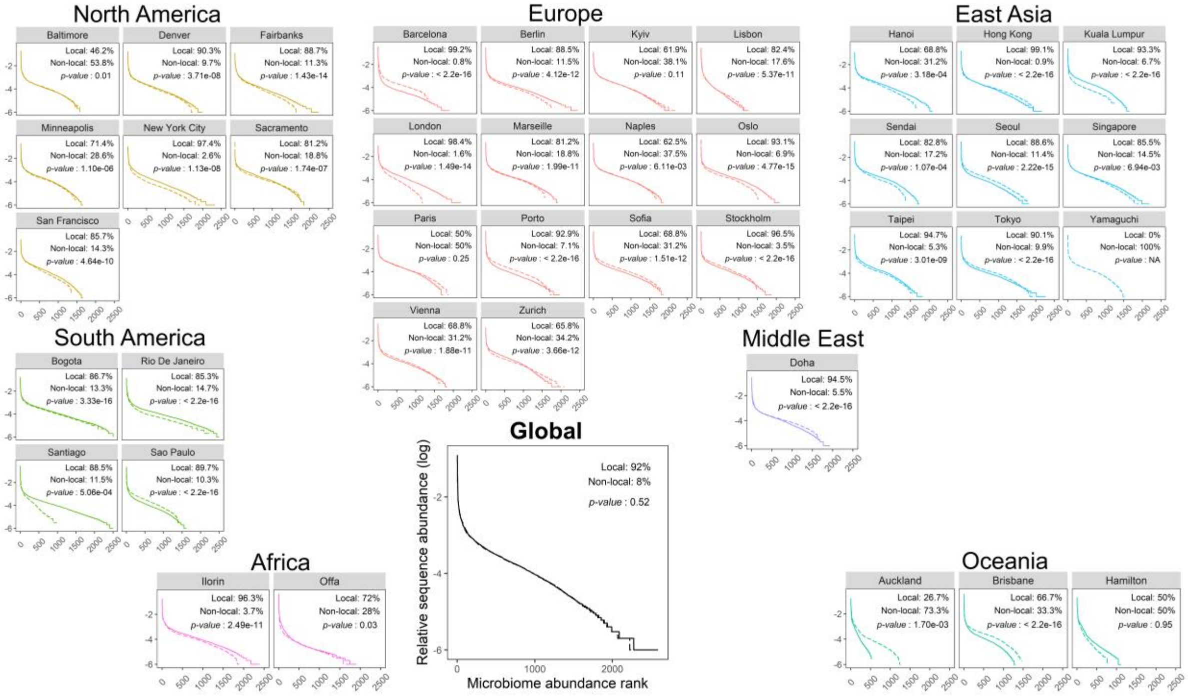

Figure S3.3. **Rank abundance curves of local and non-local samples per city.** The text in the plot means the percentage of samples that are predicted as local or non-local samples (migrated samples). Cities are separated by region. The *p-value* was obtained from the Kolmogorov–Smirnov test. Linetype distinguishes local (solid line) and non-local (dotted line) samples.

REFERENCES

Danko D, et al. 2021. A global metagenomic map of urban microbiomes and antimicrobial resistance. Cell. 184:3376-3393.e3317.

Delgado-Baquerizo M, et al. 2018. A global atlas of the dominant bacteria found in soil. Science. 359:320-325.

Parks DH, et al. 2021. GTDB: an ongoing census of bacterial and archaeal diversity through a phylogenetically consistent, rank normalized and complete genome-based taxonomy. Nucleic Acids Res. 50:D785-D794.

Pascoal F, et al. 2020. The microbial rare biosphere: current concepts, methods and ecological principles. FEMS Microbiology Ecology. 97.

Sierra MA, et al. 2019. The Microbe Directory v2.0: An Expanded Database of Ecological and Phenotypical Features of Microbes. Cold Spring Harbor Laboratory.

Zhang Y, et al. 2023. The microbial biodiversity at the archeological site of Tel Megiddo (Israel). Front. microbiol. 14:1253371.
